# Mechanistic PK-PD model of alendronate treatment of postmenopausal osteoporosis predicts bone site-specific response

**DOI:** 10.3389/fbioe.2022.940620

**Published:** 2022-08-17

**Authors:** José L. Calvo-Gallego, Peter Pivonka, Rocío Ruiz-Lozano, Javier Martínez-Reina

**Affiliations:** ^1^ Departamento de Ingeniería Mecánica y Fabricación, Universidad de Sevilla, Seville, Spain; ^2^ School of Mechanical, Medical and Process Engineering, Queensland University of Technology, Brisbane, QLD, Australia

**Keywords:** alendronate, bone remodeling, bone cell population model, postmenopausal osteoporosis, pharmacokinetics, pharmacodynamics

## Abstract

Alendronate is the most widely used drug for postmenopausal osteoporosis (PMO). It inhibits bone resorption, affecting osteoclasts. Pharmacokinetics (PK) and pharmacodynamics (PD) of alendronate have been widely studied, but few mathematical models exist to simulate its effect. In this work, we have developed a PK model for alendronate, valid for short- and long-term treatments, and a mechanistic PK-PD model for the treatment of PMO to predict bone density gain (BDG) at the hip and lumbar spine. According to our results, at least three compartments are required in the PK model to predict the effect of alendronate in both the short and long terms. Clinical data of a 2-year treatment of alendronate, reproduced by our PK-PD model, demonstrate that bone response is site specific (hip: 7% BDG, lumbar spine: 4% BDG). We identified that this BDG is mainly due to an increase in tissue mineralization and a decrease in porosity. The difference in BDG between sites is linked to the different loading and dependence of the released alendronate on the bone-specific surface and porosity. Osteoclast population diminishes quickly within the first month of alendronate treatment. Osteoblast population lags behind but also falls due to coupling of resorption and formation. Two dosing regimens were studied (70 mg weekly and 10 mg daily), and both showed very similar BDG evolution, indicating that alendronate accumulates quickly in bone and saturates. The proposed PK-PD model could provide a valuable tool to analyze the effect of alendronate and to design patient-specific treatments, including drug combinations.

## 1 Introduction

Osteoporosis is a disease caused by an imbalance in the remodeling process. Resorption of the old bone, carried out by osteoclasts, predominates over osteoid formation by osteoblasts. This leads to net bone loss and a decrease in the stiffness and strength of bones. Around 9 million osteoporotic fractures occurred worldwide in 2000, with the highest incidence in Europe (Johnell and Kanis, 2006). Over 27.5 million people suffer from this disease in Europe and nearly 3.5 million fractures occur every year, raising healthcare costs to approximately 37 billion euros (Hernlund et al., 2013). Postmenopausal osteoporosis (PMO), resulting from estrogen deficiency, is the most common type of osteoporosis (Eastell et al., 2016).

At present, the most widely used drug for the treatment of PMO is alendronate (Cummings et al., 2020), a bisphosphonate usually administered orally once a week in 70 mg tablets, though other treatment regimens are also prescribed. Alendronate inhibits bone resorption by osteoclasts *via* two different mechanisms: a reduction in osteoclasts' resorbing capacity and inducing their apoptosis (Halasy-Nagy et al., 2001; Yu et al., 2018; Takagi et al., 2021).

Pharmacokinetics (PK) models intend to explain the absorption, distribution, and elimination of a drug in the body. These PK models constitute the first step to developing pharmacodynamics (PD) models, in which the effect of the drug on the body is analyzed. The combination of both is usually termed as PK-PD models.

Pharmacokinetics of alendronate has been widely studied. Many experimental works have been carried out both on animals and humans. Regarding the latter, there is considerable clinical information about the evolution of the alendronate concentration in plasma (Cocquyt et al., 1999; Chae et al., 2014) and urine excretion (Cocquyt et al., 1999; Kang et al., 2006; Acotto et al., 2012; Chae et al., 2014) during the first hours after alendronate intake. However, to the best of our knowledge, no clinical data have been published regarding the long-term plasma concentration, and only one work has reported on experimental urine excretion in long term (Khan et al., 1997). Despite the available experimental data, only a few mathematical models have been developed to simulate the pharmacokinetics of alendronate or other bisphosphonates (Cremers et al., 2002; Pillai et al., 2004; Sedghizadeh et al., 2013; Chae et al., 2014). These are compartmental models where each compartment represents a part of the body, or rather, a distribution volume where the drug is delivered. These PK models aim at reproducing the temporal evolution of the drug in each compartment. Most of these PK models simply fitted the experimental data from short-term experiments (Pillai et al., 2004; Sedghizadeh et al., 2013; Chae et al., 2014), making their use unreliable for osteoporosis treatments, which normally last for years. Cremers et al. (2002) developed a PK model of pamindronate by fitting the short- and long-term clinical results, but actually they had to use long-term urine excretion data of alendronate (Khan et al., 1997) because the results for pamindronate were not available. To the best of the authors' knowledge, in the case of alendronate treatment, no model could be found in the literature that fits both short- and long-term plasma concentration and/or urine excretion.

Regarding pharmacodynamics, the effects of alendronate on bone density gain (BDG), reduction of fracture risk, and bone turnover markers are well-documented in the literature. However, few mathematical models have been developed to simulate the pharmacodynamics of bisphosphonates and, in particular, alendronate. A large number of these models describe the pharmacodynamics of alendronate in a simplistic way in order to predict the evolution of (systemic) bone turnover markers (Hernández et al., 2002; Pillai et al., 2004). However, they do not consider bone remodeling and its underlying mechanobiological processes. Therefore, these models are unable to predict site-specific bone mineral density (BMD) changes during drug treatment. Recently, Ashrafi et al. (2021) applied a PK-PD model implemented in a bone remodeling model to simulate the behavior of dental implants in patients undergoing bisphosphonate treatment. However, they used ibandronate and implemented the PK model proposed by Pillai et al. (2004), who only used short-term clinical data to adjust their model, which probably makes its use for simulating long-term treatment questionable.

Based on the previous review, the objectives of this work are the following:

• To develop a comprehensive PK model for alendronate valid for short- and long-term treatment.
• To develop a mechanistic PK-PD model of alendronate treatment of PMO to predict site-specific changes in BMD at the hip and lumbar spine.

This article is organized as follows: in Section 2, we provide a detailed description of the different PK and PD models tested in this work and the methods used to fit their constants. The comparison of clinical data with the results provided by the models is reported in Section 3, together with the values of the fitted constants. The results are discussed in detail with respect to the alendronate literature in Section 4.

## 2 Materials and methods

In order to develop a PK-PD model, it is important to note that the PK and the PD model influence one another, that is, they are not independent. Herein, we first develop the PK model for alendronate with the aim of identifying the most suitable compartment interactions for both short- and long-term responses.^1^ At the second stage, we added the PD model which is based on a BCPM of bone remodeling in order to assess changes in BMD at different bone sites. For the case of alendronate treatment of PMO, the addition of the PD will have a greater influence in long term, as the effect of alendronate and its return rate to plasma depends on osteoclasts' action and is more noticeable in long term (months, years) than in short term (hours).

### 2.1 Pharmacokinetics

The aim of this section was to develop a model to reproduce the pharmacokinetics of alendronate while temporarily neglecting the pharmacodynamics of the drug. Compartmental models will be used to fulfill this objective.

Alendronate is distributed throughout the body *via* the blood plasma. It directly reaches the plasma in the case of intravenous (IV) administration or through the gastrointestinal tract when delivered orally. It does not appear to be metabolized in humans, and it is cleared from the plasma by deposition onto bone and elimination via renal excretion (Porras et al., 1999). Once in the plasma, alendronate is quickly distributed into the non-calcified tissues of the body, followed by redistribution in bone or renal elimination (Lin et al., 1991; Porras et al., 1999).

**FIGURE 1:**
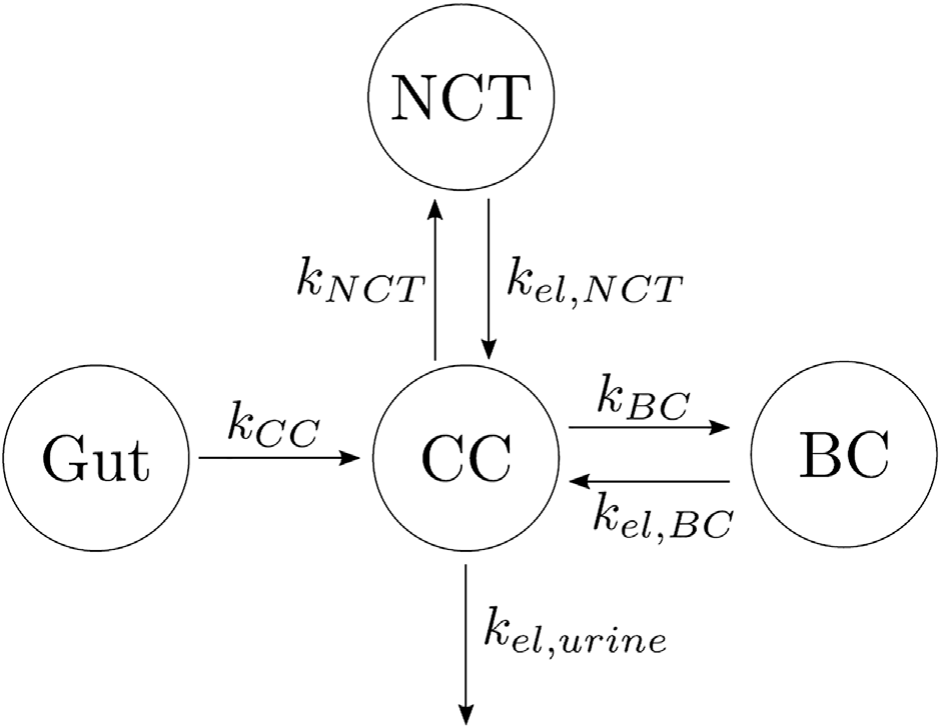
Alendronate three-compartment PK model “in parallel.”

Based on that knowledge, a three-compartment model was developed as depicted in Figure 1, where CC stands for the central compartment (plasma), NCT for the non-calcified tissues, and BC for the bone compartment, with the gut compartment added in the case of oral doses. The arrows indicate the flow of alendronate, *k*_*i*_ is the absorption rate constant, and *k*_*el*,*i*_ is the elimination rate constant, with i = CC, NCT, BC, or urine. The different compartments where alendronate is distributed and the interconnections between them are represented in Figure 1, with the renal excretion represented by an elimination term from the CC (urine excretion). In the following, this model is denoted as a three-compartment model “in parallel” to distinguish it from another model presented later with three compartments and two of them connected “in series.”

The differential equations governing the temporal evolution of alendronate in the three-compartment PK model “in parallel” are:

$$\begin{array}{l} \begin{array}{cc} & \frac{dAle_{CC}}{dt}=F\cdot k_{CC}Ale_{\mathrm{Gut}}+k_{el,BC}Ale_{BC}+k_{el,NCT}Ale_{\mathrm{NCT}}\\ & \;-\left(k_{BC}+k_{\mathrm{NCT}}+k_{el,urine}\right)Ale_{CC} \end{array}, \end{array}$$ (1)

$$\frac{dAle_{BC}}{dt}=k_{BC}Ale_{CC}-k_{el,BC}Ale_{BC},$$ (2)

$$\frac{dAle_{\mathrm{NCT}}}{dt}=k_{\mathrm{NCT}}Ale_{CC}-k_{el,NCT}Ale_{\mathrm{NCT}},$$ (3)

$$\frac{dAle_{\mathrm{Gut}}}{dt}=-k_{CC}Ale_{\mathrm{Gut}},$$ (4)

$$\frac{dAle_{\mathrm{Urine}}}{dt}=k_{el,urine}Ale_{CC},$$ (5)

where *Ale*_*CC*_, *Ale*_*BC*_, *Ale*_*NCT*_, *Ale*_*Gut,*_ and *Ale*_*Urine*_ are the amount of alendronate in the central compartment, bone compartment, non-calcified tissues, gut, and urine, respectively. *F* is the bioavailability. If the dose is administered intravenously, the gut compartment will not appear; therefore, the term *F* ⋅ *k*_*CC*_ *Ale*_*Gut*_ in Eq. 1 must be replaced by the IV dose rate, while Eq. 4 is no longer needed. The concentration in the central compartment was calculated as follows:

$$\left[Ale_{CC}\right]=\frac{Ale_{CC}}{V_{c}},$$ (6)

where *V*_*c*_ is the volume of distribution of the central compartment.

**FIGURE 2:**
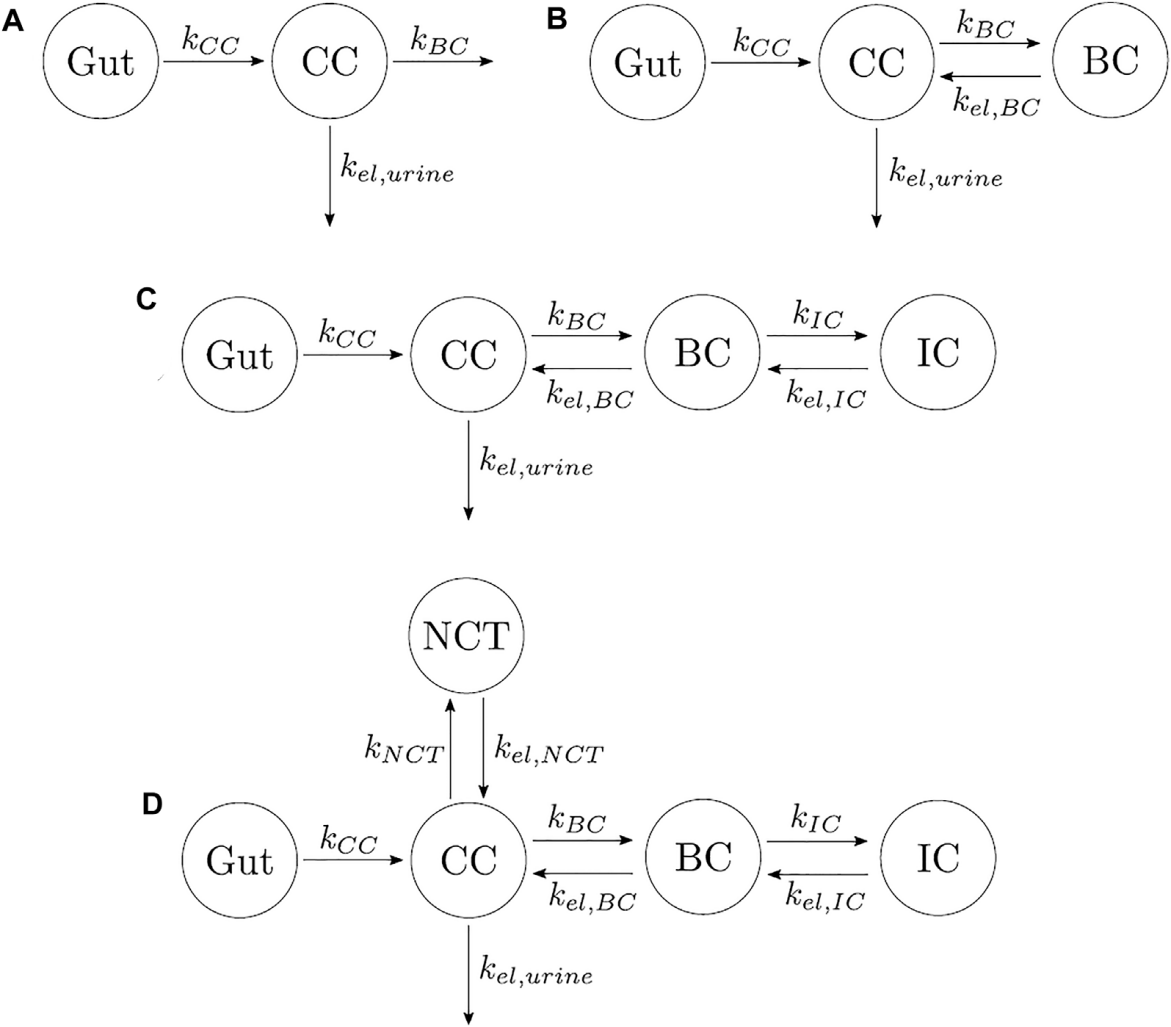
Different types of PK models for alendronate considering various numbers of compartments and different compartmental interactions. **(A)** One-compartment model with two elimination mechanisms. **(B)** Two-compartment model. **(C)** Three-compartment model “in series.” **(D)** Four-compartment model.

Other authors have proposed models with different numbers of compartments and different compartmental interactions. In order to find the most suitable PK model which minimizes the error between experimental data and model results, we investigated four additional models described below:

(1) One-compartment model with two elimination mechanisms, proposed by Chae et al. (2014) (see Figure 2A). This model has an important drawback. It is known from the literature that alendronate is only excreted by urine (Porras et al., 1999), but the model consists of a CC and two exits: one to urine and another to a non-specified organ (in the original model, the outflow rates were termed *k*_*urine*_ and *k*_*non−urine*_). The latter was renamed here as *k*_*BC*_ in order to have an analogous terminology in all models. To overcome this drawback, the following two-compartment model was proposed.
(2) Two-compartment model. The only difference with the one-compartment model is that one of the exits from the CC flows into the BC, and there is a return flow from BC to CC (see Figure 2B).
(3) Three-compartment model “in series.” This model adds to the previous model, a compartment connected to the BC, as seen in Figure 2C. The model was called “in series” to distinguish it from the three-compartment model “in parallel.” This model considers the different availability of the alendronate deposited in bone. To this end, it distinguishes the drug that has been deposited near the bone matrix–marrow interface from the alendronate that was buried deeper into the bone matrix. The former, termed active alendronate (and contained in the BC), is more accessible for osteoclasts to resorb it since resorption occurs mainly on the bone matrix–marrow interface. Thus, it can affect the osteoclastic activity through endocytosis. On the contrary, the latter is assumed to be inaccessible to resorption and was termed inactive alendronate (contained in the inactive compartment, IC) (Porras et al., 1999). Cremers et al. (2002) used a similar model for pamindronate.
(4) Four-compartment model. It was proposed by Porras et al. (1999), although they did not test it. It can be considered the combination of the three-compartment models “in parallel” and “in series,” where the biological backgrounds of both apply (see Figure 2D).

The differential equations governing the previous models are not included in the main text but can be consulted in the Supplementary Material. In accordance with the literature (Lin et al., 1992; Cocquyt et al., 1999), no saturation has been considered for the bone compartment in any of the PK models, though the concept of saturation will be introduced later when the fully mechanistic PK-PD model is developed.

The information found in the literature about the pharmacokinetics of alendronate consists of clinical data of the serum concentration in short term and urine excretion amount in short and long terms. Little is known about urinary excretion in long term, and only the experimental data obtained by Khan et al. (1997) could be found to validate these specific results. The experimental data from Chae et al. (2014) were used to adjust the serum concentration in short term, while those obtained by Kang et al. (2006) were used for the short-term urinary excretion. Other authors measured similar values for both types of results (Cocquyt et al., 1999; Acotto et al., 2012). It is important to note that the results from Chae et al. and Kang et al. correspond to a single oral administration (70 mg of alendronate) to healthy volunteers, while the results from Khan et al. correspond to IV administration (7.5 mg of alendronate infused over 12 h every day for 4 days, being a total dose of 30 mg) to postmenopausal women. These dosing regimens were simulated with the PK models, and their constants were fitted to reproduce the clinical results (short-term serum concentration and short-term and long-term urinary excretion). The goodness of fit of the three curves was jointly evaluated to identify the most suitable PK model. A genetic algorithm was used to minimize the difference between the curves generated by the model and the clinical results. Since the three sets of clinical results were given in different units, the root mean squared error of each curve was normalized with the mean of each experimental curve in order to express the errors as a coefficient of variation so that they could be summed up. Finally, this sum was divided by 3 and expressed in percentage to yield *E*_*PK*_. This error measure represents the average error over all three experiments and was the objective function minimized with the genetic algorithm:

$$\begin{array}{cc} E_{PK}= & \left(\frac{\sqrt{\frac{\overset{N_{\mathrm{Chae}}}{\underset{i=1}{\sum }}{\left(S_{\mathrm{model}}\left(t_{i}\right)-S_{\mathrm{Chae}}\left(t_{i}\right)\right)}^{2}}{N_{\mathrm{Chae}}}}}{\mu_{\mathrm{Chae}}}+\frac{\sqrt{\frac{\overset{N_{\mathrm{Kang}}}{\underset{i=1}{\sum }}{\left(sU_{\mathrm{model}}\left(t_{i}\right)-sU_{\mathrm{Kang}}\left(t_{i}\right)\right)}^{2}}{N_{\mathrm{Kang}}}}}{\mu_{\mathrm{Kang}}}+\right.\\ & +\left.\frac{\sqrt{\frac{\overset{N_{\mathrm{Khan}}}{\underset{i=1}{\sum }}{\left(lU_{\mathrm{model}}\left(t_{i}\right)-lU_{\mathrm{Khan}}\left(t_{i}\right)\right)}^{2}}{N_{\mathrm{Khan}}}}}{\mu_{\mathrm{Khan}}}\right)\cdot \frac{100}{3} \end{array}$$ (7)

where *N*_*j*_ is the number of time points and *μ*_*j*_ is the mean of experimental values over the respective time points for each set of clinical data (*j* = *Chae*, *Kang*, *Khan*). The variables *S*_*j*_(*t*_*i*_), *sU*_*j*_(*t*_*i*_), and *lU*_*j*_(*t*_*i*_) represent the serum concentration, the short-term urinary excretion, and long-term urinary excretion at the time point *t*_*i*_ of the clinical result *j*, respectively. Finally, the subscript *j* = *model* stands for the prediction of the PK model.

### 2.2 Pharmacodynamics

Once a suitable PK model is identified, the effect of alendronate on bone turnover was modeled. To address this aim, a previously published mathematical BCPM describing the bone cell interactions was used (Martin et al., 2019). This model considers catabolic (RANK–RANKL–OPG) and anabolic (Wnt–Scl– LRP5/6) signaling pathways, together with the action of parathyroid hormone (PTH), nitric oxide (NO), transforming growth factor beta (TGF-*β*), and mechanobiological feedback on bone cells. The effect of bone mineralization was added following Martínez-Reina and Pivonka (2019) and Martínez-Reina et al. (2008). The accumulation and repair of microstructural damage was also taken into account as in Martínez-Reina et al. (2021).

**FIGURE 3:**
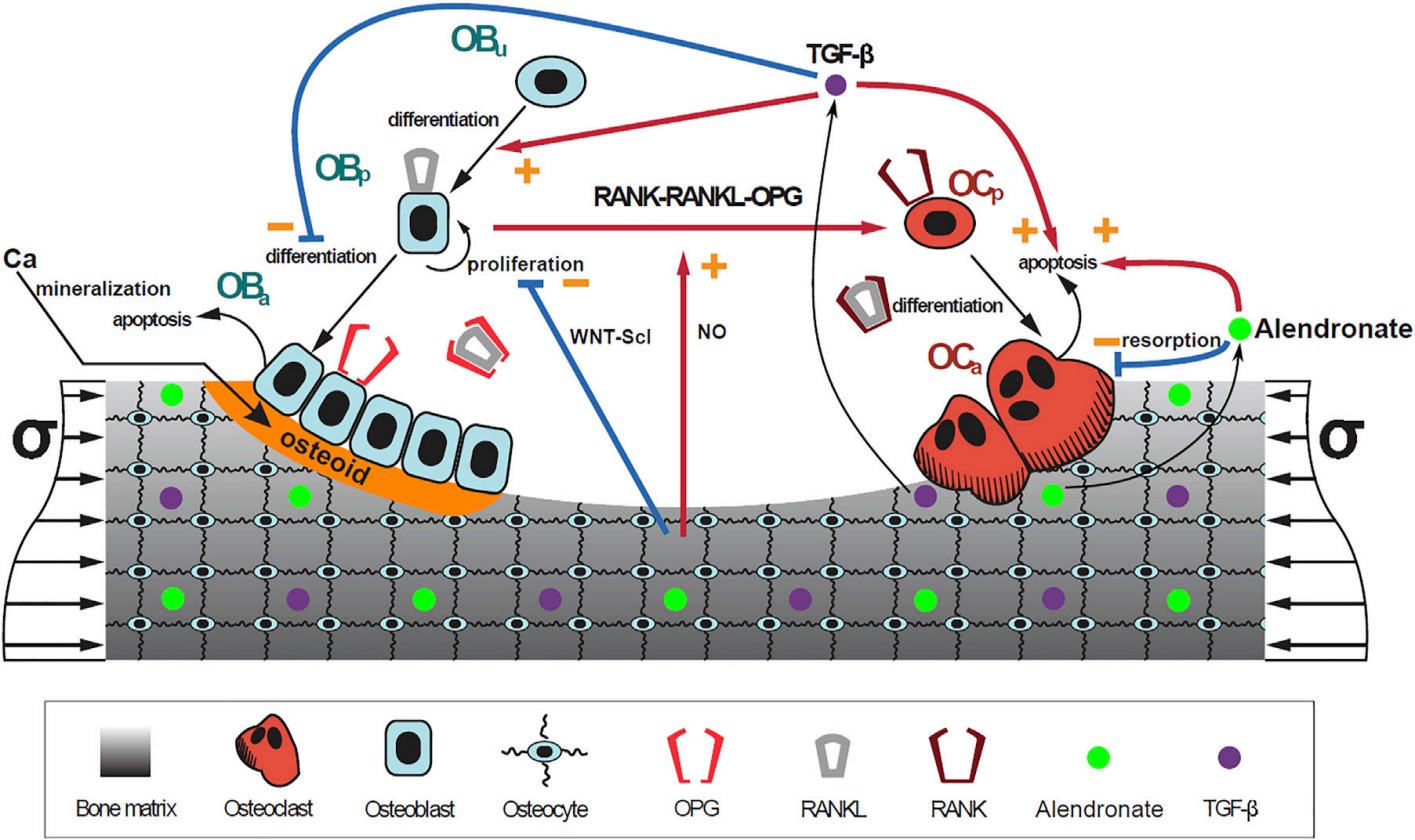
Schematic representation of the mechanistic PK-PD model: bone cell differentiation stages along with biochemical and biomechanical interactions are presented. The mineralization of osteoid is shown in orange. Alendronate doses lead to the distribution of the drug into the bone compartment where it interacts with the osteoclasts.

The bone cell types, whose concentrations are the state variables of the model, are osteoblast precursor cells (Ob_p_), active osteoblasts (Ob_a_), osteoclast precursor cells (Oc_p_), active osteoclasts (Oc_a_), and osteocytes (Ot). The cell pools of uncommitted osteoblasts (Ob_u_) and osteoclasts (Oc_u_) are assumed constant as in Martin et al. (2019).

$$\begin{array}{l} \begin{array}{cc} & \frac{d\;{Ob}_{\text{p}}}{dt}=D_{{Ob}_{\text{u}}}\cdot {Ob}_{\text{u}}\cdot \pi_{\text{act,Obu}}^{\text{TGF}-\beta }-D_{{Ob}_{\text{p}}}\cdot {Ob}_{\text{p}}\cdot \pi_{\text{rep},{Ob}_{\text{p}}}^{\text{TGF}-\beta }\\ & \;+P_{{Ob}_{\text{p}}}\cdot {Ob}_{\text{p}}\cdot \pi_{\text{act},{Ob}_{\text{p}}}^{\text{Wnt}} \end{array}, \end{array}$$ (8)

$$\frac{d\;{Ob}_{\text{a}}}{dt}=D_{{Ob}_{\text{p}}}\cdot {Ob}_{\text{p}}\cdot \pi_{\text{rep},{Ob}_{\text{p}}}^{\text{TGF}-\beta }-\Delta_{{Ob}_{\text{a}}}\cdot {Ob}_{\text{a}},$$ (9)

$$\frac{d\;{Oc}_{\text{p}}}{dt}=D_{{Oc}_{\text{u}}}\cdot {Oc}_{\text{u}}\cdot \pi_{\text{act},{Oc}_{\text{u}}}^{\text{RANKL}}-D_{{Oc}_{\text{p}}}\cdot {Oc}_{\text{p}}\cdot \pi_{\text{act},{Oc}_{\text{p}}}^{\text{RANKL}},$$ (10)

$$\frac{d\;{Oc}_{\text{a}}}{dt}=D_{{Oc}_{\text{p}}}\cdot {Oc}_{\text{p}}\cdot \pi_{\text{act},{Oc}_{\text{p}}}^{\text{RANKL}}-A_{{Oc}_{\text{a}}}\cdot {Oc}_{\text{a}}\cdot \pi_{\text{act},{Oc}_{\text{p}}}^{\text{TGF}-\beta },$$ (11)

$$\frac{d\;Ot}{dt}=\eta \;\frac{d\;f_{bm}}{dt},$$ (12)

where $D_{{Ob}_{\text{u}}}$ , $D_{{Ob}_{\text{p}}}$ , $D_{{Oc}_{\text{u}}}$, and $D_{{Oc}_{\text{p}}}$ are the differentiation rates of Ob_u_, Ob_p_, Oc_u_, and Oc_p_, respectively; $A_{{Oc}_{\text{a}}}$ is the apoptosis rate of Oc_a_, and $\Delta_{{Ob}_{\text{a}}}$ is the rate of clearance of active osteoblasts through apoptosis or differentiation into osteocytes. The variables $\pi_{\text{act,Obu}}^{\text{TGF}-\beta }$ , $\pi_{\text{rep},{Ob}_{\text{p}}}^{\text{TGF}-\beta }$, and $\pi_{\text{act},{Oc}_{\text{p}}}^{\text{TGF}-\beta }$ represent activator and repressor functions related to the binding of TGF-β to its receptor. Similarly, $\pi_{\text{act},{Oc}_{\text{u}}}^{\text{RANKL}}$ and $\pi_{\text{act},{Oc}_{\text{p}}}^{\text{RANKL}}$ are the activator functions related to the RANK–RANKL binding. Finally, $P_{{Ob}_{\text{p}}}$ is the proliferation rate of Ob_p_, a process which is mediated by the Wnt signaling pathway through the activator function $\pi_{act,Ob_{p}}^{\mathrm{Wnt}}$ . These functions, as well as the remaining equations needed to complete the model, are not presented here for brevity but are completely described in the Supplementary Material. A schematic figure of the mechanistic PK-PD model is presented in Figure 3. The values of the model constants are given in Supplementary Table S1.

Finally, Eq. 12 establishes that the population of osteocytes varies as the bone matrix fraction *f*_*bm*_, if the density of osteocytes trapped within the bone matrix, *η*, is assumed constant as carried out in Martin et al. (2019). Bone matrix fraction is defined as the volume of the bone matrix, *V*_*b*_, per total volume of the bone sample (i.e., the representative volume element, *V*_*RVE*_), expressed as a percentage:

$$f_{bm}\left(\%\right)=\frac{V_{b}}{V_{\mathrm{RV E}}}\cdot 100.$$ (13)

Its evolution is obtained through the balance between resorbed and formed tissue:

$$\frac{df_{bm}}{dt}=-k_{\mathrm{res}}\cdot {Oc}_{\text{a}}+k_{\mathrm{form}}\cdot {Ob}_{\text{a}},$$ (14)

where *k*_*res*_ and *k*_*form*_ are the rates of bone resorption and osteoid formation, respectively (see Supplementary Table S1).

This BCPM was integrated into the PK model by including it in the bone compartment (BC, see Figure 1). All the absorption rate constants *k*_*i*_ and the elimination rate constants *k*_*el*,*i*_ fitted for the PK model (see Figure 1) were assumed constant, except for *k*_*el*,*BC*_, which is variable and depends on the osteoclast-mediated release of alendronate from the bone matrix. Note that the latter variable depends on bone turnover (Fisher et al., 2000; Yu et al., 2018), whereas in the PK model of Section 2.1, *k*_*el*,*BC*_ was constant. Now, in the PK-PD model, *k*_*el*,*BC*_ is given by:

$$k_{el,BC}=k_{\mathrm{res}}\;Oc_{a}.$$ (15)

We have assumed that it is the concentration of alendronate in the bone compartment, BC, that controls the action of the drug on the bone remodeling process:

$$\left[Ale_{BC}\right]=\frac{Ale_{BC}}{V_{\mathrm{Bone}}},$$ (16)

where *V*_*Bone*_ is the total volume of bone tissue in the body, 2.23 ⋅ 10^−3^ *m* ^3^, adapted from Valentín (2002) for a 60 kg adult female. The amount of alendronate accumulated within the bone increases with each weekly dose; therefore, if the effect of the drug was proportional to that amount, such an effect would be increasingly pronounced, but this is not observed in the clinical trials (Rizzoli et al., 2002), where the increase of BMD is high during the first months after the beginning of the treatment and slows down in the subsequent months, both in the lumbar spine and the hip. This behavior could be explained by several facts highlighted in the literature:

• Alendronate is preferentially deposited in areas undergoing active resorption (Porras et al., 1999).
• 80% of normal bone turnover occurs in trabecular bone and 20% in cortical bone (Fleisch, 2000).
• A considerably higher amount of alendronate is deposited in trabecular bone than in cortical bone (Lin et al., 1991; Khan et al., 1997).
• The volume of cortical bone is much higher than that of trabecular bone (Fleisch, 2000; Valentín, 2002).

Therefore, a higher proportion of alendronate would be initially deposited in trabecular bone, which explains the fast increase of BMD in the lumbar spine and the hip observed in the clinical results. However, due to the fact that the volume of trabecular bone is much lower than that of cortical bone, in a long treatment, it is expected that the proportion of alendronate that reaches the former will be reduced over time in favor of the latter due to the saturation of the tissue. This would explain the long-term stabilization of bone mass gain observed clinically.

**FIGURE 4:**
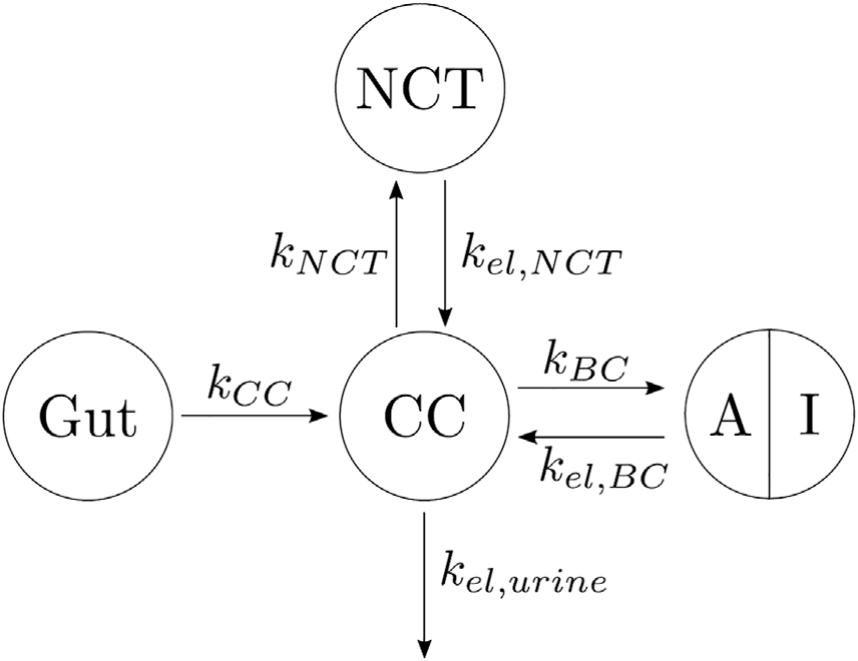
Three-compartment model “in parallel”, adapted with the division of the BC compartment into an active (A) and an inactive (I) subcompartment.

In view of the clinical results, we hypothesized that the BC can be divided into two parts: one part termed active, which is the closest to the bone marrow interface and where the retrieval of alendronate is immediate through bone resorption; the second part termed inactive corresponds to the innermost regions of bone, where alendronate is buried and, to some extent, inaccessible to bone resorption (Sato et al., 1991; Porras et al., 1999). An updated compartmental model is shown in Figure 4. The active subcompartment would predominate in trabecular bone, where most of the tissue is superficial, and its proportion would decrease with porosity as the tissue becomes more compact.

We need a variable to measure this compacity, that is, the proportion of superficial tissue. The specific surface, *S*_*v*_, is defined as the area of bone matrix–marrow interface, *S*_*i*_, per total volume of the bone sample, *V*_*T*_. The following correlation between *S*_*v*_ and vascular porosity, *f*_*vas*_, was given by Martin (1984) and used by Pivonka et al. (2013):

$$S_{v}=\frac{S_{i}}{V_{T}}=32.3\cdot f_{\mathrm{vas}}-93.9\cdot f_{\mathrm{vas}}^{2}+134\cdot f_{\mathrm{vas}}^{3}-101\cdot f_{\mathrm{vas}}^{4}+28.8\cdot f_{\mathrm{vas}}^{5},$$ (17)

where the vascular porosity is $f_{\mathrm{vas}}=1-\frac{f_{bm}}{100}$ and *S*_*v*_ is expressed in *mm* ^2^/*mm* ^3^, being this variable equal to 0 both for *f*_*vas*_ = 0 and *f*_*vas*_ = 1 when no free surface exists. In order to measure the compacity of the tissue, we would need to express that specific surface per bone matrix volume instead of per total volume:

$$\frac{S_{i}}{V_{b}}=\frac{\frac{S_{i}}{V_{T}}}{\frac{V_{b}}{V_{T}}}=\frac{S_{v}}{f_{bm}}.$$ (18)

**FIGURE 5:**
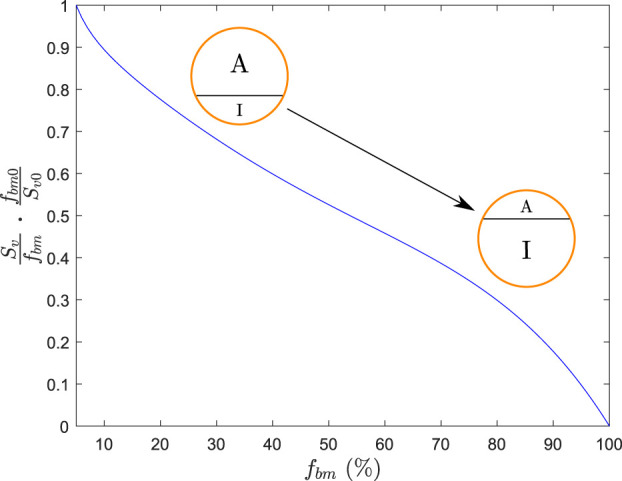
Function that regulates the division between the active (A) and inactive (I) parts of BC.

This quotient is not defined for *f*_*bm*_ = 0, but Eq. 18 is not needed for very low values of *f*_*bm*_ as we can assume that all the tissue is superficial and accessible to bone resorption in a very porous bone. More precisely, we have established *f*_*bm0*_ = 5% as the minimum bone matrix below which all the tissue is considered superficial. We have normalized the quotient in Eq. 18 and used the following expression for the active alendronate, that is, the amount of alendronate contained in the active subcompartment:

$$Ale_{BC,act}=Ale_{BC}\cdot \frac{S_{v}}{f_{bm}}\cdot \frac{f_{\mathrm{bm0}}}{S_{v0}},$$ (19)

where *S*_*v*0_ is the specific area corresponding to the reference value *f*_*bm0*_. The concentration of active alendronate can be assessed as in Eq. 16, that is, $\left[Ale_{BC,act}\right]=\frac{Ale_{BC,act}}{V_{\mathrm{bone}}}$ . The function $\frac{S_{v}}{f_{bm}}\cdot \frac{f_{\mathrm{bm0}}}{S_{v0}}$ that controls the division between the active and inactive parts of the BC is plotted against *f*_*bm*_ in Figure 5. If the tissue has no pores (*f*_*bm*_ = 100%), all the BC is inactive as there is no surface where osteoclasts can resorb bone; thus, there is no release of alendronate from the bone matrix. As porosity increases (*f*_*bm*_ decreases), the ratio between the free surface and bone volume rises, as does the exposure of the drug (the active subcompartment becomes predominant). In contrast, if more tissue is formed than resorbed, *f*_*bm*_ increases and the inactive part becomes predominant as the alendronate that was on the surface is buried into the bone matrix and is thus less accessible to osteoclasts.

So, Eqs 1 and 2 are rewritten as follows by changing the second terms on the right-hand side of both:

$$\begin{array}{l} \begin{array}{cc} & \frac{dAle_{CC}}{dt}=F\cdot k_{CC}Ale_{\mathrm{Gut}}+k_{el,BC}\left[Ale_{BC,act}\right]\;\frac{V_{\mathrm{Bone}}}{\frac{f_{bm}^{\mathrm{aver}}}{100}}+k_{el,NCT}Ale_{\mathrm{NCT}}\\ & \;-\left(k_{BC}+k_{\mathrm{NCT}}+k_{el,urine}\right)Ale_{CC} \end{array}, \end{array}$$ (20)

$$\frac{dAle_{BC}}{dt}=k_{BC}Ale_{CC}-k_{el,BC}\left[Ale_{BC,act}\right]\;\frac{V_{\mathrm{Bone}}}{\frac{f_{bm}^{\mathrm{aver}}}{100}}.$$ (21)

Since [*Ale*_*BC*,*act*_] is the local concentration, *k*_*el*,*BC*_[*Ale*_*BC*,*act*_] represents the local amount of alendronate per unit volume or the RVE, *V*_*RVE*_, released from the bone matrix through resorption. As the flux from BC to CC is systemic, one must consider the contribution of all the *V*_*RVE*_ in the skeleton, which is done in a simplified way through the factor $V_{\mathrm{Bone}}/\left(f_{bm}^{\mathrm{aver}}/100\right)$ . Therefore, $f_{bm}^{\mathrm{aver}}/100$ represents an average bone volume fraction of the skeleton that allows expressing the amount of alendronate per unit volume of bone tissue (recall Eq. 13), which is then multiplied by the total volume of bone tissue in the skeleton, *V*_*Bone*_. Eqs 20 and 21 imply that the systemic flux from BC to CC is proportional to the flux from the local RVE to the CC; if bone turnover changes locally for any reason, it will change accordingly in the whole skeleton. The adopted value $f_{bm}^{\mathrm{aver}}=43.7\%$ was estimated by assuming an average *f*_*bm*_ = 93% for cortical bone (Cardoso et al., 2013) and *f*_*bm*_ = 14% for trabecular one (Ulrich et al., 1999). Therefore, 80% of the bone tissue volume is cortical, and the rest is trabecular (Valentín, 2002).

The active subcompartment is predominant in trabecular bone, whose volume is considerably lower than that of cortical bone. Thus, it is plausible to assume that this subcompartment will become saturated relatively soon, whereas the total alendronate in the BC will not, as indicated in the previous subsection because there is a large volume of cortical bone in the body able to admit high doses of the drug. It could also become saturated in cases of very long treatments, and then the excess of alendronate would be eliminated *via* urine, but the authors found no information on urine excretion in long treatments. The active alendronate has also been saturated as a function of *S*_*v*_/*f*_*bm*_, being its maximum concentration:

$${\left[Ale_{BC,act}\right]}^{max}=f\cdot \frac{S_{v}}{f_{bm}}\cdot \frac{f_{\mathrm{bm0}}}{S_{v0}},$$ (22)

with *f* as a constant to be fitted. Thus, as alendronate enters the BC (through Eq. 21), it is divided between the active and inactive subcompartments following Eq. 19 ^2^ until $\left[Ale_{BC,act}\right]={\left[Ale_{BC,act}\right]}^{\max}$ when the active subcompartment becomes saturated and no more drug is allowed in it.

Alendronate has the following two effects on bone cells. On the one hand, it causes the disappearance of clear zones and ruffled borders, disrupting the cytoskeleton of osteoclasts by inhibiting farnesyl pyrophosphate (FPP) synthase, which leads to these structural changes and loss of function (Halasy-Nagy et al., 2001; Takagi et al., 2021). In other words, it limits the resorbing capacity of osteoclasts, which is measured in the model by *k*_*res*_ (see Eq. 14). Consequently, this parameter is reduced as follows:

$$k_{\mathrm{res}}=k_{\mathrm{res,nom}}\left(1-\Pi_{\mathrm{rep}}^{\mathrm{Ale}}\cdot k_{el,BC}\cdot \left[Ale_{BC,act}\right]\right),$$ (23)

where *k*_*res,nom*_ is the nominal value of *k*_*res*_, $\Pi_{\mathrm{rep}}^{\mathrm{Ale}}$ is a constant quantifying the effect of alendronate on the resorbing capacity of osteoclasts, and *k*_*el*,*BC*_ ⋅ [*Ale*_*BC*,*act*_] is the amount of alendronate per unit volume released from the bone matrix through resorption, that is, the concentration of alendronate that affects the surrounding osteoclasts. If Eq. 15 is replaced in Eq. 23:

$$k_{\mathrm{res}}=k_{\mathrm{res,nom}}\left(1-\Pi_{\mathrm{rep}}^{\mathrm{Ale}}\cdot k_{\mathrm{res}}\cdot Oc_{a}\cdot \left[Ale_{BC,act}\right]\right),$$ (24)

from which *k*_*res*_ can be worked out:

$$k_{\mathrm{res}}=\frac{k_{\mathrm{res,nom}}}{1+\Pi_{\mathrm{rep}}^{\mathrm{Ale}}\cdot k_{\mathrm{res,nom}}\cdot Oc_{a}\cdot \left[Ale_{BC,act}\right]}.$$ (25)

The second effect caused by alendronate is the inhibition of the FPP synthase in the mevalonate pathway and the reduction of protein prenylation, an essential post-translational lipid modification required for the function of numerous proteins, thereby inducing apoptosis in osteoclasts (Sato et al., 1991; Halasy-Nagy et al., 2001; Yu et al., 2018; Takagi et al., 2021). To account for this effect, the apoptosis rate was increased in the model as follows:

$$A_{Oc_{a}}=A_{Oc_{a},nom}\left(1+\Pi_{\mathrm{act}}^{\mathrm{Ale}}\cdot k_{el,BC}\cdot \left[Ale_{BC,act}\right]\right),$$ (26)

where $\Pi_{\mathrm{act}}^{\mathrm{Ale}}$ quantifies the effect of alendronate on the apoptosis of osteoclasts. If Eq. 15 is replaced in Eq. 26:

$$A_{Oc_{a}}=A_{Oc_{a},nom}\left(1+\Pi_{\mathrm{act}}^{\mathrm{Ale}}\cdot k_{\mathrm{res}}Oc_{a}\cdot \left[Ale_{BC,act}\right]\right).$$ (27)

Therefore, the constants to be fitted in the PK-PD model are *k*_*CC*_, *k*_*NCT*_, *k*_*BC*_, *k*_*el*,*NCT*_, *k*_*el*,*urine*_, *V*_*c*_, and *F*, the purely PK constants of the three-compartment model “in parallel”, which was the model eventually selected, and $\Pi_{\mathrm{act}}^{\mathrm{Ale}}$ , $\Pi_{\mathrm{rep}}^{\mathrm{Ale}}$, and *f*, the PD constants that affect the PK model through *k*_*el*,*BC*_ and Eq. 15. It should be noted that due to this coupling through *k*_*el*,*BC*_, the PK model constants needed to be readjusted. The same clinical data used for the fitting of the PK model were used to validate the PK-PD model: Khan et al. (1997) for the urinary excretion in the long term, Chae et al. (2014) for the serum concentration in the short term, and Kang et al. (2006) for the short-term urinary excretion. Furthermore, the effect of alendronate on the bone response, included in the PD model, had to be validated with bone turnover biomarkers. In this case, only local markers could be used, as the present model was applied locally, at a specific bone location that is only characterized by its BMD. For this reason, the bone density gain (BDG), measured with respect to baseline (the beginning of the treatment when bone apparent density was *ρ*_0_), was defined as:

$$BDG\left(\%\right)=\frac{\rho \left(t\right)-\rho_{0}}{\rho_{0}}\cdot 100.$$ (28)

This BDG was compared to clinical data taken from Rizzoli et al. (2002), who measured the BDG in the lumbar spine (for which *f*_*bm*_ = 15% was assumed) and the hip (*f*_*bm*_ = 25%) in postmenopausal women with osteoporosis treated with a once-weekly dose of 70 mg of alendronate during 2 years. PMO was simulated in the *in silico* model as in Martínez-Reina et al. (2022) by introducing a disease-related increase in RANKL production over time, $P_{\mathrm{RANKL}}^{\mathrm{PMO}}$ , through the following sigmoidal function:

$$P_{\mathrm{RANKL}}^{\mathrm{PMO}}=P_{\mathrm{RANKL}}^{\mathrm{PMO,max}}\frac{{\left(t-t_{\mathrm{onset}}\right)}^{\gamma }}{{\left(t-t_{\mathrm{onset}}\right)}^{\gamma }+\delta_{\mathrm{PMO}}^{\gamma }},$$ (29)

where $P_{\mathrm{RANKL}}^{\mathrm{PMO,max}}$ is the maximum (long term) RANKL production rate due to PMO, *t*_*onset*_ is the onset of RANKL increase, *τ*_*PMO*_ is a time constant that establishes when the 50% of $P_{\mathrm{RANKL}}^{\mathrm{PMO,max}}$ is reached, and *δ*_*PMO*_ is a parameter which controls how steep the increase of RANKL is (i.e., the resemblance to a step function). The time between the onset of RANKL increase and the beginning of the treatment was set to 19 years, which is the average time elapsed since menopause in the patients studied by Rizzoli et al. (2002).

The PK-PD model was fitted using a genetic algorithm to minimize the difference between the curves generated by the model and the clinical results. The definition of the error is similar to the one used in the fitting of the PK model (see Eq. 7) but with two differences: 1) the BDG results were also compared, in this case, to those obtained by Rizzoli et al. (2002); 2) it was necessary to distinguish between two types of bone and evaluate them separately (*f*_*bm*_ = 15%, assumed for the lumbar spine, and *f*_*bm*_ = 25% assumed for the hip) as Rizzoli et al. (2002) did in their clinical measurements of BDG. This distinction had to be carried out also for the serum concentration and short- and long-term urinary excretion since the corresponding *in silico* results depend on *f*_*bm*_ (see Eqs 15, 25, and 27). In fact, Eq. 15 couples the PK and PD models and makes the prediction of the serum and urine profiles also dependent on *f*_*bm*_. Thus, the error defined as the objective function and minimized with the genetic algorithm was

$$\begin{array}{cc} & E=\overset{2}{\underset{k=1}{\sum }}\left(\frac{\sqrt{\frac{\overset{N_{\mathrm{Chae}}}{\underset{i=1}{\sum }}{\left(S_{\mathrm{model,k}}\left(t_{i}\right)-S_{\mathrm{Chae}}\left(t_{i}\right)\right)}^{2}}{N_{\mathrm{Chae}}}}}{\mu_{\mathrm{Chae}}}+\frac{\sqrt{\frac{\overset{N_{\mathrm{Kang}}}{\underset{i=1}{\sum }}{\left(sU_{\mathrm{model,k}}\left(t_{i}\right)-sU_{\mathrm{Kang}}\left(t_{i}\right)\right)}^{2}}{N_{\mathrm{Kang}}}}}{\mu_{\mathrm{Kang}}}+\right.\\ & +\left.\frac{\sqrt{\frac{\overset{N_{\mathrm{Khan}}}{\underset{i=1}{\sum }}{\left(lU_{\mathrm{model,k}}\left(t_{i}\right)-lU_{\mathrm{Khan}}\left(t_{i}\right)\right)}^{2}}{N_{\mathrm{Khan}}}}}{\mu_{\mathrm{Khan}}}+\frac{\sqrt{\frac{\overset{N_{\mathrm{Riz,k}}}{\underset{i=1}{\sum }}{\left(BDG_{\mathrm{model,k}}\left(t_{i}\right)-BDG_{\mathrm{Riz,k}}\left(t_{i}\right)\right)}^{2}}{N_{\mathrm{Riz,k}}}}}{\mu_{\mathrm{Riz,k}}}\right)\cdot \frac{100}{8} \end{array}$$ (30)

where *k* = 1, 2 correspond to *f*_*bm*_ = 15% and *f*_*bm*_ = 25%, respectively, *N*_*j*_ is the number of time points, and *μ*_*j*_ is the mean of those points for each set of clinical data (*j* = *Chae*, *Kang*, *Khan*, *Riz*). The variables *S*_*j*_(*t*_*i*_), *sU*_*j*_(*t*_*i*_), *lU*_*j*_(*t*_*i*_), and *BDG*_*j*_(*t*_*i*_) represent the serum concentration, the short- and long-term urinary excretion, and the BDG at the time point *t*_*i*_, respectively. Finally, the subscript *j* = *model* stands for the prediction of the PK-PD model for each type of bone *k*.

A sensitivity analysis was conducted to evaluate the uncertainty of the model predictions. The fitted constants of the PK-PD model were varied by ± 10%, one at a time, keeping the rest constant.

In order to demonstrate the implications of different assumptions made for the development of the PK-PD model and more specifically, in the distribution of alendronate and its effect on bone turnover, six scenarios have been tested with six different models:

(1) *PK-PD1*. This is the proposed model for which alendronate is assumed to downregulate the resorption capacity of osteoclasts (through Eq. 25) and upregulate its apoptosis rate (through Eq. 27) as confirmed by different authors (Halasy-Nagy et al., 2001; Takagi et al., 2021). It is also hypothesized that the alendronate in BC is divided into active and inactive subcompartments and that the active one can be saturated.
(2) *PK-PD2*. In this model, we assume that alendronate only affects the resorption capacity but not the apoptosis rate ( $A_{Oc_{a}}=A_{Oc_{a},nom}$ in Eq. 27).
(3) *PK-PD3*. Herein, we assume that alendronate only affects the apoptosis rate but not the resorption capacity (*k*_*res*_ = *k*_*res,nom*_ in Eq. 25).
(4) *PK-PD4*. In this model, we assume that the active subcompartment does not exhibit saturation. Therefore, Eq. 22 is removed from this model.
(5) *PK-PD5*. Herein, we assume that all the alendronate stored in the BC (and not just the active one) is accessible to osteoclasts and therefore influences the PD model. This is equivalent to disregarding the distinction between active and inactive subcompartments and replacing [*Ale*_*BC*,*act*_] by [*Ale*_*BC*_] in Eqs 25 and 27.
(6) *PK-PD6*. As in the previous model *PK-PD5*, we assume here that all the alendronate ([*Ale*_*BC*_]) influences the PD model but including saturation similar to models *PK-PD1*, *PK-PD2,* and *PK-PD3* for the active subcompartment. As no distinction is made here between active and inactive, when the total alendronate reaches the maximum concentration, no further alendronate can accumulate in the BC. This is in contrast to models *PK-PD1*, *PK-PD2,* and *PK-PD3*, where alendronate can still enter the inactive subcompartment when the active one is saturated.

The constants of the six models were fitted separately by minimizing the error *E* defined in Eq. 30 with a genetic algorithm. The fitting of models *PK-PD2* and *PK-PD3* was made by enforcing $\Pi_{\mathrm{act}}^{\mathrm{Ale}}=0$ and $\Pi_{\mathrm{rep}}^{\mathrm{Ale}}=0$ , respectively.

Finally, once the constants were fitted to show that the *PK-PD1* model was the most accurate, this was used to predict the BDG in postmenopausal women with osteoporosis treated with a daily dose of 10 mg of alendronate, and the results were compared with the clinical data from Rizzoli et al. (2002).

## 3 Results

The results of the fitting used to determine the most suitable PK model are presented first. Subsequently, the results of integrating this PK model with the various PD models are shown.

### 3.1 Pharmacokinetics

**FIGURE 6:**
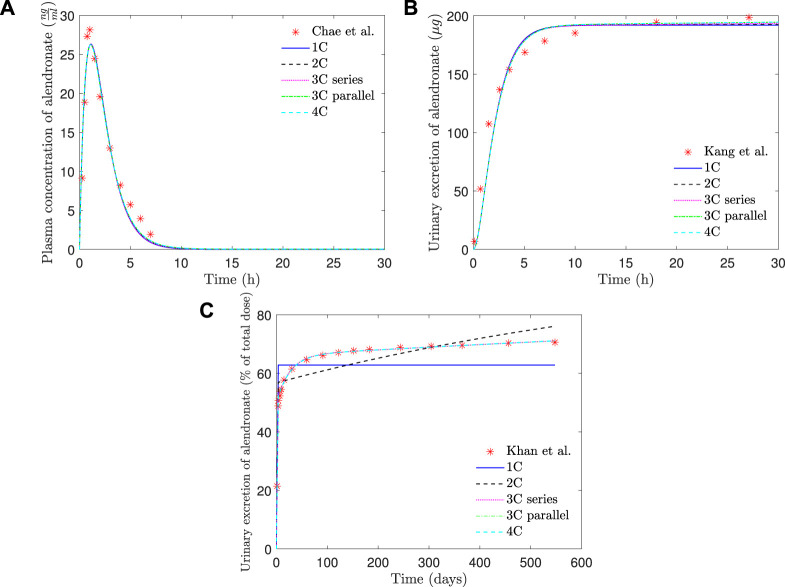
PK model results of alendronate: **(A)** plasma concentration versus time; **(B)** short-term urinary excretion vs. time; and **(C)** long-term urinary excretion vs. time. 1C: one-compartment model with two exits; 2C: two-compartment model; 3C series: three-compartment model “in series”; 3C parallel: three-compartment model “in parallel”; 4C: four-compartment model.

In Table 1, the goodness of fit of each PK model (through error *E*_*PK*_, see Eq. 7) is presented, together with the values of the fitted constants. In Figure 6, the fitting curves of each PK model to the clinical results of plasma concentration and short- and long-term urine excretion are shown.

**TABLE 1:** Summary of the constants and errors of the alendronate PK models. 1C: one-compartment model with two exits; 2C: two-compartment model; 3C series: three-compartment model “in series”; 3C parallel: three-compartment model “in parallel”; 4C: four-compartment model.

	1C	2C	3C “series”	3C “parallel”	4C
*k*_*CC*_ (*day* ^−1^)	19.03	17.26	25.49	16.33	16.21
*k*_*el*,*urine*_ (*day* ^−1^)	15.15	15.34	9.44	14.80	14.90
*k*_*NCT*_ (*day* ^−1^)	—–	—–	—–	6.60	6.67
*k*_*el*,*NCT*_ (*day* ^−1^)	—–	—–	—–	4.95 ⋅ 10^−2^	4.91 ⋅ 10^−2^
*k*_*BC*_ (*day* ^−1^)	9.02	11.64	8.95	7.46	7.52
*k*_*el*,*BC*_ (*day* ^−1^)	—–	1.90 ⋅ 10^−3^	2.40 ⋅ 10^−2^	4.38 ⋅ 10^−4^	4.43 ⋅ 10^−4^
*k*_*IC*_ (*day* ^−1^)	—–	—–	2.60 ⋅ 10^−2^	—–	1.01 ⋅ 10^−5^
*k*_*el*,*IC*_ (*day* ^−1^)	—–	—–	9.36 ⋅ 10^−4^	—–	9.08 ⋅ 10^−3^
*V*_*c*_ (*L*)	3.78	3.73	6.07	3.85	3.82
*F*	4.37 ⋅ 10^−3^	4.84 ⋅ 10^−3^	5.32 ⋅ 10^−3^	5.35 ⋅ 10^−3^	5.36 ⋅ 10^−3^
*E*_*PK*_ (%)	11.777	10.103	8.670	8.553	8.547

As can be seen in Figure 6, all the PK models are able to reproduce with good accuracy the temporal evolution of the concentration of alendronate in the plasma (Figure 6A) and the short-term urinary excretion (Figure 6B), with negligible differences between them. However, the one-compartment model with two exits and the two-compartment model are unable to reproduce urinary excretion in the mid and long terms (see Figure 6C). In the case of the one-compartment model with two exits (blue solid line), the alendronate is eliminated from the central compartment *via* urine, and once it is exhausted, no more can be eliminated, thus leading to a step-like curve. The two-compartment model (black dashed line) shows a significant improvement with respect to the one-compartment model: it yields a curve with a non-zero slope in the mid and long terms, indicating that alendronate continues to be eliminated in those periods. However, that slope does not fit the real one. An option for the fitting procedure was tried in this case: enforcing the long-term slope, but then the model completely overestimated the amount of alendronate excreted in the midterm (30–100 days). The rest of the models (three-compartment “in series” and “in parallel” and four-compartment) produce almost identical results, which are all in excellent agreement with the experimental data.

Table 1 shows that the one-compartment model with two exits and the two-compartment model produced errors considerably higher than the rest of the models, which in turn have similar errors. Therefore, it is important to highlight the two qualitative leaps made when moving from 1 to 2 and from 2 to 3 compartments. In view of these results, it seems that at least three compartments are needed to simulate correctly the alendronate elimination rate over time.

The three-compartment model “in parallel” was selected as the most suitable PK model, because it is simple and able to fit the three experimental curves with good accuracy. The selected model was only slightly outperformed by the four-compartment model, but the difference was virtually negligible, and the latter model is more complex as it depends on two more constants. Moreover, the layout of the three-compartment model “in parallel” is biologically based and allows a direct interpretation of all the compartments and their mutual interaction.

### 3.2 Pharmacodynamics

In Table 2, the final constants fitted for the PK-PD model are presented. If the constants of the PK model (without PD) are compared with those provided in Table 1 for the three-compartment PK model “in parallel,” it can be seen that they are very similar, with slight variations due to coupling with the PD model.

**TABLE 2:** Summary of parameter values for alendronate PK models. Fitted for the complete model (*PK-PD1*), for the model that does not take into account the influence of alendronate on the apoptosis of osteoclasts (*PK-PD2*), for the model that does not consider its influence on the resorbing capacity of osteoclasts (*PK-PD3*), for the model in which the active subcompartment does not exhibit saturation (*PK-PD4*), for the model that ignores the distinction between the active and inactive subcompartments (*PK-PD5*), and for the model that ignores that distinction but implements saturation in the BC (*PK-PD6*). E is the error as defined in Eq. 30.

	*PK-PD1*	*PK-PD2*	*PK-PD3*	*PK-PD4*	*PK-PD5*	*PK-PD6*
*k*_*CC*_ (*day* ^−1^)	16.33	16.33	16.33	28.70	11.28	18.92
*k*_*el*,*urine*_ (*day* ^−1^)	14.80	14.80	14.80	10.61	26.54	10.97
*k*_*NCT*_ (*day* ^−1^)	6.81	6.84	6.66	6.97	19.11	8.40
*k*_*el*,*NCT*_ (*day* ^−1^)	4.90 ⋅ 10^−2^	4.44 ⋅ 10^−2^	4.77 ⋅ 10^−2^	1.34 ⋅ 10^−3^	1.51 ⋅ 10^−3^	2.07 ⋅ 10^−3^
*k*_*BC*_ (*day* ^−1^)	7.39	7.33	7.53	2.61 ⋅ 10^−5^	2.93 ⋅ 10^−4^	8.67
*V*_*c*_ (*L*)	3.84	3.85	3.85	5.33	2.06	5.22
*F*	5.36 ⋅ 10^−3^	5.36 ⋅ 10^−3^	5.36 ⋅ 10^−3^	4.34 ⋅ 10^−3^	4.87 ⋅ 10^−3^	6.41 ⋅ 10^−3^
$\Pi_{\mathrm{act}}^{\mathrm{Ale}}\;\;\left(day/mM\right)$	12.98	——	32.91	16.69 ⋅ 10^5^	34.45 ⋅ 10^4^	11.80
$\Pi_{\mathrm{rep}}^{\mathrm{Ale}}\;\;\left(day/mM\right)$	14.31	20.30	——	27.68 ⋅ 10^4^	48.29 ⋅ 10^3^	3.85
*F*	7.72 ⋅ 10^−2^	7.72 ⋅ 10^−2^	1.07 ⋅ 10^−1^	——	——	1.79 ⋅ 10^−1^
*E* (%)	8.59	8.59	8.59	11.36	11.41	10.69

**FIGURE 7:**
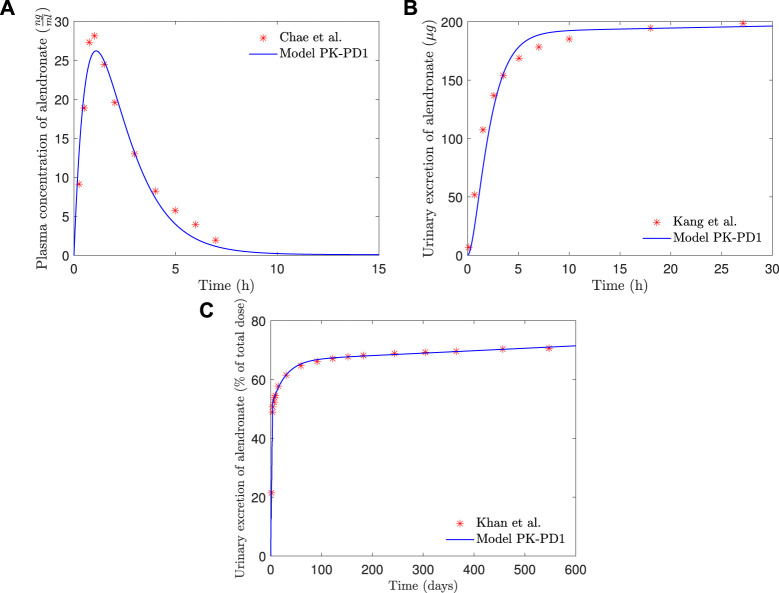
*PK-PD1* model results of alendronate: **(A)** plasma concentration versus time; **(B)** short-term urinary excretion vs. time; and **(C)** long-term urinary excretion vs. time.

In Figure 7, the plasma concentration and the short- and long-term urine excretion curves obtained with the model *PK-PD1* are shown along with the experimental data for *f*_*bm*_ = 15% (results for *f*_*bm*_ = 25% are omitted because the curves virtually overlap with those of *f*_*bm*_ = 15%, and only in the long-term urine excretion, a slight difference can be noted in the final slope). It can be seen that the model *PK-PD1* is able to reproduce, reasonably well, all the experimental curves.

**FIGURE 8:**
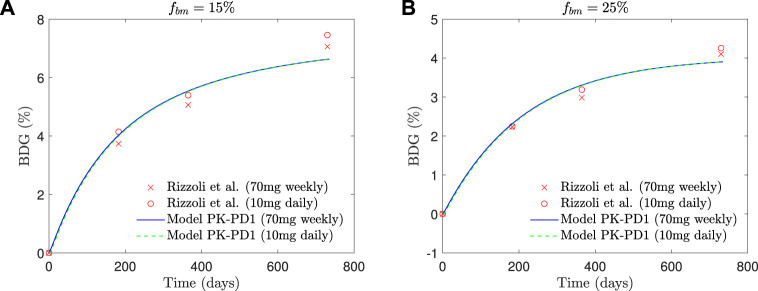
Comparison of BDG vs. time plots of alendronate PK-PD1 model for **(A)** hip, that is, *f*_*bm*_ = 15% and **(B)** lumbar vertebra, that is, *f*_*bm*_ = 25% for a once-weekly 70 mg dose and daily 10 mg dose with the clinical results obtained by Rizzoli et al. (2002).

In Figure 8, the BDG predicted by the model *PK-PD1* is plotted against the experimental results for *f*_*bm*_ = 15% and *f*_*bm*_ = 25%. It can be noted that the model prediction agrees well with the clinical results. In addition to the standard once-weekly 70 mg dose, the BDG for a daily 10 mg dose is also represented, producing both treatments with very similar results.

In Table 3, the results of the sensitivity analysis of the *PK-PD1* model are presented. It can be seen that each constant affects at least one curve, having an important effect on the global error, except in the case where *k*_*el*,*NCT*_ is reduced by 10%, which does not change the fitting. The curves generated by the variations of each constant are included in the Supplementary Material.

**TABLE 3:** Sensitivity analysis of the constants of the *PK-PD1* model. The fitted constants of the PK-PD model were varied by ± 10%, one at a time, keeping the rest constant. $e_{j}^{k}$ is the error in each curve, where *k* = 1, 2 corresponds to *f*_*bm*_ = 15% and *f*_*bm*_ = 25%, respectively, and *j* = *Chae*, *Kang*, *Khan*, *Riz* indicates the curve. These error terms correspond to each of the summations divided by the corresponding mean in Eq. 30. E is the error as defined in Eq. 30.

		$e_{\mathrm{Chae}}^{1}$	$e_{\mathrm{Khan}}^{1}$	$e_{\mathrm{Kang}}^{1}$	$e_{\mathrm{Riz}}^{1}$	$e_{\mathrm{Chae}}^{2}$	$e_{\mathrm{Khan}}^{2}$	$e_{\mathrm{Kang}}^{2}$	$e_{\mathrm{Riz}}^{2}$	*E*(%)
*PK-PD1*		0.131	0.106	0.019	0.090	0.131	0.106	0.019	0.083	8.59
*k*_*CC*_	+10%	0.159	0.097	0.019	0.090	0.159	0.097	0.019	0.083	9.06
	−10%	0.141	0.120	0.019	0.090	0.141	0.120	0.019	0.083	9.15
*k*_*el*,*urine*_	+10%	0.142	0.116	0.044	0.090	0.142	0.116	0.042	0.083	9.68
	−10%	0.134	0.125	0.042	0.090	0.134	0.126	0.044	0.083	9.75
*k*_*NCT*_	+10%	0.135	0.106	0.021	0.090	0.135	0.106	0.021	0.083	8.73
	−10%	0.131	0.112	0.022	0.090	0.131	0.111	0.022	0.083	8.78
*k*_*el*,*NCT*_	+10%	0.131	0.106	0.020	0.090	0.131	0.106	0.020	0.083	8.61
	−10%	0.131	0.106	0.019	0.090	0.131	0.106	0.019	0.083	8.59
*k*_*BC*_	+10%	0.135	0.107	0.033	0.090	0.135	0.107	0.035	0.083	9.08
	−10%	0.131	0.112	0.042	0.090	0.131	0.112	0.039	0.083	9.26
*V*_*c*_	+10%	0.168	0.106	0.019	0.090	0.169	0.106	0.019	0.083	9.52
	−10%	0.186	0.106	0.019	0.090	0.186	0.106	0.019	0.083	9.95
*F*	+10%	0.177	0.153	0.019	0.090	0.177	0.153	0.019	0.083	10.89
	−10%	0.175	0.151	0.019	0.090	0.175	0.151	0.019	0.083	10.81
$\Pi_{\mathrm{act}}^{\mathrm{Ale}}$	+10%	0.131	0.106	0.019	0.104	0.131	0.106	0.019	0.099	8.96
	−10%	0.131	0.106	0.019	0.109	0.131	0.106	0.019	0.092	8.94
$\Pi_{\mathrm{rep}}^{\mathrm{Ale}}$	+10%	0.131	0.106	0.019	0.164	0.131	0.106	0.019	0.149	10.33
	−10%	0.131	0.106	0.019	0.166	0.131	0.106	0.019	0.134	10.17
*F*	+10%	0.131	0.106	0.020	0.218	0.131	0.106	0.019	0.191	11.54
	−10%	0.131	0.106	0.019	0.212	0.131	0.106	0.019	0.172	11.22

If the influence of alendronate on the apoptosis and the resorbing capacity of osteoclasts is removed from the PK-PD model separately (models *PK-PD2* and *PK-PD3*), the fitting errors are similar to those of the complete model (*PK-PD1*) (see Table 2). This indicates that both effects of alendronate have the same consequences, at least in the case simulated here with this model, as will be discussed later on.

**FIGURE 9:**
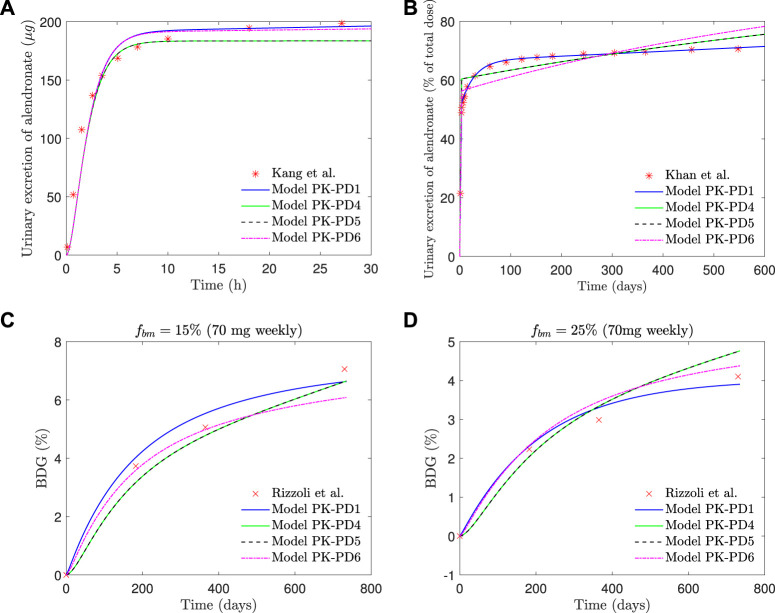
*PK-PD1*, *PK-PD4*, *PK-PD5,* and *PK-PD6* model results. **(A)** Alendronate short-term urinary excretion vs. time; **(B)** alendronate long-term urinary excretion vs. time; **(C)** BDG vs. time for hip, that is, *f*_*bm*_ = 15% and **(D)** BDG vs. time for lumbar vertebra, that is, *f*_*bm*_ = 25% for a once-weekly 70 mg dose.

If the saturation of the active subcompartment of BC is removed (model *PK-PD4*) or if no distinction is made between the active and inactive subcompartments, either with or without saturation (models *PK-PD6* and *PK-PD5*, respectively), the fitting errors are higher (see Table 2) and therefore indicating that both model features, the distinction between subcompartments and the saturation, are important for the bone response. The plasma concentration curves are similar to those obtained with the model *PK-PD1*, but the fitting of the urine curves, particularly the long-term urine excretion, is significantly worse (see Figure 9). Regarding the bone gain, a difficulty is detected in adjusting the response for *f*_*bm*_ = 15% and *f*_*bm*_ = 25% simultaneously. In general, BDG is underestimated by models *PK-PD4*, *PK-PD5*, and *PK-PD6* for *f*_*bm*_ = 15% and overestimated for *f*_*bm*_ = 25%, as can be seen in Figure 9.

**FIGURE 10:**
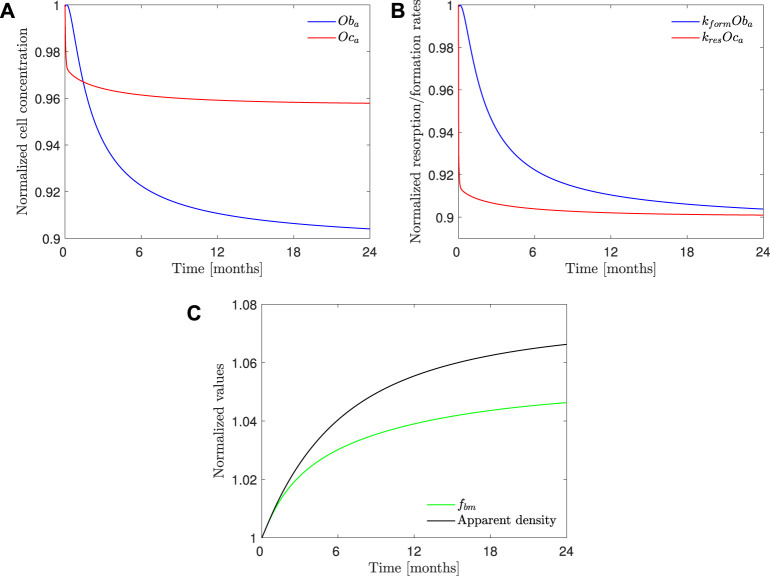
*PK-PD1* model results of 70 mg weekly treatment of alendronate in the case of the hip. Temporal evolution of **(A)** osteoblasts and osteoclasts concentration; **(B)** bone resorption and formation rates; **(C)** apparent density and *f*_*bm*_. All the results are normalized with the values at the beginning of the treatment.

Figure 10 shows the evolution of osteoclasts and osteoblasts population, bone resorption and formation rates, apparent density, and *f*_*bm*_ for model *PK-PD1* in a 70 mg weekly treatment of alendronate and in the case of the hip. The results for the 10 mg daily treatment are very similar, and those of the lumbar spine show the same tendency. Alendronate treatment decreases both cell populations and thus reducing bone turnover rate. It also decreases the resorption capacity of osteoclasts *k*_*res*_, and as a consequence, the formation rate is slightly higher than the resorption rate, thereby reversing the tendency of PMO. During the treatment, *f*_*bm*_ increases, but not enough to account for the BDG as can be deduced from the fact that the increment in apparent density is higher than that of *f*_*bm*_, that is, the BDG is not only due to the reduction in porosity but also because of the mineralization of the tissue, produced by the decrease in bone turnover, similarly to what occurs in other antiresorptive treatments (Martínez-Reina and Pivonka, 2019).

## 4 Discussion

The PK-PD model of alendronate proposed in this work was able to reproduce the short- and long-term pharmacokinetics reported in the literature (see Figure 7), as well as the BDG in a case of PMO for two different treatment protocols (10 mg administered daily and 70 mg administered weekly) for two different bone sites, that is, the lumbar spine and the hip (see Figure 8).

To model the pharmacokinetics of the drug, several models have been tested to conclude that the best option was a three-compartment model “in parallel.” Cremers et al. (2002) also used a three-compartment scheme in their pamindronate PK model (i.e., a different bisphosphonate); although in order to model its long-term treatment, the authors fitted the long-term urine excretion curves of the alendronate data obtained by Khan et al. (1997). These authors used an “in series” layout instead of our “in parallel” model. Differences may exist between the pharmacokinetics of pamindronate and alendronate, but, in any case, the fitting of both types of model (“in series” and “in parallel”) produced similar errors in our analysis, and we finally opted for the “in parallel” layout for two reasons: 1) the fitting error is slightly lower and 2) its biological basis is more solid. As Lin et al. (1991) experimentally demonstrated in animal models, alendronate is absorbed by both bone and non-calcified tissues, with a faster return to plasma from the latter compartment. Pillai et al. (2004) used a four-compartment model for ibandronate, although they stated that a three-compartment model was adequate. They introduced a fourth compartment on the basis to improve model performance. Other authors used models with a lower number of compartments (Sedghizadeh et al., 2013; Chae et al., 2014), but they did not fit the long-term experimental data. In our work, we conclude that one or two compartments are sufficient to predict the short-term behavior of bisphosphonates, but at least three compartments are required to also fit long-term experimental data.

The value obtained for bioavailability (see Table 2), *F* = 0.5%, is in agreement with the values found in the literature (Porras et al., 1999; Drake et al., 2008; Cummings et al., 2020).

The addition of the PD model did not significantly affect the results obtained with the PK model alone (see Figure 7). In the complete PK-PD model (*PK-PD1*), the return flow of alendronate from the BC to the CC is no longer independent of the osteoclast activity, that is, it is controlled by the elimination rate *k*_*res*_ *Oc*_*a*_ and therefore variable. However, the plasma concentration and the short- and long-term urine excretion have a similar goodness of fit compared to the purely PK model. For the first two quantities, the period of interest is significantly shorter than the time required to observe bone cellular changes during remodeling. For the long-term urine excretion, one would anticipate a significant influence. However, the osteoclast population is quickly stabilized and the same occurs with *k*_*res*_ (see Figure 10), therefore producing an approximately constant elimination rate, which is responsible for the urinary excretion rate in the long-term, slow but steady as seen in Figure 7.

The sensitivity analysis (see Table 3) showed that the variation of each constant has a significant effect on at least one of the curves. For the relatively small variation (10%), the PK constants do not have a significant effect on BDG, and the same occurs with the PD constants on the plasma concentration and urine excretion experimental curves. Another conclusion that can be drawn from the sensitivity analysis is the fact that the PK behavior (plasma and urine curves) is similar for *f*_*bm*_ = 15% and *f*_*bm*_ = 25% but not in the case of the PD behavior (BDG).

Our PK-PD model yielded very similar BDGs for the 2-year treatment period for both dose regimes (70 mg weekly and 10 mg daily), reproducing the clinical results obtained by Rizzoli et al. (2002) very closely in the lumbar spine and hip. This similarity between both protocols is also in agreement with the clinical results by Schnitzer et al. (2000). We identified that BDG can be due to two effects. First, to a decrease in bone turnover rate which affects osteoclasts more intensely. Their population falls less than that of osteoblasts (Figure 10C), but since the resorbing capacity (*k*_*res*_) of the existing osteoclasts is also diminished by the drug, the resorption rate drops more acutely (Figure 10B). This fact makes *f*_*bm*_ rise slightly with the treatment. However, this rise cannot completely explain the predicted BDG, which was also influenced by the increased mineralization of the tissue produced by the decreased bone turnover, similarly to what occurs in other antiresorptive treatments (Martínez-Reina and Pivonka, 2019). The difference in BDG between sites is linked to the different loading and a dependence of the active alendronate on the bone-specific surface and *f*_*bm*_. A noticeable decrease in the osteoclast population takes place during the first month of the treatment. Osteoblast population lags behind but also falls due to the coupling of bone cells in the first 6 months (see Figure 10). After this rapid decline, there seems to be a stabilization during the rest of the treatment. This behavior was experimentally reported by Rizzoli et al. (2002). Bone resorption markers (N-terminal telopeptide of type 1 collagen, NTx) and bone formation markers (bone-specific alkaline phosphatase, BSAP) exhibited strong decay in approximately the same times, faster for the NTx, and a subsequent stabilization, that is, however, more evident in the clinical results for the case of BSAP (Rizzoli et al., 2002).

As reported in the literature (Halasy-Nagy et al., 2001; Yu et al., 2018; Takagi et al., 2021), the role of alendronate in bone turnover was simulated in the proposed PK-PD model (*PK-PD1*) with a double mechanism of action: a weakening of the resorptive capacity of osteoclasts due to the disappearance of the clear zones and ruffled borders and an upregulation of their apoptosis. The influence of both mechanisms was evaluated by fitting two alternative models (*PK-PD2* and *PK-PD3*) in which only one of them was considered alternatively. It was concluded that considering just one of those mechanisms (any of them) is enough to simulate the action of alendronate, as those alternative models produced similar errors in the fitting procedure (see Table 2). However, this result does not imply that any of them can be simply eliminated from the model, as it has been shown experimentally that both mechanisms take place. Probably, their consequences are similar to the clinical cases simulated here but might be different when longer periods of time are simulated or in more complex scenarios as discussed in the next paragraphs.

One simplification of BCPMs consists of assuming that bone turnover is continuous in time and space. The differentiation of mature osteoclasts and osteoblasts from their precursors occurs intermittently, and if there are no active osteoclasts at a given time point and bone site, then alendronate could not induce apoptosis at that site. On the other hand, only when the osteoclasts are being differentiated, alendronate could prevent the formation of clear zones and ruffled borders in developing osteoclasts. The two mechanisms are activated at different time scales, given that alendronate rapidly induces apoptosis (Takagi et al., 2021), but the differentiation of osteoclasts is a slower process. This would make them not totally equivalent in intermittent models.

Another limitation of the model is the use of a systemic PK model in conjunction with a local PD model. The clinical data of alendronate serum concentration and urine excretion used to fit the model are systemic. Indeed, the PK model depicted in Figure 4 is systemic itself. This means that the CC represents the serum of the whole body and the BC, the whole skeleton. However, the PD model was applied locally, at a certain RVE, in a specific bone site. Therefore, Eq. 15 and the last term of Eq. 21 imply a strong simplification of the model, which consists of assuming that the flux of alendronate from BC to CC at a given bone site is representative of what occurs at the skeleton on average. This concerns *f*_*bm*_ and *Oc*_*a*_ and implies that both variables are assumed to change globally as they do locally. A comprehensive PK-PD model should take into account the flux from every site of the BC following Eq. 15, with the specific values of *f*_*bm*_ and *Oc*_*a*_ at each point. Such a model would need to simulate the whole skeleton, but it must be disregarded for practical reasons.

If not the entire skeleton, at least one complete bone could be simulated with the proposed model. In these organ models, the concentration of alendronate could be heterogeneous with specific bone sites being saturated with the drug and others with low concentrations. The former would correspond to an active part of the BC, where the flux from CC to BC is hindered by the saturation, but the opposite flux would be favored if a resorption event occurred locally. On the contrary, the latter would correspond to the inactive part of the BC, where the drug is mainly accumulated and not released. Therefore, the distinction between active and inactive subcompartments would come naturally.

It must be noted that the predictions of models *PK-PD4*, in which the active subcompartment does not exhibit saturation, and *PK-PD5*, which ignores the distinction between the active and inactive subcompartments, almost coincide. This was expected, because the difference between both models is that the alendronate effect is proportional either to the active or total alendronate, respectively. The former is *f*_*bm*_ dependent, while the latter is not, but the variation of porosity is limited in the simulations carried out here. Thus, the active alendronate remains as a constant fraction of the total as long as saturation is not considered.

Figure 9 shows that the fit of the short-term urinary excretion and BDG curves, though not excessively bad, is worse than in the model *PK-PD1*, and the prediction of the long-term urinary excretion is rather poor. Therefore, in view of the results of models *PK-PD4* and *PK-PD5*, which do not consider saturation, we can conclude that this feature is necessary. The need for saturation is supported by two further reasons. 1) In the clinical results of BDG (see Figure 8), a steep increase is observed at the beginning to slow down shortly thereafter, that is, the effect of alendronate is stronger during the first months, which reinforces the necessity of saturation. 2) According to Lin et al. (1991), alendronate would only stay in the blood for a very short time: some are quickly excreted *via* urine, and some are stored in the non-calcified tissues and the bone, but the return from the former to the blood is faster. So, we can deduce that alendronate is quickly deposited in the bone and returns to the blood *via* bone resorption, which occurs very slowly. According to this, the amount of alendronate accumulated within bone would increase constantly with each weekly dose, along with its effect on bone turnover which would also rise constantly, unless a saturation limit is reached. Clearly, saturation is needed to explain that the effect of the drug is reduced in the midterm.

Another question is how models *PK-PD4* and *PK-PD5* are able to achieve a reasonable fit for BDG without implementing saturation. The answer is that the mathematical fitting procedure is forced to completely readjust the pharmacokinetics, leading to some biological incoherences. First, as can be seen in Table 2, the BC absorption rate constant, *k*_*BC*_, decreases by 4 or 5 orders of magnitude with respect to the standard model, which means that almost no alendronate enters the BC. Moreover, virtually none is stored because the elimination rate constant, *k*_*el*,*BC*_, is higher. This prevents the accumulation of the drug within the bone matrix, and its effect on bone turnover is only achieved for enormous values of $\Pi_{\mathrm{rep}}^{\mathrm{Ale}}$ and $\Pi_{\mathrm{act}}^{\mathrm{Ale}}$ (see Table 2). Finally, although the fit of the urinary excretion curves is not very good with models *PK-PD4* and *PK-PD5*, it would be even worse if the algorithm had not reduced *k*_*el*,*NCT*_. Such reduction leads to higher disposal of the drug in the long term, though not from the BC but from the NCT, which would contradict the literature as the return to the serum is faster from the non-calcified tissues (Lin et al., 1991). In conclusion, the goodness of fit of these models is poor and worse, and they do not make any biological sense.

Once saturation proves necessary, the division of BC into an active and an inactive part also seems crucial, as is evident from the comparison between models *PK-PD1* and *PK-PD6*. Both implement saturation but just of the active part in *PK-PD1* and of the whole BC in *PK-PD6*. The latter is also unable to fit the clinical results, particularly the mid- and long-term urinary excretion because of the following. Saturation is needed to damp bone mass gain in the midterm, as commented before, but if saturation applies to the whole BC, then no more drug can enter the bone after saturation and must be excreted, thus failing to adjust the mid- and long-term excretion curves. In other words, the algorithm is not able to find a saturation limit that meets both requirements and either fails to predict BDG (if the saturation constraint is too loose) or fails to predict the excretion curve (if the saturation constraint is too tight). The latter is what can be seen in Figure 9 (top right, magenta line). The model is forced to reproduce the clinical results of BDG, but then the BC is saturated too soon and from that point on, all the administered drug is excreted at a constant rate. If only the active part of the BC is saturated (as in *PK-PD1*), BDG response may be damped while alendronate can continue to accumulate, now in the inactive part of the BC. This does not mean that this subcompartment cannot saturate, but that saturation is reached much later and probably only if the treatment is very long.

The PK-PD model of alendronate proposed in this work could provide a valuable tool to analyze the effect of alendronate in a large number of scenarios, particularly in PMO, as well as to design patient-specific treatments, including combinations of different drugs.

## Data Availability

The original contributions presented in the study are included in the article/Supplementary Material; further inquiries can be directed to the corresponding author.

## Footnotes

1 It is important to note that if the PK model is not able to predict the concentration profile of the drug over time, the addition of a PD model is unlikely to improve the results.

2 The rest goes to the inactive subcompartment, that is, *Ale*_*BC*,*inact*_ = *Ale*_*BC*_ − *Ale*_*BC*,*act*_.
