# Supplementary material for: Mechanistic PK-PD model of alendronate treatment of postmenopausal osteoporosis predicts bone site-specific response
Source: Front Bioeng Biotechnol. 2022 Aug 17;10:940620. doi: 10.3389/fbioe.2022.940620 (PMC9428150; doi:10.3389/fbioe.2022.940620)
Supplement: Supplementary file 1 [file DataSheet1.pdf]

Supplementary Material to: Mechanistic PK-PD model of alendronate treatment of postmenopausal osteoporosis predicts bone site-specific response

José Luis Calvo-Gallego[a,*], Peter Pivonka[b], Rocío Ruiz-Lozano[a], Javier Martínez-Reina[a]

[a]*Departmento de Ingeniería Mecánica y Fabricación, Universidad de Sevilla, Seville 41092, Spain*

[b]*School of Mechanical, Medical and Process Engineering, Queensland University of Technology, QLD 4000, Australia.*

1. Introduction

In this document we provide a complete version of the mathematical model used in this work (the code in MATLAB is also accessible from this publication). With the intention of making a self-contained explanation, the mathematical development followed in the main document is enriched with the features which were not explained there. More precisely, we explain here the construction of the PK model (we also provide the differential equations governing the rest of the PK models which are not included in the main text), the bone cell population model based on the original model by Martin et al. [22] with the modifications introduced by Martínez-Reina et al. [25] and the inclusion of the alendronate PD model. The figures of the sensitivity analysis are also included at the end of this document.

This document is structured as follows: in section 2, the differential equations governing the PK models studied in this work are presented. Section 3 explains the bone cell population model with all its components, and therefore is divided in several subsections. In particular, in subsection 3.1 we present the general equations for competitive binding between ligands and receptors; the specific equations for the competitive binding of the complex RANK-RANKL-OPG are given in subsection 3.2, those for the com-

*Corresponding author: joselucalvo@us.es

plex Wnt–Scl–LRP5/6 are given in subsection 3.3 and the co-regulation of RANKL levels via the antagonistic effect of PTH and nitric oxide in subsection 3.4. The inclusion of damage in the model is explained in subsection 3.5. The upregulation of RANKL expressed by osteocytes due to damage is given in subsection 3.6. The equations related to the regulatory effect of TGF-β are included in subsection 3.7. Subsection 3.8 describes the term of proliferation of osteoblast precursors. The algorithm of bone mineralisation is explained in subsection 3.9. In subsection 3.10, the pharmacodynamic model of the alendronate is introduced. In subsection 3.11, the model constants are given in Table 1. Finally, in section 4, the figures of the sensitivity analysis conducted with the model parameters are presented.

2. Pharmacokinetics

In this work, we investigated 5 different PK models in order to find the most suitable one, which minimizes error between experimental data and model results. The schemes of the models can be seen in figures 1 and 2 (the model in the latter figure is separated as it was the final selected model). In the following, CC stands for the central compartment (plasma), NCT for the non-calcified tissues, BC for the bone compartment and IC for the inactive compartment, with the gut compartment added in the case of oral doses. The arrows in figures 1 and 2 indicate the flow of alendronate, k_i are the absorption rate constants and $k_{el,i}$ the elimination rate constants, with i = CC, NCT, BC, IC or urine. The studied models are:

1. One-compartment model with 2 elimination mechanisms, proposed by Chae et al. [5] (see Fig. 1a). This model has an important drawback. It is known from the literature that alendronate is only excreted by urine [38], but the model consists of a CC and 2 exits, one to urine and another to a non-specified organ (in the original model, the outflow rates were termed k_{urine} and $k_{non-urine}$). The latter was renamed here as k_{BC} in order to have an analogous terminology in all models. To overcome this drawback the following two-compartment model was proposed.
2. Two-compartment model. The only difference with the one-compartment model is that one of the exits from the CC flows into the BC and there is a return flow from BC to CC (see Fig. 1b).

3. Three-compartment model "in series". This model adds to the previous model a compartment connected to the BC, as seen in Fig. 1c. The model was called "in series" to distinguish it from the three-compartment model "in parallel". This model considers the different availability of the alendronate deposited in bone. To this end, it distinguishes the drug that has been deposited near the bone matrix – marrow interface from the alendronate that was buried deeper into the bone matrix. The former, termed as active alendronate (and contained in the BC), is more accessible for osteoclasts to resorb it, since resorption occurs mainly on the bone matrix – marrow interface. Thus, it can affect the osteoclastic activity through endocytosis. On the contrary, the latter is assumed to be inaccessible to resorption and was termed inactive alendronate (contained in the inactive compartment, IC) [38]. Cremers et al. [6] used a similar model for pamindronate.
4. Three-compartment model "in parallel". This model is similar to the previous model, but with the compartments arranged in a different layout, as can be seen in figure 2. The rationale for placing the compartments in parallel is that there are experimental results indicating that, once in the plasma, alendronate is quickly distributed into the non-calcified tissues of the body, followed by a redistribution in bone or renal elimination [21, 38].
5. Four-compartment model. It was proposed by Porras et al. [38], although they did not test it. It can be considered the combination of the three-compartment models "in parallel" and "in series", where the biological backgrounds of both apply (see Fig. 1d).

The differential equations which govern the temporal evolution of the alendronate in each model are presented below. In these equations, Ale_{CC}, Ale_{BC}, Ale_{NCT}, , Ale_{IC} Ale_{Gut} and Ale_{Urine} are the amount of alendronate in the central compartment, bone compartment, non-calcified tissues, inactive compartment, gut and urine respectively. F is the bioavailability. If the dose is administered intravenously, the gut compartment will not appear and therefore the term $F \cdot k_{CC} Ale_{Gut}$ in Eqs.(2, 5, 9, 14 and 19) must be replaced by the IV dose rate, while Eqs.(3, 7, 12, 17 and 23) are no longer needed. The concentration in the central compartment was calculated as:

$$[Ale_{CC}] = \frac{Ale_{CC}}{V_c} \tag{1}$$

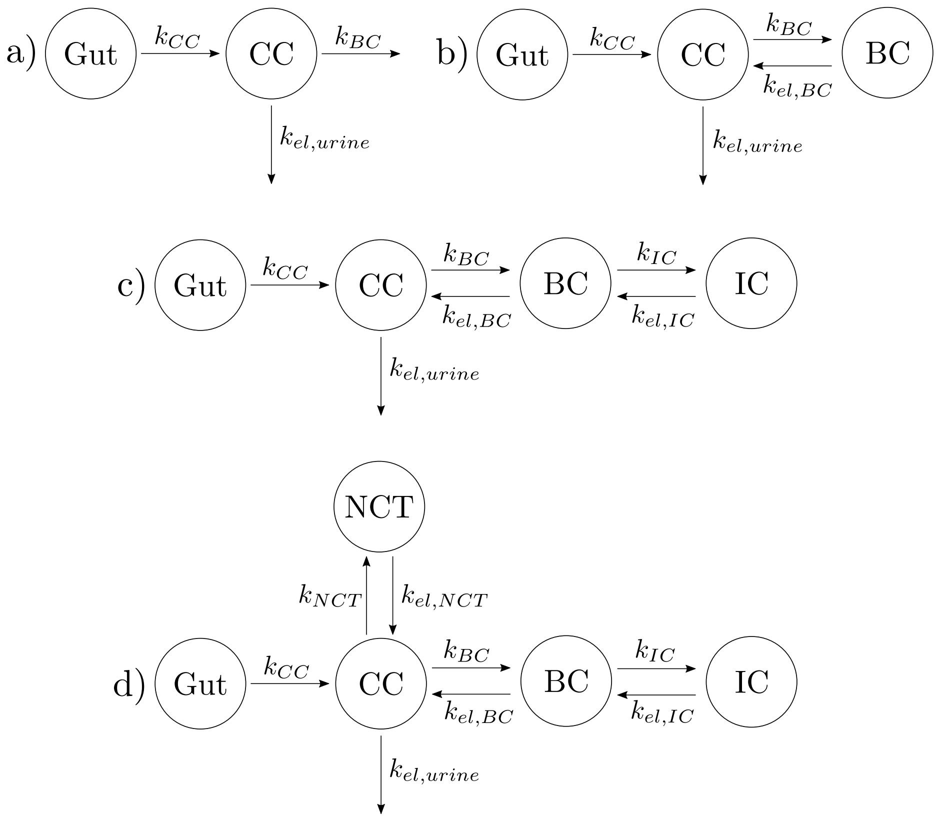

Figure 1: Different types of PK models for alendronate considering various numbers of compartments and different compartmental interactions. a) One-compartment model with 2 elimination mechanisms. b) Two-compartment model. c) Three-compartment model "in series". d) Four-compartment model.

where V_c is the volume of distribution of the central compartment.

The differential equations which govern the temporal evolution of the alendronate in the one-compartment model with two elimination mechanisms (see Fig. 1a) are:

$$\frac{dAle_{CC}}{dt} = F \cdot k_{CC} Ale_{Gut} - (k_{BC} + k_{el,urine}) Ale_{CC} \tag{2}$$

$$\frac{dAle_{Gut}}{dt} = -k_{CC} Ale_{Gut} \tag{3}$$

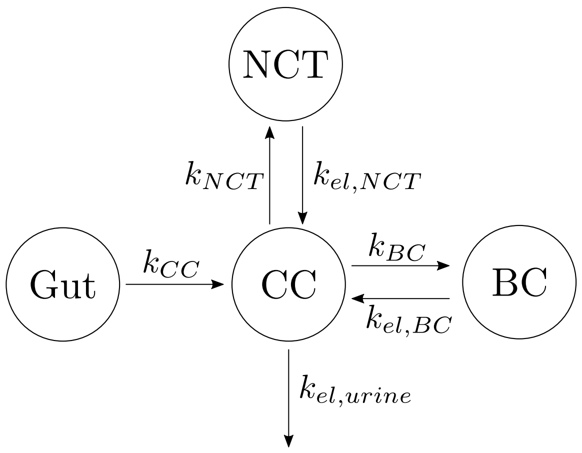

Figure 2: Alendronate three-compartment PK model "in parallel".

$$\frac{dAle_{Urine}}{dt} = k_{el,urine}Ale_{CC} \tag{4}$$

The differential equations which govern the temporal evolution of the alendronate in the two-compartment model (see Fig. 1b) are:

$$\frac{dAle_{CC}}{dt} = F \cdot k_{CC}Ale_{Gut} + k_{el,BC}Ale_{BC} - (k_{BC} + k_{el,urine})Ale_{CC} \tag{5}$$

$$\frac{dAle_{BC}}{dt} = k_{BC}Ale_{CC} - k_{el,BC}Ale_{BC} \tag{6}$$

$$\frac{dAle_{Gut}}{dt} = -k_{CC}Ale_{Gut} \tag{7}$$

$$\frac{dAle_{Urine}}{dt} = k_{el,urine}Ale_{CC} \tag{8}$$

The differential equations which govern the temporal evolution of the alendronate in the three-compartment model "in series" (see Fig. 1c) are:

$$\frac{dAle_{CC}}{dt} = F \cdot k_{CC}Ale_{Gut} + k_{el,BC}Ale_{BC} - (k_{BC} + k_{el,urine})Ale_{CC} \tag{9}$$

$$\frac{dAle_{BC}}{dt} = k_{BC}Ale_{CC} - (k_{el,BC} + k_{IC})Ale_{BC} + k_{el,IC}Ale_{IC} \tag{10}$$

$$\frac{dAle_{IC}}{dt} = k_{IC}Ale_{BC} - k_{el,IC}Ale_{IC} \quad (11)$$

$$\frac{dAle_{Gut}}{dt} = -k_{CC}Ale_{Gut} \quad (12)$$

$$\frac{dAle_{Urine}}{dt} = k_{el,urine}Ale_{CC} \quad (13)$$

The differential equations governing the temporal evolution of alendronate in the three-compartment PK model "in parallel" (see Fig. 2) are:

$$\begin{aligned}\frac{dAle_{CC}}{dt} &= F \cdot k_{CC}Ale_{Gut} + k_{el,BC}Ale_{BC} + k_{el,NCT}Ale_{NCT} \\ &- (k_{BC} + k_{NCT} + k_{el,urine})Ale_{CC}\end{aligned} \quad (14)$$

$$\frac{dAle_{BC}}{dt} = k_{BC}Ale_{CC} - k_{el,BC}Ale_{BC} \quad (15)$$

$$\frac{dAle_{NCT}}{dt} = k_{NCT}Ale_{CC} - k_{el,NCT}Ale_{NCT} \quad (16)$$

$$\frac{dAle_{Gut}}{dt} = -k_{CC}Ale_{Gut} \quad (17)$$

$$\frac{dAle_{Urine}}{dt} = k_{el,urine}Ale_{CC} \quad (18)$$

The differential equations which govern the temporal evolution of the alendronate in the four-compartment model (see Fig. 1d in the main document) are:

$$\begin{aligned}\frac{dAle_{CC}}{dt} &= F \cdot k_{CC}Ale_{Gut} + k_{el,NCT}Ale_{NCT} + k_{el,BC}Ale_{BC} - \\ &- (k_{NCT} + k_{BC} + k_{el,urine})Ale_{CC}\end{aligned} \quad (19)$$

$$\frac{dAle_{BC}}{dt} = k_{BC}Ale_{CC} + k_{el,IC}Ale_{IC} - (k_{el,BC} + k_{IC})Ale_{BC} \quad (20)$$

$$\frac{dAle_{NCT}}{dt} = k_{NCT}Ale_{CC} - k_{el,NCT}Ale_{NCT} \quad (21)$$

$$\frac{dAle_{IC}}{dt} = k_{IC}Ale_{BC} - k_{el,IC}Ale_{IC} \quad (22)$$

$$\frac{dAle_{Gut}}{dt} = -k_{CC}Ale_{Gut} \tag{23}$$

$$\frac{dAle_{Urine}}{dt} = k_{el,urine}Ale_{CC} \tag{24}$$

3. Bone cell population model

A previously published mathematical BCPM describing the bone cell interactions was used [22]. This model considers catabolic (RANK–RANKL–OPG) and anabolic (Wnt–Scl– LRP5/6) signalling pathways, together with the action of parathyroid hormone (PTH), nitric oxide (NO), transforming growth factor beta (TGF-β) and mechanobiological feedback on bone cells. The effect of bone mineralisation was added following Martínez-Reina and Pivonka [29] and Martínez-Reina et al. [24]. The accumulation and repair of microstructural damage was also taken into account as in Martínez-Reina et al. [27].

The bone cell types, whose concentrations are the state variables of the model, are: osteoblast precursor cells ($\mathrm{Ob_p}$), active osteoblasts ($\mathrm{Ob_a}$), osteoclast precursor cells ($\mathrm{Oc_p}$), active osteoclasts ($\mathrm{Oc_a}$) and osteocytes (Ot). The cell pools of uncommitted osteoblasts ($\mathrm{Ob_u}$) and osteoclasts ($\mathrm{Oc_u}$) are assumed constant as in [22].

$$\begin{aligned}\frac{d\,\mathrm{Ob_p}}{dt} &= D_{\mathrm{Ob_u}} \cdot \mathrm{Ob_u} \cdot \pi^{\mathrm{TGF-\beta}}_{\mathrm{act,Obu}} - D_{\mathrm{Ob_p}} \cdot \mathrm{Ob_p} \cdot \pi^{\mathrm{TGF-\beta}}_{\mathrm{rep,Ob_p}} \\ &+ P_{\mathrm{Ob_p}} \cdot \mathrm{Ob_p} \cdot \pi^{\mathrm{Wnt}}_{\mathrm{act,Ob_p}}\end{aligned} \tag{25}$$

$$\frac{d\,\mathrm{Ob_a}}{dt} = D_{\mathrm{Ob_p}} \cdot \mathrm{Ob_p} \cdot \pi^{\mathrm{TGF-\beta}}_{\mathrm{rep,Ob_p}} - \Delta_{\mathrm{Ob_a}} \cdot \mathrm{Ob_a} \tag{26}$$

$$\frac{d\,\mathrm{Oc_p}}{dt} = D_{\mathrm{Oc_u}} \cdot \mathrm{Oc_u} \cdot \pi^{\mathrm{RANKL}}_{\mathrm{act,Oc_u}} - D_{\mathrm{Oc_p}} \cdot \mathrm{Oc_p} \cdot \pi^{\mathrm{RANKL}}_{\mathrm{act,Oc_p}} \tag{27}$$

$$\frac{d\,\mathrm{Oc_a}}{dt} = D_{\mathrm{Oc_p}} \cdot \mathrm{Oc_p} \cdot \pi^{\mathrm{RANKL}}_{\mathrm{act,Oc_p}} - A_{\mathrm{Oc_a}} \cdot \mathrm{Oc_a} \cdot \pi^{\mathrm{TGF-\beta}}_{\mathrm{act,Oc_p}} \tag{28}$$

$$\frac{d\,\mathrm{Ot}}{dt} = \eta\,\frac{d\,f_{bm}}{dt} \tag{29}$$

where $D_{\mathrm{Ob_u}}$, $D_{\mathrm{Ob_p}}$, $D_{\mathrm{Oc_u}}$ and $D_{\mathrm{Oc_p}}$ are the differentiation rates of $\mathrm{Ob_u}$, $\mathrm{Ob_p}$, $\mathrm{Oc_u}$ and $\mathrm{Oc_p}$, respectively; $A_{\mathrm{Oc_a}}$ is the apoptosis rate of $\mathrm{Oc_a}$ and $\Delta_{\mathrm{Ob_a}}$ is

the rate of clearance of active osteoblasts through apoptosis or differentiation into osteocytes. The variables $\pi_{\mathrm{act,Obu}}^{\mathrm{TGF-\beta}}$, $\pi_{\mathrm{rep,Ob_p}}^{\mathrm{TGF-\beta}}$ and $\pi_{\mathrm{act,Oc_p}}^{\mathrm{TGF-\beta}}$ represent activator and repressor functions related to the binding of TGF-β to its receptor. Similarly, $\pi_{\mathrm{act,Oc_u}}^{\mathrm{RANKL}}$ and $\pi_{\mathrm{act,Oc_p}}^{\mathrm{RANKL}}$ are the activator functions related to the RANK-RANKL binding. Finally, $P_{\mathrm{Ob_p}}$ is the proliferation rate of $\mathrm{Ob_p}$, a process which is mediated by the Wnt signalling pathway through the activator function π_{act,Ob_p}^{Wnt}. These functions, as well as the remaining equations needed to complete the model, are described in the following subsections. The values of the model constants are given in Table 1.

Finally, Eq.(29) establishes that the population of osteocytes varies as the bone matrix fraction f_{bm}, if the density of osteocytes trapped within bone matrix, η, is assumed constant, as done in Martin et al. [22]. Bone matrix fraction is defined as the volume of bone matrix, V_b, per total volume of the bone sample (i.e. the representative volume element, V_{RVE}), expressed as a percentage, i.e.:

$$f_{bm}(\%) = \frac{V_b}{V_{RVE}} \cdot 100 \tag{30}$$

Its evolution is obtained through the balance between resorbed and formed tissue:

$$\frac{df_{bm}}{dt} = -k_{res} \cdot \mathrm{Oc_a} + k_{form} \cdot \mathrm{Ob_a} \tag{31}$$

where k_{res} and k_{form} are, respectively, the rates of bone resorption and osteoid formation.

3.1. Competitive binding

Many biological processes are controlled by binding of biochemical factors which act as receptor and ligand. In some of them two or more ligands compete to bind to the receptor. This is the case of Wnt and sclerostin that compete to bind to LRP5/6 to control the proliferation of osteoblast precursors and also the case of RANK and OPG which compete to bind to RANKL to control the differentiation of osteoclast precursors into mature active osteoclasts.

Let us consider separately the binding of a given receptor R to its ligands A and B to form, respectively, the complexes A–R and B–R. Let us consider

for each species X=A,B,R a production term P_{X} and a degradation term D_{X}, along with a degradation term for the complex $D_{\mathrm{X-Y}}$. Let $K^{r}_{\mathrm{X-Y}}$ and $K^{f}_{\mathrm{X-Y}}$ be the reverse and forward binding reaction constants, respectively.

$$\begin{array}{ccccccc} \downarrow P_{\mathrm{R}} & & \downarrow P_{\mathrm{A}} & & & & \\ \mathrm{R} & + & \mathrm{A} & \underset{K^{r}_{\mathrm{A-R}}}{\overset{K^{f}_{\mathrm{A-R}}}{\rightleftharpoons}} & \mathrm{A-R} & \overset{D_{\mathrm{A-R}}}{\rightharpoonup} & \emptyset \\ \downarrow D_{\mathrm{R}} & & \downarrow D_{\mathrm{A}} & & & & \end{array} \tag{32}$$

$$\begin{array}{ccccccc} \downarrow P_{\mathrm{R}} & & \downarrow P_{\mathrm{B}} & & & & \\ \mathrm{R} & + & \mathrm{B} & \underset{K^{r}_{\mathrm{B-R}}}{\overset{K^{f}_{\mathrm{B-R}}}{\rightleftharpoons}} & \mathrm{B-R} & \overset{D_{\mathrm{B-R}}}{\rightharpoonup} & \emptyset \\ \downarrow D_{\mathrm{R}} & & \downarrow D_{\mathrm{B}} & & & & \end{array} \tag{33}$$

The law of mass action provides the following set of differential equations:

$$\frac{\partial[\mathrm{A-R}]}{\partial t} = K^{f}_{\mathrm{A-R}}\,[\mathrm{A}]\,[\mathrm{R}] - K^{r}_{\mathrm{A-R}}\,[\mathrm{A-R}] - \tilde{D}_{\mathrm{A-R}}\,[\mathrm{A-R}] \tag{34a}$$

$$\frac{\partial[\mathrm{B-R}]}{\partial t} = K^{f}_{\mathrm{B-R}}\,[\mathrm{B}]\,[\mathrm{R}] - K^{r}_{\mathrm{B-R}}\,[\mathrm{B-R}] - \tilde{D}_{\mathrm{B-R}}\,[\mathrm{B-R}] \tag{34b}$$

$$\frac{\partial[\mathrm{A}]}{\partial t} = P_{\mathrm{A}} - \tilde{D}_{\mathrm{A}}\,[\mathrm{A}] + K^{r}_{\mathrm{A-R}}\,[\mathrm{A-R}] - K^{f}_{\mathrm{A-R}}\,[\mathrm{A}]\,[\mathrm{R}] \tag{34c}$$

$$\frac{\partial[\mathrm{B}]}{\partial t} = P_{\mathrm{B}} - \tilde{D}_{\mathrm{B}}\,[\mathrm{B}] + K^{r}_{\mathrm{B-R}}\,[\mathrm{B-R}] - K^{f}_{\mathrm{B-R}}\,[\mathrm{B}]\,[\mathrm{R}] \tag{34d}$$

$$\begin{aligned} \frac{\partial[\mathrm{R}]}{\partial t} =& P_{\mathrm{R}} - \tilde{D}_{\mathrm{R}}\,[\mathrm{R}] + K^{r}_{\mathrm{A-R}}\,[\mathrm{A-R}] + K^{r}_{\mathrm{B-R}}\,[\mathrm{B-R}] \\ & - K^{f}_{\mathrm{A-R}}\,[\mathrm{A}]\,[\mathrm{R}] - K^{f}_{\mathrm{B-R}}\,[\mathrm{B}]\,[\mathrm{R}] \end{aligned} \tag{34e}$$

where $[\mathrm{X}]$ and $[\mathrm{X-Y}]$ represent, respectively, the concentration of species X and complex X-Y. The degradation terms are assumed proportional to the concentration of the species, i.e. $D_{\mathrm{X}} = \tilde{D}_{\mathrm{X}}\,[\mathrm{X}]$, with $\tilde{D}_{\mathrm{X}}$ being the degradation rate.

Following Pivonka et al. [37] we assume that the binding reactions are much faster than the cell responses they produce and hence a quasi-steady state can be assumed, implying that the time derivatives of Eqs. (34) are null. This condition in Eqs. (34a) and (34b) yield:

$$[\mathrm{A}-\mathrm{R}] = \frac{[\mathrm{A}]\,[\mathrm{R}]}{K_{\mathrm{A}-\mathrm{R}}} \tag{35a}$$

$$[\mathrm{B}-\mathrm{R}] = \frac{[\mathrm{B}]\,[\mathrm{R}]}{K_{\mathrm{B}-\mathrm{R}}} \tag{35b}$$

where:

$$K_{\mathrm{A}-\mathrm{R}} = \frac{K^r_{\mathrm{A}-\mathrm{R}} + \tilde{D}_{\mathrm{A}-\mathrm{R}}}{K^f_{\mathrm{A}-\mathrm{R}}} \tag{36a}$$

$$K_{\mathrm{B}-\mathrm{R}} = \frac{K^r_{\mathrm{B}-\mathrm{R}} + \tilde{D}_{\mathrm{B}-\mathrm{R}}}{K^f_{\mathrm{B}-\mathrm{R}}} \tag{36b}$$

The stationarity condition of Eqs. (34c)-(34e) yields:

$$[\mathrm{A}] = \frac{P_{\mathrm{A}}}{\tilde{D}_{\mathrm{A}} + \frac{\tilde{D}_{\mathrm{A}-\mathrm{R}}}{K_{\mathrm{A}-\mathrm{R}}}\,[\mathrm{R}]} \tag{37a}$$

$$[\mathrm{B}] = \frac{P_{\mathrm{B}}}{\tilde{D}_{\mathrm{B}} + \frac{\tilde{D}_{\mathrm{B}-\mathrm{R}}}{K_{\mathrm{B}-\mathrm{R}}}\,[\mathrm{R}]} \tag{37b}$$

$$[\mathrm{R}] = \frac{P_{\mathrm{R}}}{\tilde{D}_{\mathrm{R}} + \frac{\tilde{D}_{\mathrm{A}-\mathrm{R}}}{K_{\mathrm{A}-\mathrm{R}}}\,[\mathrm{A}] + \frac{\tilde{D}_{\mathrm{B}-\mathrm{R}}}{K_{\mathrm{B}-\mathrm{R}}}\,[\mathrm{B}]} \tag{37c}$$

If the degradation ($\tilde{D}_{\mathrm{X}}$,$\tilde{D}_{\mathrm{X}-\mathrm{Y}}$), production ($P_{\mathrm{X}}$) and dissociation constants, ($K_{\mathrm{X}-\mathrm{Y}}$) are known, (37) constitutes a non-linear system of three equations with three unknowns, namely [A], [B], [R].

The total concentration of a receptor is the sum of the concentrations of the receptor which is found free and bound to ligands, i.e.:

$$[\mathrm{R}]_{\mathrm{tot}} = [\mathrm{R}] + [\mathrm{A}-\mathrm{R}] + [\mathrm{B}-\mathrm{R}] = [\mathrm{R}]\left(1 + \frac{[\mathrm{A}]}{K_{\mathrm{A}-\mathrm{R}}} + \frac{[\mathrm{B}]}{K_{\mathrm{B}-\mathrm{R}}}\right) \tag{38}$$

where Eqs. (35) have been used. The stationarity condition is equivalent to establish that the production rate of a species must equal the degradation rate, including all its forms, free and bound. For instance, in the case of the receptor that can bind to different ligands, this condition reads:

$$P_{\mathrm{R}} = \tilde{D}_{\mathrm{R}}\,[\mathrm{R}] + \sum_{\mathrm{L}} \tilde{D}_{\mathrm{L-R}}\,[\mathrm{L-R}] \tag{39}$$

which can be obtained from Eqs. (34a), (34b) and (34e) by imposing that the stationarity condition is met, ($\frac{\partial[\mathrm{R}]}{\partial t} = \frac{\partial[\mathrm{L-R}]}{\partial t} = 0 \quad \forall\,\mathrm{L} = \mathrm{A}, \mathrm{B}$). In the case of a ligand, that only binds to the receptor, that condition reads:

$$P_{\mathrm{L}} = \tilde{D}_{\mathrm{L}}\,[\mathrm{L}] + \tilde{D}_{\mathrm{L-R}}\,[\mathrm{L-R}] \tag{40}$$

The degradation rates are usually assumed as constants but the production rates are modelled in a more complex way. For instance, the production rate of ligand L can be split into a term corresponding to endogenous production, $P_{\mathrm{L,b}}$, and a term accounting for external dosage, $P_{\mathrm{L,d}}$:

$$P_{\mathrm{L}} = P_{\mathrm{L,b}} + P_{\mathrm{L,d}} \tag{41}$$

The endogenous production is sometimes modelled by the following equation:

$$P_{\mathrm{L,b}} = \sum_{\mathrm{X,Y}} \beta_{\mathrm{L,Y}}\,\pi^{\mathrm{X}}_{\mathrm{act/rep,Y}}\,\mathrm{Y}\left(1 - \frac{[\mathrm{L}]}{[\mathrm{L}]_{\mathrm{max}}}\right) \tag{42}$$

where Y is the concentration of the cell type Y producing L with a production rate $\beta_{\mathrm{L,Y}}$, regulated by the species X through the activator or repressor function $\pi^{\mathrm{X}}_{\mathrm{act/rep,Y}}$. The parenthesis establishes a saturation condition in such a way that ligand is not produced if its concentration reaches the maximum or saturation value, $[\mathrm{L}]_{\mathrm{max}}$.

As described by Pivonka et al. [35] the activation of a certain biological process regulated by the formation of the complex L–R is given by the ratio between the receptors R occupied by ligands L and the total number of receptors:

$$\pi^{\mathrm{L}}_{\mathrm{act,Y}} = \frac{[\mathrm{L-R}]}{[\mathrm{R}]_{\mathrm{tot}}} = \frac{[\mathrm{L-R}]}{[\mathrm{R}] + \sum_{\mathrm{L'}} [\mathrm{L'-R}]} \tag{43}$$

Similarly, the repressor action of the binding is given by the complementary to one of the latter:

$$\pi_{\mathrm{rep,Y}}^{\mathrm{L}} = \frac{[\mathrm{R}]_{\mathrm{tot}} - [\mathrm{L-R}]}{[\mathrm{R}]_{\mathrm{tot}}} = \frac{[\mathrm{R}] + \sum_{\mathrm{L}' \neq \mathrm{L}} [\mathrm{L}' - \mathrm{R}]}{[\mathrm{R}] + \sum_{\mathrm{L}'} [\mathrm{L}' - \mathrm{R}]} \tag{44}$$

In case of a single ligand the latter expressions yield the first-order Hill activator and repressor functions:

$$\pi_{\mathrm{act,Y}}^{\mathrm{L}} = \frac{[\mathrm{L}]}{[\mathrm{L}] + K_{\mathrm{act,Y}}^{\mathrm{L-R}}} \tag{45}$$

$$\pi_{\mathrm{rep,Y}}^{\mathrm{L}} = \frac{K_{\mathrm{rep,Y}}^{\mathrm{L-R}}}{K_{\mathrm{rep,Y}}^{\mathrm{L-R}} + [\mathrm{L}]} \tag{46}$$

3.2. Competitive RANK-RANKL-OPG binding

The RANK-RANKL-OPG signalling pathway controls the differentiation of uncommitted osteoclast progenitors and osteoclasts maturation, respectively through π_{act,Oc_u}^{RANKL} and π_{act,Oc_p}^{RANKL} (see Eqs.10 and 11 in the main document). Thus, an imbalance in that pathway, such as that occurring after menopause, may result in the development of osteoporosis. Following Martin et al. [22], the concentrations of OPG, RANK and RANKL are given by the following equations:

$$[\mathrm{OPG}] = \frac{P_{\mathrm{OPG}}}{\tilde{D}_{\mathrm{OPG}} + \frac{\tilde{D}_{\mathrm{OPG-RANKL}}[\mathrm{RANKL}]}{K_{\mathrm{OPG-RANKL}}}} \tag{47}$$

$$[\mathrm{RANK}] = \frac{N_{\mathrm{Oc_p}}^{\mathrm{RANK}}\,\mathrm{Oc_p}}{1 + \frac{[\mathrm{RANK}]}{K_{\mathrm{RANK-RANKL}}}} \tag{48}$$

$$\begin{aligned}[\mathrm{RANKL}] =& P_{\mathrm{RANKL}} \cdot \left[\tilde{D}_{\mathrm{RANKL}} + \frac{\tilde{D}_{\mathrm{OPG-RANKL}}}{K_{\mathrm{OPG-RANKL}}} \cdot [\mathrm{OPG}]\right. \\ & \left. + \frac{\tilde{D}_{\mathrm{RANK-RANKL}}}{K_{\mathrm{RANK-RANKL}}} \cdot [\mathrm{RANK}]\right]^{-1}\end{aligned} \tag{49}$$

where $\tilde{D}_{\mathrm{X}}$ and $\tilde{D}_{\mathrm{X-Y}}$ are the degradation rates of the factor X and the complex X-Y, respectively; $K_{\mathrm{X-Y}}$ is the dissociation constant of the complex X-Y and $N_{\mathrm{Oc_p}}^{\mathrm{RANK}}$ is the number of RANK receptors per osteoclast precursor. P_{OPG} is the production rate of OPG by active osteoblasts:

$$P_{\mathrm{OPG}} = \beta_{\mathrm{OPG,Ob_a}}\, \pi_{\mathrm{rep,Ob_a}}^{\mathrm{PTH}}\, \mathrm{Ob_a} \left(1 - \frac{[\mathrm{OPG}]}{[\mathrm{OPG_{max}}]}\right) \tag{50}$$

where $\beta_{\mathrm{OPG,Ob_a}}$ is the OPG production rate, $\pi_{\mathrm{rep,Ob_a}}^{\mathrm{PTH}}$ is the repressor function that quantifies the effect of PTH on the production of OPG and $[\mathrm{OPG_{max}}]$ is the saturation concentration of OPG above which no further production takes place. To evaluate P_{RANKL}, the RANKL production rate of Eq. (49), we have assumed that RANKL is expressed by osteocytes and osteoblasts precursors, following experimental evidence [30, 44] and then:

$$\begin{aligned} P_{\mathrm{RANKL}} = {} & \beta_{\mathrm{RANKL,Ot}}\, \mathrm{Ot} \left(1 - \frac{[\mathrm{RANKL}]_{\mathrm{tot}}}{[\mathrm{RANKL}]_{\mathrm{max}}}\right) \pi_{\mathrm{act,RANKL}}^{\mathrm{dam}} \\ & + \beta_{\mathrm{RANKL,Ob_p}}\, \pi_{\mathrm{act/rep,RANKL}}^{\mathrm{PTH,NO}}\, \mathrm{Ob_p} \left(1 - \frac{[\mathrm{RANKL}]_{\mathrm{tot}}}{[\mathrm{RANKL}]_{\mathrm{max}}}\right) + P_{\mathrm{RANKL}}^{\mathrm{PMO}} \end{aligned} \tag{51}$$

where $P_{\mathrm{RANKL}}^{\mathrm{PMO}}$ is the RANKL production due to PMO, which expression is given in the main document; $\beta_{\mathrm{RANKL,Ot}}$ and $\beta_{\mathrm{RANKL,Ob_p}}$ are the RANKL production rate of osteocytes and osteoblast precursors, respectively; $\pi_{\mathrm{act/rep,RANKL}}^{\mathrm{PTH,NO}}$ is a co-regulatory function that takes into account the up-regulation of RANKL transcription by the parathyroid hormone (PTH) and its inhibition by nitric oxide (NO) [22] and $\pi_{\mathrm{act,RANKL}}^{\mathrm{dam}}$ is an activator function accounting for the upregulation of RANKL expression by osteocytes due to microstructural damage [27] (see the details of both regulatory functions in sections 3.4 and 3.6 respectively). Finally, $[\mathrm{RANKL}]_{\mathrm{max}}$ is the saturation concentration of RANKL above which no further expression takes place and $[\mathrm{RANKL}]_{\mathrm{tot}}$ is the total concentration of RANKL (bound and free) and is defined as follows:

$$\begin{aligned} [\mathrm{RANKL}]_{\mathrm{tot}} = {} & [\mathrm{RANKL}] \cdot \\ & \left(1 + \frac{[\mathrm{OPG}]}{K_{\mathrm{OPG-RANKL}}} + \frac{[\mathrm{RANK}]}{K_{\mathrm{RANK-RANKL}}} + \frac{[\mathrm{Dmab}]_{\mathrm{BC}}}{K_{\mathrm{RANKL-Dmab}}}\right) \end{aligned} \tag{52}$$

Eqs. (51) and (52) can be substituted in (49) to work out the free RANKL concentration, i.e. [RANKL]. Then, the activator functions in Eqs.10 and 11 in the main document have the same structure:

$$\pi_{\mathrm{act,X}}^{\mathrm{RANKL}} = \frac{[\mathrm{RANKL}]}{K_{\mathrm{act,X}}^{\mathrm{RANKL}} + [\mathrm{RANKL}]} \qquad \text{with X} = \mathrm{Oc_u},\ \mathrm{Oc_p} \tag{53}$$

and different constants: $K_{\mathrm{act,Oc_u}}^{\mathrm{RANKL}}$ and $K_{\mathrm{act,Oc_p}}^{\mathrm{RANKL}}$, as in Martínez-Reina et al. [25]. In previous works [29, 26, 27] both constants $K_{\mathrm{act,X}}^{\mathrm{RANKL}}$ were chosen equal, so resulting in a constant pool of $\mathrm{Oc_p}$ in the steady state.

3.3. Competitive binding Wnt–Scl–LRP5/6

Wnt signaling is an anabolic pathway promoting the proliferation of osteoblast precursors and hence bone formation. Extracellular Wnt binds to Frizzled and the lipoprotein receptor-related protein LRP5/6, so triggering intracellular activation of β-catenin. Sclerostin, produced by osteocytes, modulates the signaling pathway by its interaction with LRP5/6 receptors. This prevents the formation of the Wnt-Frizzled-LRP5/6 complex and therefore hinders preosteoblasts proliferation. Competitive Wnt–Scl–LRP5/6 binding is modelled as follows. First, Eq. (38) reads for LRP5/6:

$$[\mathrm{LRP5/6}]_{\mathrm{tot}} = [\mathrm{LRP5/6}] \cdot \left(1 + \frac{[\mathrm{Wnt}]}{K_{\mathrm{Wnt-LRP5/6}}} + \frac{[\mathrm{Scl}]}{K_{\mathrm{Scl-LRP5/6}}}\right) \tag{54}$$

The production of sclerostin is given by an equation like (40), which now reads:

$$P_{\mathrm{Scl,b}} + P_{\mathrm{Scl,d}} = \tilde{D}_{\mathrm{Scl}}\,[\mathrm{Scl}] + \tilde{D}_{\mathrm{Scl-LRP5/6}}\,[\mathrm{Scl} - \mathrm{LRP5/6}] \tag{55}$$

where $\tilde{D}_{\mathrm{Scl}}$ and $\tilde{D}_{\mathrm{Scl-LRP5/6}}$ are the degradation rates of sclerostin and the sclerostin-LRP5/6 complex, respectively. The concentration of this complex is given by the receptor-ligand binding equation (34a), which reads here:

$$[\mathrm{Scl} - \mathrm{LRP5/6}] = \frac{[\mathrm{Scl}]\,[\mathrm{LRP5/6}]}{K_{\mathrm{Scl-LRP5/6}}} \tag{56}$$

The external dosage of sclerostin, $P_{\mathrm{Scl,d}}$, is set to zero and the endogenous production of sclerostin by osteocytes is:

$$P_{\mathrm{Scl,b}} = \beta_{\mathrm{Scl,Ot}}\, \pi_{\mathrm{rep,Scl}}^{\Psi_{\mathrm{bm}}}\, \mathrm{Ot} \left(1 - \frac{[\mathrm{Scl}]}{[\mathrm{Scl}]_{\mathrm{max}}}\right) \tag{57}$$

where $\beta_{Scl,Ot}$ and $[\mathrm{Scl}]_{\mathrm{max}}$ are, respectively, the sclerostin production rate and its maximum concentration. The production of sclerostin by osteocytes is downregulated by the mechanical stimulus through the repressor function $\pi_{\mathrm{rep,Scl}}^{\Psi_{\mathrm{bm}}}$ (see Eq. (74) later on). Replacing (57) and (56) into (55) yields:

$$\beta_{\mathrm{Scl,Ot}}\, \pi_{\mathrm{rep,Scl}}^{\Psi_{\mathrm{bm}}}\, \mathrm{Ot} \left(1 - \frac{[\mathrm{Scl}]}{[\mathrm{Scl}]_{\mathrm{max}}}\right) = \tilde{D}_{\mathrm{Scl}}\, [\mathrm{Scl}] + \tilde{D}_{\mathrm{Scl-LRP5/6}}\, \frac{[\mathrm{Scl}]\, [\mathrm{LRP5/6}]}{K_{\mathrm{Scl-LRP5/6}}} \tag{58}$$

Following Martin et al. [22] we assumed that the total number of LRP5/6 receptors per osteoblast precursor ($N_{\mathrm{OB_p}}^{\mathrm{LRP5/6}}$) is constant and thus:

$$[\mathrm{LRP5/6}]_{\mathrm{tot}} = N_{\mathrm{OB_p}}^{\mathrm{LRP5/6}}\, \mathrm{Ob_p} \tag{59}$$

Solving for [LRP5/6] in Eq. (54) and replacing it into (58) yields the following second-order polynomial of the free sclerostin, [Scl]:

$$A\, [\mathrm{Scl}]^2 + B\, [\mathrm{Scl}] + C = 0 \tag{60}$$

where the constants coefficients of the polynomial are:

$$A = \tilde{D}_{\mathrm{Scl}} + \frac{\beta_{\mathrm{Scl,Ot}}\, \pi_{\mathrm{rep,Scl}}^{\Psi_{\mathrm{bm}}}\, \mathrm{Ot}}{[\mathrm{Scl}]_{\mathrm{max}}} \tag{61}$$

$$\begin{aligned} B =& A \cdot K_{\mathrm{Scl-LRP5/6}} \left(1 + \frac{[\mathrm{Wnt}]}{K_{\mathrm{Wnt-LRP5/6}}}\right) + \tilde{D}_{\mathrm{Scl-LRP5/6}}\, [\mathrm{LRP5/6}]_{\mathrm{tot}} \\ & - (P_{\mathrm{Scl,d}} + \beta_{\mathrm{Scl,Ot}}\, \pi_{\mathrm{rep,Scl}}^{\Psi_{\mathrm{bm}}}\, \mathrm{Ot}) \end{aligned} \tag{62}$$

$$C = - K_{\mathrm{Scl-LRP5/6}} \left(1 + \frac{[\mathrm{Wnt}]}{K_{\mathrm{Wnt-LRP5/6}}}\right) (P_{\mathrm{Scl,d}} + \beta_{\mathrm{Scl,Ot}}\, \pi_{\mathrm{rep,Scl}}^{\Psi_{\mathrm{bm}}}\, \mathrm{Ot}) \tag{63}$$

$$(64)$$

We can use (59) in the previous expression together with the previously calculated cell populations and [Wnt], which is assumed constant, to work out the three coefficients. Only one solution of (60) is positive as shown by Martin et al. [22] and this solution [Scl] is then used in (54) to calculate [LRP5/6]. Finally, using Eqs. (43) and (59), the activator function in the Ob_p proliferation term can be calculated as:

$$\pi_{\mathrm{act,Ob_p}}^{\mathrm{Wnt}} = \frac{[\mathrm{Wnt-LRP5/6}]}{[\mathrm{LRP5/6}]_{\mathrm{tot}}} = \frac{[\mathrm{Wnt}]\,[\mathrm{LRP5/6}]}{K_{\mathrm{Wnt-LRP5/6}}\,[\mathrm{LRP5/6}]_{\mathrm{tot}}} \tag{65}$$

Some studies have shown an increase in serum sclerostin after menopause [1, 16], while sclerostin expression (local mRNA levels) was found to decrease in animal models of menopause [16]. Following [22] we have assumed an exponential decay of the degradation rate of sclerostin to acknowledge this discrepancy between the serum levels and the local expression of sclerostin:

$$\tilde{D}_{\mathrm{Scl}}(t) = \begin{cases} \tilde{D}_{\mathrm{Scl}}^{0}\exp\left(\dfrac{t-t_{\mathrm{onset}}}{\tau_{PMO}}\right) & \text{for } t \geq t_{\mathrm{onset}} \\ \tilde{D}_{\mathrm{Scl}}^{0} & \text{for } t < t_{\mathrm{onset}} \end{cases} \tag{66}$$

where t_{onset} is the time of onset of menopause, $\tilde{D}_{\mathrm{Scl}}^{0}$ is the pre-menopause value of the degradation rate of sclerostin and τ_{PMO} is a time constant.

3.4. Co-regulation of RANKL via PTH and NO concentration

RANKL transcription is upregulated by parathyroid hormone (PTH) and downregulated by nitric oxide (NO). In the model developed by Martin et al. [22] this antagonistic influence was merged into a co-regulatory function capturing both effects.

$$\pi_{\mathrm{act/rep,RANKL}}^{\mathrm{PTH,NO}} = \lambda_s\left(\pi_{\mathrm{act,RANKL}}^{\mathrm{PTH}} + \pi_{\mathrm{rep,RANKL}}^{\mathrm{NO}}\right) + \lambda_c\,\pi_{\mathrm{act,RANKL}}^{\mathrm{PTH}}\cdot\pi_{\mathrm{rep,RANKL}}^{\mathrm{NO}} \tag{67}$$

where the activator function accounting for the effect of PTH is:

$$\pi_{\text{act,RANKL}}^{\text{PTH}} = \frac{[\text{PTH}]}{[\text{PTH}] + K_{\text{act}}^{\text{PTH}}} \tag{68}$$

and the repressor effect on OPG (see Eq.(50)) is accounted for through the function:

$$\pi_{\text{rep,Ob}_\text{a}}^{\text{PTH}} = \frac{K_{\text{rep}}^{\text{PTH}}}{[\text{PTH}] + K_{\text{rep}}^{\text{PTH}}} \tag{69}$$

being $K_{\text{act}}^{\text{PTH}}$ and $K_{\text{rep}}^{\text{PTH}}$ constants and the concentration of PTH given by:

$$[\text{PTH}] = \frac{\beta_{\text{PTH}}}{\tilde{D}_{\text{PTH}}} \tag{70}$$

which comes from Eqs. (39) to (42) when there is no ligand for the species, the external dosage is null ($P_{\text{PTH,d}} = 0$), the endogenous production rate is not regulated ($\pi_{\text{act/rep,Y}}^{\text{X}} \cdot \text{Y} = 1$ see Eq. (42)) and the saturation value $[\text{PTH}]_{\text{max}}$ is large enough to assume the parenthesis equal to 1. β_{PTH} and $\tilde{D}_{\text{PTH}}$ are the endogenous production and degradation rate of PTH, respectively. On the other hand, the factor corresponding to nitric oxide is:

$$\pi_{\text{rep,RANKL}}^{\text{NO}} = \frac{K_{\text{rep}}^{\text{NO}}}{[\text{NO}] + K_{\text{rep}}^{\text{NO}}} \tag{71}$$

with $K_{\text{rep}}^{\text{NO}}$ a constant and the concentration of NO given by:

$$[\text{NO}] = \frac{P_{\text{NO,d}} + \beta_{\text{NO,Ot}}\, \pi_{\text{act,NO}}^{\Psi_{\text{bm}}}\, \text{Ot}}{\tilde{D}_{\text{NO}} + \frac{\beta_{\text{NO,Ot}}\, \pi_{\text{act,NO}}^{\Psi_{\text{bm}}}\, \text{Ot}}{[\text{NO}]_{\text{max}}}} \tag{72}$$

which also comes from Eq. (39) in the absence of ligands. The external dosage of nitric oxide $P_{\text{NO,d}}$ is set to zero in this study, $\beta_{\text{NO,Ot}}$, $\tilde{D}_{\text{NO}}$ and $[\text{NO}]_{\text{max}}$ are, respectively, the endogenous production and degradation rate of nitric oxide and its maximum content. The factor $\pi_{\text{act,NO}}^{\Psi_{\text{bm}}}$ is the mechanical

feedback activator function that accounts for the production of NO by osteocytes. This function and the repressor function affecting the production of sclerostin by osteocytes (see Eq. (57)) are defined by the following sigmoidal functions:

$$\pi_{\mathrm{act,NO}}^{\Psi_{\mathrm{bm}}} = \rho_{\mathrm{act}} + \frac{\left(\alpha_{\mathrm{act}} - \rho_{\mathrm{act}}\right) \Psi_{\mathrm{bm}}^{\gamma^{\mathrm{act}}}}{\delta_{\mathrm{act}}^{\gamma^{\mathrm{act}}} + \Psi_{\mathrm{bm}}^{\gamma^{\mathrm{act}}}} \tag{73}$$

$$\pi_{\mathrm{rep,Scl}}^{\Psi_{\mathrm{bm}}} = \alpha_{\mathrm{rep}} - \frac{\left(\alpha_{\mathrm{rep}} - \rho_{\mathrm{rep}}\right) \Psi_{\mathrm{bm}}^{\gamma^{\mathrm{rep}}}}{\delta_{\mathrm{rep}}^{\gamma^{\mathrm{rep}}} + \Psi_{\mathrm{bm}}^{\gamma^{\mathrm{rep}}}} \tag{74}$$

where $\rho_{\sim}$ and $\alpha_{\sim}$ are, respectively, the minimum and maximum anticipated response, $\gamma^{\sim}$ is the sigmoidicity, influencing the steepness of the response, and $\delta_{\sim}$ is the value of the stimulus producing the half-maximal response [34].

Finally, the strain energy density (SED) at the bone matrix level is given by the SED at the continuum level, $\Psi = \frac{1}{2}\boldsymbol{\sigma} : \boldsymbol{\varepsilon}$, and the bone matrix fraction through the following expression proposed by Beaupre et al. [2]:

$$\Psi_{\mathrm{bm}} = \frac{\Psi}{\left(\frac{f_{\mathrm{bm}}}{100}\right)^2} \tag{75}$$

3.5. Damage

Targeted bone remodelling theories hypothesise that one of the major functions of bone remodelling is to remove microcracks from bone matrix, so avoiding an excessive accumulation of the latter, which could result in macroscopic failure [32]. The accumulation of microcracks in a particular volume of material is addressed here using a Continuum Damage Mechanics approach [20]. This theory introduces a damage variable, d, which is linked to the density of microcracks in a volume of material and to the loss of stiffness through Eq. (76). This variable is such that $d \in [0, 1]$, with $d = 0$ corresponding to an undamaged state and $d = 1$ to a local fracture or failure situation:

$$\mathbf{C} = (1 - d)\,\mathbf{C}_0 \tag{76}$$

where $\mathbf{C}$ and $\mathbf{C}_0$ are, respectively, the stiffness tensors of damaged and undamaged bone [20]. In the isotropic damage theory, Eq. (76) can be rewritten in terms of the respective Young's moduli, E and E_0, as $E = (1-d)\,E_0$ [46, 33] (see Eq. (104)).

A balance of microdamage is considered through the accumulation due to fatigue loading and the removal due to bone remodelling, as osteoclasts resorb the damaged tissue, while the osteoid deposited by osteoblasts is initially intact. The evolution law for damage can be expressed as:

$$\dot{d} = \dot{d}_A - \dot{d}_R \tag{77}$$

where $\dot{d}_A$ is the rate of damage accumulation by fatigue loading and $\dot{d}_R$ is the rate of damage removal by bone remodelling. The latter is assessed by assuming that damage is uniformly distributed throughout the representative volume element (RVE). So, the amount of repaired damage is proportional to the damage present in that volume and to the volume of tissue being resorbed, $\dot{V}_r$, through the fraction that this volume represents within the bone matrix volume:

$$\dot{d}_R = d\,\frac{\dot{V}_r}{V_{bm}} = d\,\frac{k_{res}\cdot Oc_a}{f_{bm}} \tag{78}$$

Damage accumulation is evaluated following the procedure described in [24, 28]. This procedure makes use of the results of the experimental fatigue tests performed by Pattin et al. [33], who provided the evolution of damage with the strain level and the number of cycles. This evolution was mathematically modelled by García-Aznar et al. [11] to yield the following differential equation under tensile stresses:

$$\dot{d}_a = \dot{N}\,\frac{C_1}{C_2\,\gamma_f}\,(1-d)^{1-\gamma_f}\;\varepsilon_{max}^{\delta_f}\,\exp(-C_2\,(1-d)^{\gamma_f}) \tag{79}$$

where $\dot{N}$ is the number of cycles applied per unit time and ε_{max} is the maximum principal strain expressed in $\mu\varepsilon$.[1] The rest of parameters and constants

[1] In the damage model proposed by Martínez-Reina et al. [28], cracks were assumed to grow normal to the maximum strain direction and only under tensile strains.

of the model are:

$$C_1 = \frac{e^{C_2} - 1}{K_f([Ca])}; \qquad \delta_f = 14.1;$$
$$\gamma_f = -0.018(\varepsilon_{max} - 4100) + 12; \qquad C_2 = -20; \tag{80}$$

where $K_f([Ca])$ is a function of the mineral content which will be defined next. The experimental tests perfomed by Pattin et al. [33] included an estimation of fatigue life, N_f, which was related to the deformation by the following expression:

$$N_f = \frac{K_f}{\varepsilon_{max}^{\delta_f}} \tag{81}$$

where K_f was assumed constant and equal to $1.445 \cdot 10^{53}$ in tension. Martínez-Reina et al. [24] introduced a correction in K_f to consider the degradation of the fatigue properties with the increase in mineral content. A life $N_f = 10^7$ cycles was assigned to the fatigue limit, which is usually assumed to occur for a given fraction of the ultimate tensile strain, ε_u/β, where the parameter β depends on the type of material [17] and $\beta = 2$ was assumed for bone [24] with good results. So, K_f was obtained from Eq. (81) as:

$$K_f([Ca]) = 10^7 \left(\frac{\varepsilon_u([Ca])}{\beta} \right)^{\delta_f} \tag{82}$$

where the ultimate tensile strain depends on the calcium concentration of bone matrix, $[Ca]$, as Currey [7] showed. The following regression was fitted in [24] from the experimental results presented by Currey [7]:

$$log\, \varepsilon_u = 31.452 - 11.341\, log\, [Ca] \tag{83}$$

where ε_u is expressed in $\mu\varepsilon$ and the concentration $[Ca]$ is expressed in mg of calcium per g of bone matrix. This concentration is directly related to the ash fraction, α, which will be defined in the next section. More precisely, the relation $[Ca] = 398.8\, \alpha$ was assumed, based on the molecular weigths of hydroxyapatite and type I collagen [24].

3.6. Upregulation of RANKL expressed by osteocytes due to microstructural damage

As proposed in [27] we have assumed that RANKL expression by osteocytes is upregulated by the presence of microstructural damage in the bone matrix through the factor $\pi_{\text{act,RANKL}}^{\text{dam}}$, which is defined as a sigmoidal function of damage, d:

$$\pi_{\text{act,RANKL}}^{\text{dam}} = \rho_{dam} + (\alpha_{dam} - \rho_{dam})\,(1 + K_{dam})\,\frac{d}{K_{dam} + d} \tag{84}$$

where K_{dam} is a constant, ρ_{dam} is the minimum value of the factor $\pi_{\text{act,RANKL}}^{\text{dam}}$, corresponding to $d = 0$, while α_{dam} is its maximum value, corresponding to $d = 1$.

3.7. Regulatory role of TGF-β

TGF–β is stored in the bone matrix and released during resorption by osteoclasts. Its concentration is calculated following Pivonka et al. [37]:

$$[\text{TGF}-\beta] = \frac{\alpha_{\text{TGF}-\beta}\; k_{res}\; Oc_a}{\tilde{D}_{\text{TGF}-\beta}} \tag{85}$$

where $\alpha_{\text{TGF}-\beta}$ is the concentration of TGF–β in bone matrix and $\tilde{D}_{\text{TGF}-\beta}$ is the TGF–β degradation rate. The concentration of TGF–β is used to define the activator/repressor functions in Eqs. (8)-(11) of the main document. These functions control the upregulation of the differentiation of Ob_u into Ob_p, the upregulation of osteoclast apoptosis and the downregulation of the differentiation of Ob_p into Ob_a:

$$\pi_{\text{act,Ob}_\text{u}}^{\text{TGF}-\beta} = \pi_{\text{act,Oc}_\text{p}}^{\text{TGF}-\beta} = \frac{[\text{TGF}-\beta]}{K_{\text{act}}^{\text{TGF}-\beta} + [\text{TGF}-\beta]} \tag{86}$$

$$\pi_{\text{rep,Ob}_\text{p}}^{\text{TGF}-\beta} = \frac{K_{\text{rep}}^{\text{TGF}-\beta}}{K_{\text{rep}}^{\text{TGF}-\beta} + [\text{TGF}-\beta]} \tag{87}$$

with $K_{\text{act}}^{\text{TGF}-\beta}$ and $K_{\text{rep}}^{\text{TGF}-\beta}$ the activation and repression constants, respectively.

3.8. *Proliferation of osteoblast precursors*

Let us recall the differential equation of osteoblast precursors.

$$\frac{d\,\mathrm{Ob_p}}{dt} = D_{\mathrm{Ob_u}} \cdot \mathrm{Ob_u} \cdot \pi_{\mathrm{act,Ob_u}}^{\mathrm{TGF-\beta}} - D_{\mathrm{Ob_p}} \cdot \mathrm{Ob_p} \cdot \pi_{\mathrm{rep,Ob_p}}^{\mathrm{TGF-\beta}} + P_{\mathrm{Ob_p}} \cdot \mathrm{Ob_p} \cdot \pi_{\mathrm{act,Ob_p}}^{\mathrm{Wnt}} \tag{88}$$

We can rewrite this equation as:

$$\frac{d\,\mathrm{Ob_p}}{dt} = \mathcal{D}_{\mathrm{Ob_u}} \cdot \mathrm{Ob_u} - \mathcal{D}_{\mathrm{Ob_p}} \cdot \mathrm{Ob_p} + \mathcal{P}_{\mathrm{Ob_p}} \cdot \mathrm{Ob_p} \tag{89}$$

where the terms in the right-hand side correspond, respectively, to the differentiation of $\mathrm{Ob_u}$ into $\mathrm{Ob_p}$, the differentiation of $\mathrm{Ob_p}$ into $\mathrm{Ob_a}$ and the proliferation of $\mathrm{Ob_p}$ at a rate $\mathcal{P}_{\mathrm{Ob_p}}$ which is determined by $P_{\mathrm{Ob_p}}$ and the Wnt–Scl–LRP5/6 signalling pathway through $\pi_{\mathrm{act,Ob_p}}^{\mathrm{Wnt}}$ (see Eq. (88)). As discussed in Buenzli et al. [3], a necessary condition for the $\mathrm{Ob_p}$ population to stay bounded and to converge to a meaningful steady-state (with finite, positive cell densities) is that:

$$\mathcal{P}_{\mathrm{Ob_p}} - \mathcal{D}_{\mathrm{Ob_p}} < 0 \quad \text{as} \;\; t \longrightarrow \infty \tag{90}$$

Following Buenzli et al. [3] $P_{\mathrm{Ob_p}}$ was defined considering a saturation factor:

$$P_{\mathrm{Ob_p}} = \begin{cases} P^0_{\mathrm{Ob_p}} \left(1 - \dfrac{\mathrm{Ob_p}}{\mathrm{Ob_p^{sat}}}\right) & \text{if} \quad \mathrm{Ob_p} < \mathrm{Ob_p^{sat}} \\ 0 & \text{if} \quad \mathrm{Ob_p} \geq \mathrm{Ob_p^{sat}} \end{cases} \tag{91}$$

where $P^0_{\mathrm{Ob_p}}$ is a constant and $\mathrm{Ob_p^{sat}}$ is the maximum concentration of osteoblast precursors above which no proliferation occurs. This saturation and the choice of $P^0_{\mathrm{Ob_p}}$ (see Table 1) ensures that Eq. (90) is fulfilled.

3.9. *Algorithm of bone mineralisation*

The mineralisation model used in this work is based on that presented in [24] and implemented in a model which is similar to the present one [29].

That model was a mixture of differential and recursive equations, difficult to implement in a system of ODEs. For this reason, it has been simplified to yield an explicit set of differential equations, explained next.

Bone is made up of a solid bone matrix and pores filled with marrow. A certain representative volume element, V_{RVE}, can be divided into the bone matrix volume, V_{bm}, and the volume of vascular pores, V_{vas}. The bone matrix volume is divided into inorganic (mineral), organic (mainly collagen) and water phases, designated as V_m, V_o and V_w, respectively:

$$V_{RVE} = V_{bm} + V_{vas} = V_m + V_o + V_w + V_{vas} \tag{92}$$

The composition of bone matrix is defined in terms of the volume fractions of the three phases as:

$$v_i = \frac{V_i}{V_{bm}} \quad \forall i = m, o, w \tag{93}$$

Thus, the following condition holds

$$v_m + v_o + v_w = 1 \tag{94}$$

The mineral content is usually measured by the so-called ash fraction, the ratio between mass of mineral (or ash mass) and dry mass (the sum of inorganic and organic mass):

$$\alpha = \frac{m_m}{m_m + m_o} = \frac{\rho_m\, V_m}{\rho_m\, V_m + \rho_o\, V_o} = \frac{\rho_m\, v_m}{\rho_m\, v_m + \rho_o\, v_o} \tag{95}$$

where Eq. (93) have been used and ρ_i are the corresponding densities of the three phases, being $\rho_m = 3.2\,\mathrm{g/cm^3}$ [7] and $\rho_o = 1.41\,\mathrm{g/cm^3}$ [14]. The tissue density is then given by:

$$\rho_t = \frac{m_w + m_m + m_o}{V_{bm}} = \rho_w\, v_w + \rho_m\, v_m + \rho_o\, v_o = 1 + (\rho_o - 1)\, v_o + (\rho_m - 1)\, v_m \tag{96}$$

where $\rho_w = 1\,\mathrm{g/cm^3}$ and Eqs. (93) and (94) have been used to derive the right-hand side of Eq. (96).

Osteoid, the tissue laid by osteoblasts, contains only the organic phase and no mineral. Mineral accumulates in bone matrix afterwards, during the mineralisation process, which consists of three phases: (i) an initial phase, called mineralisation lag time, that lasts from 6 to 22 days [8, 31] during which no deposition of mineral occurs; (ii) a primary phase, which is very quick (it takes a few days to reach the 70% of the maximum mineral content [13]), and (iii) a secondary phase, when mineral is added at a decreasing rate (Parfitt, 1983), as the tissue becomes saturated with mineral. Mineral accumulates in bone matrix by displacing water [14]. Thus, the volume fraction of organic phase is approximately constant during the mineralisation process and fixed here at $v_o = 3/7$ [23]; while the variations of mineral and water volume fractions would hold $\Delta v_m = -\Delta v_w$. So, the mineralisation process is accounted for through the temporal variation of v_m. As $v_m = \frac{V_m}{V_{bm}}$ (recall Eq. (93)), the temporal derivative of this expression gives:

$$\dot{v}_m = \frac{\dot{V}_m}{V_{bm}} - v_m \frac{\dot{V}_{bm}}{V_{bm}} = \dot{v}_m\Big)_{V_{bm}=constant} - v_m \frac{\dot{V}_{bm}}{V_{bm}} \tag{97}$$

The first term corresponds to the variation of the mineral content due to mineralisation or resorption and it would be equal to the variation of mineral content if V_{bm} were constant. The second term is due to the variation of porosity. To understand this term it must be noted that the mineral content decreases when osteoid is deposited, since osteoid does not contain any mineral and it only contributes to increase the bone matrix volume, so reducing the concentration of mineral. Both terms will be analysed separately:

$$\dot{v}_m\Big)_{V_{bm}=cte} = \dot{v}_m\Big)_{mineralisation} - \dot{v}_m\Big)_{resorption} \tag{98}$$

In a previous model [24, 29] the variation of v_m due to mineralisation was defined in a piecewise manner, considering the three phases separately: with no variation of v_m during the mineralisation lag time, a linear increase during the primary phase and an exponential increase during the secondary phase. Here, as in Martínez-Reina et al. [25], this procedure will be simplified by assuming that it is governed by a saturation model:

$$\dot{v}_m\Big)_{mineralisation} = K\,(v_m^{max} - v_m) \tag{99}$$

leading to an exponential solution which approximates rather well the global response of the previous model for a value of the constant $K = 0.007$, with a fast initial mineralisation rate (primary phase) that slows down in the mid-long-term (secondary phase). The maximum mineral content $v_m^{max} = 0.516$ was fixed such that, together with the aforementioned $v_o = 3/7$, it yields the maximum tissue density $\rho_t^{max} = 2.31\,\text{g/cm}^3$ [14] through Eq. (96). The rate constant K will be assumed fixed in this work, though it may depend on the amount of calcium and phosphorus available in the serum, which, in turn, may depend on diverse physiological factors [34].

The variation of mineral content due to resorption is similar to the damage repair term in the damage model (see Eq. (78)). It must be taken into account that mineral is dissolved by osteoclasts and so removed from the bone matrix as damage was previously assumed. Thus, the amount of mineral removed by resorption is proportional to the mineral content of the tissue being resorbed, v_m, and to the proportion of tissue being resorbed within the bone matrix, $\frac{\dot{V}_r}{V_{bm}}$:

$$\dot{v}_m\Big)_{resorption} = v_m\,\frac{\dot{V}_r}{V_{bm}} = v_m\,\frac{\dot{V}_r/V_{RVE}}{V_{bm}/V_{RVE}} = v_m\,\frac{\dot{V}_r/V_{RVE}}{f_{bm}} = \frac{v_m}{f_{bm}}\,k_{res}\,Oc_a \tag{100}$$

Using Eqs. (98)-(100) Eq. (97) can be rewritten as:

$$\dot{v}_m = K\,(v_m^{max} - v_m) - \frac{v_m}{f_{bm}}\,k_{res}\,Oc_a - v_m\,\frac{\dot{f}_{bm}}{f_{bm}} \tag{101}$$

where it has been used that $\frac{\dot{V}_{bm}}{V_{bm}} = \frac{\dot{f}_{bm}}{f_{bm}}$. Taking into account the balance between formation and resorption (Eq.14 in the main document):

$$\dot{v}_m = K\,(v_m^{max} - v_m) - \frac{v_m}{f_{bm}}\,k_{form}\,Ob_a \tag{102}$$

Once v_m is updated, the ash fraction can be derived from Eq. (95) and the tissue density from Eq. (96). Then, the apparent density is given by:

$$\rho = \frac{m_w + m_m + m_o}{V_{RVE}} = \frac{m_w + m_m + m_o}{V_{bm}} \frac{V_{bm}}{V_{RVE}} = \rho_t \, f_{bm} \tag{103}$$

Bone was assumed to be an isotropic material with a Poisson's ratio $\nu = 0.3$ and a Young's modulus given in MPa by the following expresions:

$$E(\rho, d) = \begin{cases} 2014 \, \rho^{2.5} \, (1-d) & \text{if} \;\; \rho < 1.2 \; g/cm^3 \\ 1763 \, \rho^{3.2} \, (1-d) & \text{if} \;\; \rho \geq 1.2 \; g/cm^3 \end{cases} \tag{104}$$

These are based on the correlations experimentally obtained by Jacobs [15], which were multiplied by the factor $(1-d)$ to consider microstructural damage as usually done in Continuum Damage Mechanics [20].

3.10. Pharmacodynamic model of alendronate

This BCPM was integrated into the PK model by including it in the bone compartment (BC, see Fig. 2). All the absorption rate constants k_i and the elimination rate constants $k_{el,i}$ fitted for the PK model (see Fig. 2) were assumed constant, except for $k_{el,BC}$, which is variable and depends on the osteoclast-mediated release of alendronate from bone matrix. Note that the latter variable depends on bone turnover [45, 9], whereas in the PK model of section 2 $k_{el,BC}$ was constant. Now, in the PK-PD model $k_{el,BC}$ is given by:

$$k_{el,BC} = k_{res} \, Oc_a \tag{105}$$

We have assumed that it is the concentration of alendronate in the bone compartment, BC, that controls the action of the drug on the bone remodelling process:

$$[Ale_{BC}] = \frac{Ale_{BC}}{V_{Bone}} \tag{106}$$

where V_{Bone} is the total volume of bone tissue in the body, $2.23 \cdot 10^{-3} m^3$, adapted from [43] for a 60kg adult female. The amount of alendronate accumulated within the bone increases with each weekly dose and therefore, if the effect of the drug were proportional to that amount, such effect would be increasingly pronounced. But this is not observed in the clinical trials

[39], where the increase of BMD is high during the first months after the beginning of the treatment and slows down in the subsequent months, both in the lumbar spine and the hip. This behaviour could be explained by several facts highlighted in the literature:

- Alendronate is preferentially deposited in areas undergoing active resorption [38].
- 80% of normal bone turnover occurs in trabecular bone and 20% in cortical bone [10].
- A considerably higher amount of alendronate is deposited in trabecular bone compared to cortical bone [21, 19].
- The volume of cortical bone is much higher than that of trabecular bone [10, 43].

Therefore, a higher proportion of alendronate would be initially deposited in trabecular bone, which explains the fast increase of BMD in the lumbar spine and the hip observed in the clinical results. However, due to the fact that the volume of trabecular bone is much lower than that of cortical bone, in a long treatment it is expected that the proportion of alendronate that reaches the former will be reduced over time in favour of the latter, due to the saturation of the tissue. This would explain the long-term stabilisation of bone mass gain observed clinically.

In view of the clinical results, we hypothesized that the BC can be divided in two parts: one part termed active, which is the closest to the bone-marrow interface and where the retrieval of alendronate is immediate through bone resorption; the second part, termed inactive, corresponds to the innermost regions of bone, where alendronate is buried and, to some extent, inaccessible to bone resorption [40, 38]. An updated compartmental model is shown in Fig. 3. The active subcompartment would predominate in trabecular bone, where most of the tissue is superficial, and its proportion would decrease with porosity, as the tissue becomes more compact.

We need a variable to measure this compacity, i.e. the proportion of superficial tissue. The specific surface, S_v, is defined as the area of bone matrix-marrow interface, S_i, per total volume of the bone sample, V_T. The following correlation between S_v and vascular porosity, f_{vas}, was given by [23] and used by [36]:

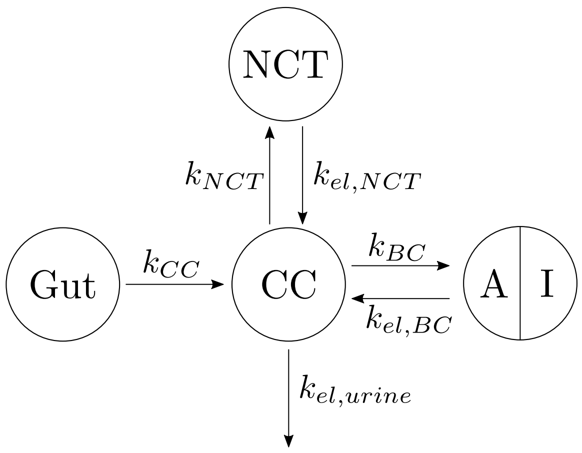

Figure 3: Three-compartment model "in parallel", adapted with the division of the BC compartment into an active (A) and an inactive (I) subcompartment.

$$S_v = \frac{S_i}{V_T} = 32.3 \cdot f_{vas} - 93.9 \cdot f_{vas}^2 + 134 \cdot f_{vas}^3 - 101 \cdot f_{vas}^4 + 28.8 \cdot f_{vas}^5 \tag{107}$$

where the vascular porosity is $f_{vas} = 1 - \frac{f_{bm}}{100}$ and S_v is expressed in mm^2/mm^3, being this variable equal to 0 both for $f_{vas} = 0$ and $f_{vas} = 1$, when no free surface exists. In order to measure the compacity of the tissue, we would need to express that specific surface per bone matrix volume instead of per total volume:

$$\frac{S_i}{V_b} = \frac{\frac{S_i}{V_T}}{\frac{V_b}{V_T}} = \frac{S_v}{f_{bm}} \tag{108}$$

This quotient is not defined for $f_{bm} = 0$, but Eq.(108) is not needed for very low values of f_{bm}, as we can assume that all the tissue is superficial and accessible to bone resorption in a very porous bone. More precisely, we have established $f_{bm0} = 5\%$ as the minimum bone matrix below which all the tissue is considered superficial. We have normalized the quotient in Eq.(108) and used the following expression for the active alendronate, i.e. the amount of alendronate contained in the active subcompartment:

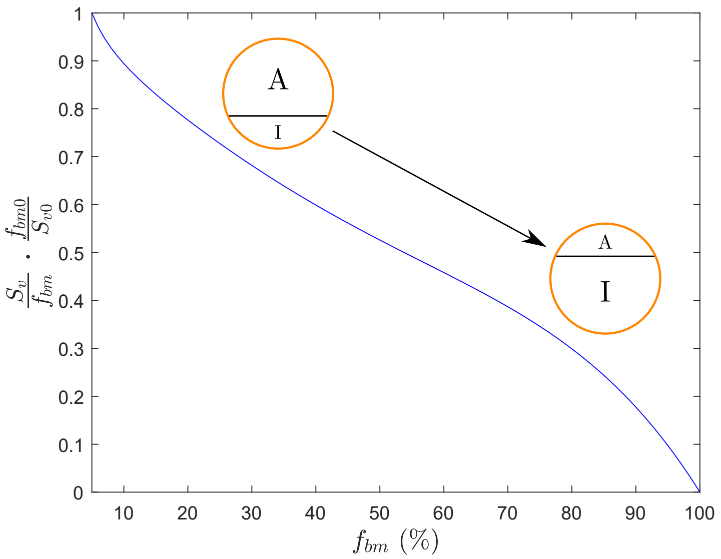

Figure 4: Function that regulates the division between the active (A) and inactive (I) parts of BC.

$$Ale_{BC,act} = Ale_{BC} \cdot \frac{S_v}{f_{bm}} \cdot \frac{f_{bm0}}{S_{v0}} \tag{109}$$

where S_{v0} is the specific area corresponding to the reference value f_{bm0}. The concentration of active alendronate can be assessed as in Eq.(106), i.e. $[Ale_{BC,act}] = \frac{Ale_{BC,act}}{V_{bone}}$. The function $\frac{S_v}{f_{bm}} \cdot \frac{f_{bm0}}{S_{v0}}$, that controls the division between the active and inactive parts of the BC, is plotted against f_{bm} in Fig. 4. If the tissue has no pores ($f_{bm} = 100\%$), all the BC is inactive as there is no surface where osteoclasts can resorb bone and, thus, there is no release of alendronate from bone matrix. As porosity increases (f_{bm} decreases), the ratio between free surface and bone volume rises, as does the exposure of the drug (the active subcompartment becomes predominant). In contrast, if more tissue is formed than resorbed, f_{bm} increases and the inactive part becomes predominant, as the alendronate that was on the surface is buried into the bone matrix and is thus less accessible to osteoclasts.

So, Eqs. (14) and (15) are rewritten as follows, by changing the second terms on the right-hand side of both:

$$\frac{dAle_{CC}}{dt} = F \cdot k_{CC} Ale_{Gut} + k_{el,BC}[Ale_{BC,act}] \frac{V_{Bone}}{\frac{f_{bm}^{aver}}{100}} + k_{el,NCT} Ale_{NCT} - (k_{BC} + k_{NCT} + k_{el,urine}) Ale_{CC} \tag{110}$$

$$\frac{dAle_{BC}}{dt} = k_{BC} Ale_{CC} - k_{el,BC}[Ale_{BC,act}] \frac{V_{Bone}}{\frac{f_{bm}^{aver}}{100}} \tag{111}$$

Since $[Ale_{BC,act}]$ is the local concentration, $k_{el,BC}[Ale_{BC,act}]$ represents the local amount of alendronate per unit volume or the RVE, V_{RVE}, released from bone matrix through resorption. As the flux from BC to CC is systemic, one must consider the contribution of all the V_{RVE} in the skeleton, which is done in a simplified way through the factor $V_{Bone}/(f_{bm}^{aver}/100)$. There, $f_{bm}^{aver}/100$ represents an average bone volume fraction of the skeleton, that allows to express the amount of alendronate per unit volume of bone tissue (recall Eq.(30)), which is then multiplied by the total volume of bone tissue in the skeleton, V_{Bone}. Eqs.(110) and (111) imply that the systemic flux from BC to CC is proportional to the flux from the local RVE to the CC and if bone turnover changes locally for any reason it will change accordingly in the whole skeleton. The adopted value $f_{bm}^{aver} = 43.7\%$ was estimated by assuming an average $f_{bm} = 93\%$ for cortical bone [4], $f_{bm} = 14\%$ for trabecular one [42] and that 80% of bone tissue volume is cortical and the rest is trabecular [43].

The active subcompartment is predominant in trabecular bone whose volume is considerably lower than that of cortical bone. Thus, it is plausible to assume that this subcompartment will become saturated and relatively soon, whereas the total alendronate in the BC will not, as indicated in the previous subsection, because there is a large volume of cortical bone in the body able to admit high doses of the drug. It could also become saturated in cases of very long treatments and then the excess of alendronate would be eliminated via urine, but the authors found no information on urine excretion in long treatments. The active alendronate has been saturated also as a function of S_v/f_{bm}, being its maximum concentration:

$$[Ale_{BC,act}]^{max} = f \cdot \frac{S_v}{f_{bm}} \cdot \frac{f_{bm0}}{S_{v0}} \tag{112}$$

with f a constant to be fitted. Thus, as alendronate enters the BC (through Eq.(111)), it is divided between the active and inactive subcompartments

following Eq.(109)[2] until $[Ale_{BC,act}] = [Ale_{BC,act}]^{max}$, when the active subcompartment becomes saturated and no more drug is allowed in it.

Alendronate has the following two effects on bone cells. On the one hand, it causes the disappearance of clear zones and ruffled borders disrupting the cytoskeleton of osteoclasts by inhibiting farnesyl pyrophosphate (FPP) synthase, which leads to these structural changes and loss of function [41, 12]. In other words, it limits the resorbing capacity of osteoclasts, which is measured in the model by k_{res} (see Eq.(31)). Consequently, this parameter is reduced as follows:

$$k_{res} = k_{res,nom}\left(1 - \Pi_{rep}^{Ale} \cdot k_{el,BC} \cdot [Ale_{BC,act}]\right) \tag{113}$$

where $k_{res,nom}$ is the nominal value of k_{res}, Π_{rep}^{Ale} is a constant quantifying the effect of alendronate on the resorbing capacity of osteoclasts and $k_{el,BC} \cdot [Ale_{BC,act}]$ is the amount of alendronate per unit volume released from bone matrix through resorption, i.e. the concentration of alendronate that affects the surrounding osteoclasts. If Eq.(105) is replaced in (113):

$$k_{res} = k_{res,nom}\left(1 - \Pi_{rep}^{Ale} \cdot k_{res} \cdot Oc_a \cdot [Ale_{BC,act}]\right) \tag{114}$$

from which k_{res} can be worked out:

$$k_{res} = \frac{k_{res,nom}}{1 + \Pi_{rep}^{Ale} \cdot k_{res,nom} \cdot Oc_a \cdot [Ale_{BC,act}]} \tag{115}$$

The second effect caused by alendronate is the inhibition of the FPP synthase in the mevalonate pathway and the reduction of protein prenylation, an essential post-translational lipid modification required for the function of numerous proteins, thereby inducing apoptosis in osteoclasts [45, 41, 12, 40]. To account for this effect the apoptosis rate was increased in the model as follows:

$$A_{Oc_a} = A_{Oc_a,nom}\left(1 + \Pi_{act}^{Ale} \cdot k_{el,BC} \cdot [Ale_{BC,act}]\right) \tag{116}$$

[2]The rest goes to the inactive subcompartment, i.e. $Ale_{BC,inact} = Ale_{BC} - Ale_{BC,act}$.

where Π_{act}^{Ale} quantifies the effect of alendronate on the apoptosis of osteoclasts. If Eq.(105) is replaced in (116):

$$A_{Oc_a} = A_{Oc_a,nom}\left(1 + \Pi_{act}^{Ale} \cdot k_{res}Oc_a \cdot [Ale_{BC,act}]\right) \tag{117}$$

3.11. Model constants

The model constants, except for those related to the damage and mineralisation algorithms, which were given in their respective sections, and for those adjusted in this work, which were given in the main document in the result section, are provided in the following table.

Constant	Value	Units
Cell constants: differentiation, proliferation, apoptosis, activity		
$\mathrm{Ob_u}$	0.01	pM
$\mathrm{Oc_u}$	0.01	pM
$D_{\mathrm{Ob_u}}$	0.083	day^{-1}
$D_{\mathrm{Ob_p}}$	0.185	day^{-1}
$P^0_{\mathrm{Ob_p}}$	2.73	day^{-1}
$\mathrm{Ob_p^{sat}}$	0.005	pM
$D_{\mathrm{Oc_u}}$	0.011	day^{-1}
$D_{\mathrm{Oc_p}}$	0.0196	day^{-1}
$\Delta_{\mathrm{Ob_a}}$	0.212	day^{-1}
$A_{\mathrm{Oc_a}}$	10.0	day^{-1}
η	$4.143 \cdot 10^{-4}$	pM / %[3]
k_{res}	2500	% day^{-1} pM^{-1}
k_{form}	50	% day^{-1} pM^{-1}
RANK-RANKL-OPG signalling pathway		
$\tilde{D}_{\mathrm{OPG}}$	$5.326 \cdot 10^{5}$	day^{-1}
$\tilde{D}_{\mathrm{RANKL}}$	10.132	day^{-1}
$\tilde{D}_{\mathrm{OPG-RANKL}}$	10.132	day^{-1}
$\tilde{D}_{\mathrm{RANK-RANKL}}$	10.132	day^{-1}
$K_{\mathrm{OPG-RANKL}}$	$1.511 \cdot 10^{-2}$	pM
$K_{\mathrm{RANK-RANKL}}$	10	pM
$N^{\mathrm{RANK}}_{\mathrm{Oc_p}}$	$4.16 \cdot 10^{3}$	pM RANK / pM cell
$\beta_{\mathrm{OPG,Ob_a}}$	$1.625 \cdot 10^{8}$	pM OPG / pM cell day^{-1}

Continued on next page

[3] Recall that f_{bm} is expressed in %.

Constant	Value	Units
$[\mathrm{OPG}]_{\mathrm{max}}$	$1.314 \cdot 10^2$	pM
$[\mathrm{RANKL}]_{\mathrm{max}}$	$3.051 \cdot 10^3$	pM
$\beta_{\mathrm{RANKL,Ot}}$	$5.66 \cdot 10^3$	pM RANKL / pM cell day^{-1}
$\beta_{\mathrm{RANKL,Ob_p}}$	$2.36 \cdot 10^4$	pM RANKL / pM cell day^{-1}
$K^{\mathrm{RANKL}}_{\mathrm{act,Oc_u}}$	16.7	pM
$K^{\mathrm{RANKL}}_{\mathrm{act,Oc_p}}$	3.34	pM
	Upregulation of RANKL via damage	
ρ_{dam}	0.04	-
α_{dam}	1	-
K_{dam}	0.15	-
	Competitive binding Wnt-Scl-LRP5/6	
$\tilde{D}^{0}_{\mathrm{Scl}}$	1	day^{-1}
$\tilde{D}_{\mathrm{Scl-LRP5/6}}$	50	day^{-1}
$K_{\mathrm{Wnt-LRP5/6}}$	1000	pM
$K_{\mathrm{Scl-LRP5/6}}$	10	pM
$N^{\mathrm{LRP5/6}}_{\mathrm{OB_p}}$	5	pM LRP5/6 / pM cell
$\beta_{\mathrm{Scl,Ot}}$	$2.4 \cdot 10^4$	pM Scl / pM cell day^{-1}
$[\mathrm{Wnt}]$	170	pM
$[\mathrm{Scl}]_{\mathrm{max}}$	70	pM
$P_{\mathrm{Scl,d}}$	0	pM day^{-1}
	Co-regulation of RANKL via PTH and NO	
λ_s	0.45	-
λ_c	0.9	-
$K^{\mathrm{PTH}}_{\mathrm{act}}$	0.65	pM
$K^{\mathrm{PTH}}_{\mathrm{rep}}$	0.223	pM
$K^{\mathrm{NO}}_{\mathrm{rep}}$	$6.44 \cdot 10^3$	pM
$[\mathrm{NO}]_{\mathrm{max}}$	$2 \cdot 10^8$	pM
β_{PTH}	250	pM day $^{-1}$
$\tilde{D}_{\mathrm{PTH}}$	86	day^{-1}
$\beta_{\mathrm{NO,Ot}}$	$3.44 \cdot 10^3$	pM NO / pM cell day^{-1}

Continued on next page

Constant	Value	Units
$\tilde{D}_{\mathrm{NO}}$	$2.1 \cdot 10^{-3}$	day^{-1}
$P_{\mathrm{NO,d}}$	0	pM day^{-1}
PMO related constants		
$P_{RANKL}^{PMO,max}$	8	pM
γ	0.8	-
δ_{PMO}	300	days
τ_{PMO}	20	years
TGF-β related constants		
$\frac{\alpha_{\mathrm{TGF}-\beta}\, k_{res}}{\tilde{D}_{\mathrm{TGF}-\beta}}$	1	-
$K_{\mathrm{act}}^{\mathrm{TGF}-\beta}$	$5.633 \cdot 10^{-4}$	pM
$K_{\mathrm{rep}}^{\mathrm{TGF}-\beta}$	$1.754 \cdot 10^{-4}$	pM
Parameters of mechanical regulation		
α_{rep}	1	-
α_{act}	1	-
ρ_{rep}	0	-
ρ_{act}	0	-
δ_{rep}	$9.612 \cdot 10^{-3}$	MPa
δ_{act}	$4.368 \cdot 10^{-3}$	MPa
γ_{rep}	8	-
γ_{act}	7	-
Alendronate		
k_{CC}	16.33	day^{-1}
$k_{el,urine}$	14.80	day^{-1}
k_{NCT}	6.81	day^{-1}
$k_{el,NCT}$	$4.90 \cdot 10^{-2}$	day^{-1}
k_{BC}	7.39	day^{-1}
V_c	3.84	L
F	$5.36 \cdot 10^{-3}$	-
Π_{act}^{Ale}	12.98	day/mM

Continued on next page

Constant	Value	Units
Π_{rep}^{Ale}	14.31	day/mM
f	$7.72 \cdot 10^{-2}$	day/mM

Table 1: Values taken for the constants of the PK-PD model

4. Sensitivity analysis

In this section, the figures corresponding to the variation of each constant by 10% are presented.

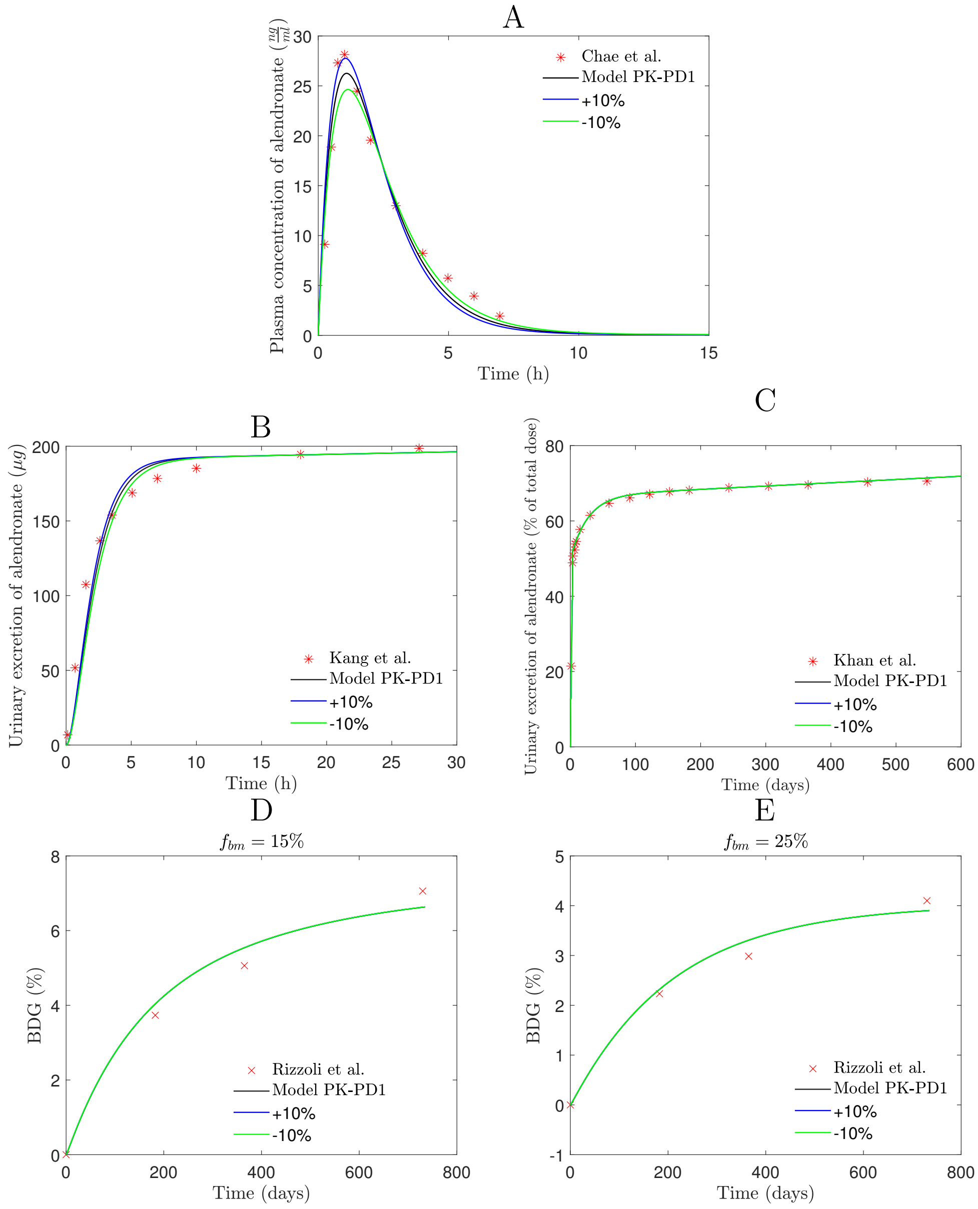

Figure 5: Sensitivity analysis for a ±10% variation of k_{CC}. A: Alendronate plasma concentration vs time; B: Alendronate short-term urinary excretion vs time; C: Alendronate long-term urinary excretion vs time; D: BDG vs time for hip, i.e. $f_{bm} = 15\%$ and E: BDG vs time for lumbar vertebra, i.e. $f_{bm} = 25\%$, for a once-weekly 70 mg dose. In images C, D and E the three curves are practically overlapping.

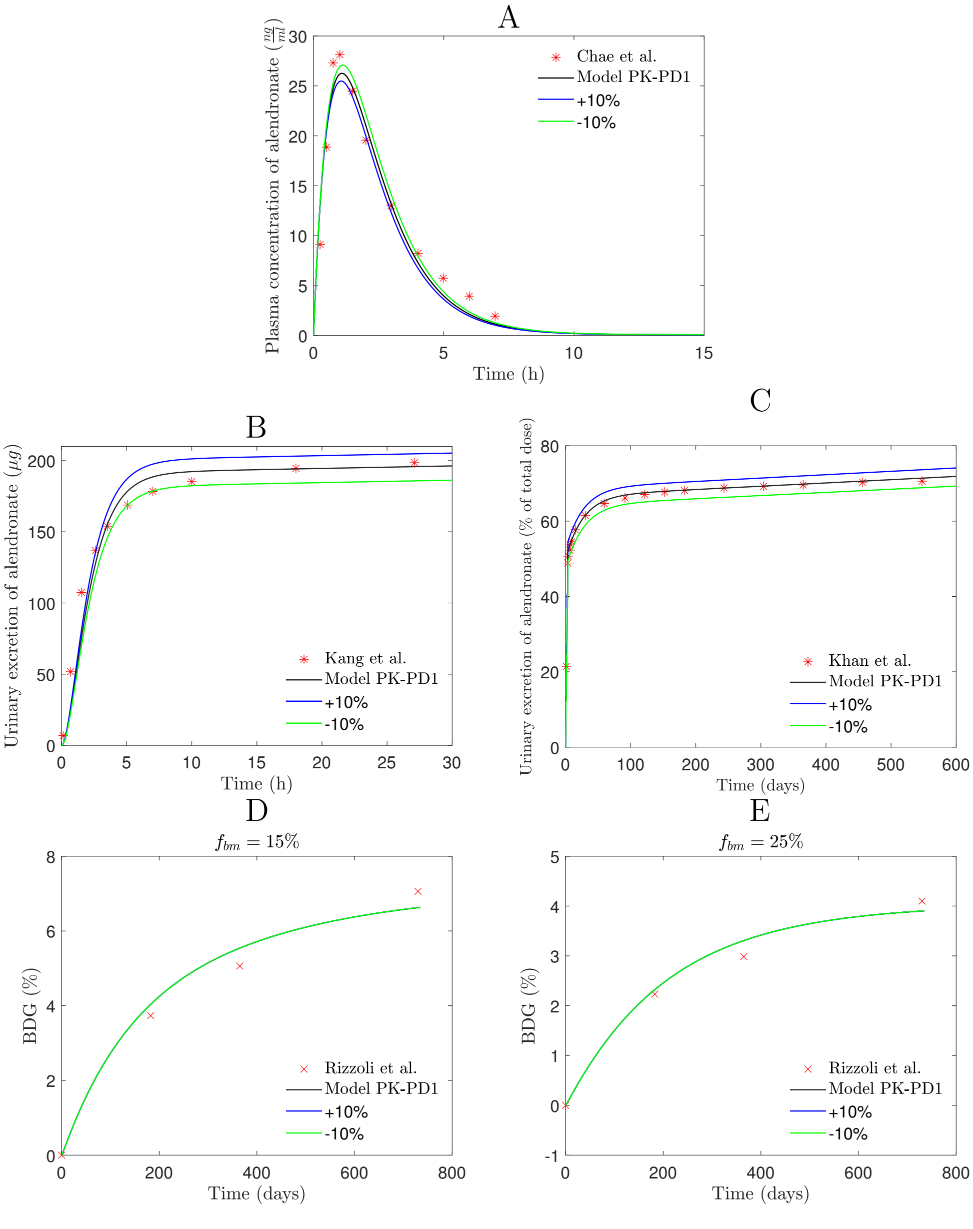

Figure 6: Sensitivity analysis for a $\pm10\%$ variation of $k_{el,urine}$. A: Alendronate plasma concentration vs time; B: Alendronate short-term urinary excretion vs time; C: Alendronate long-term urinary excretion vs time; D: BDG vs time for hip, i.e. $f_{bm} = 15\%$ and E: BDG vs time for lumbar vertebra, i.e. $f_{bm} = 25\%$, for a once-weekly 70 mg dose. In images D and E the three curves are practically overlapping.

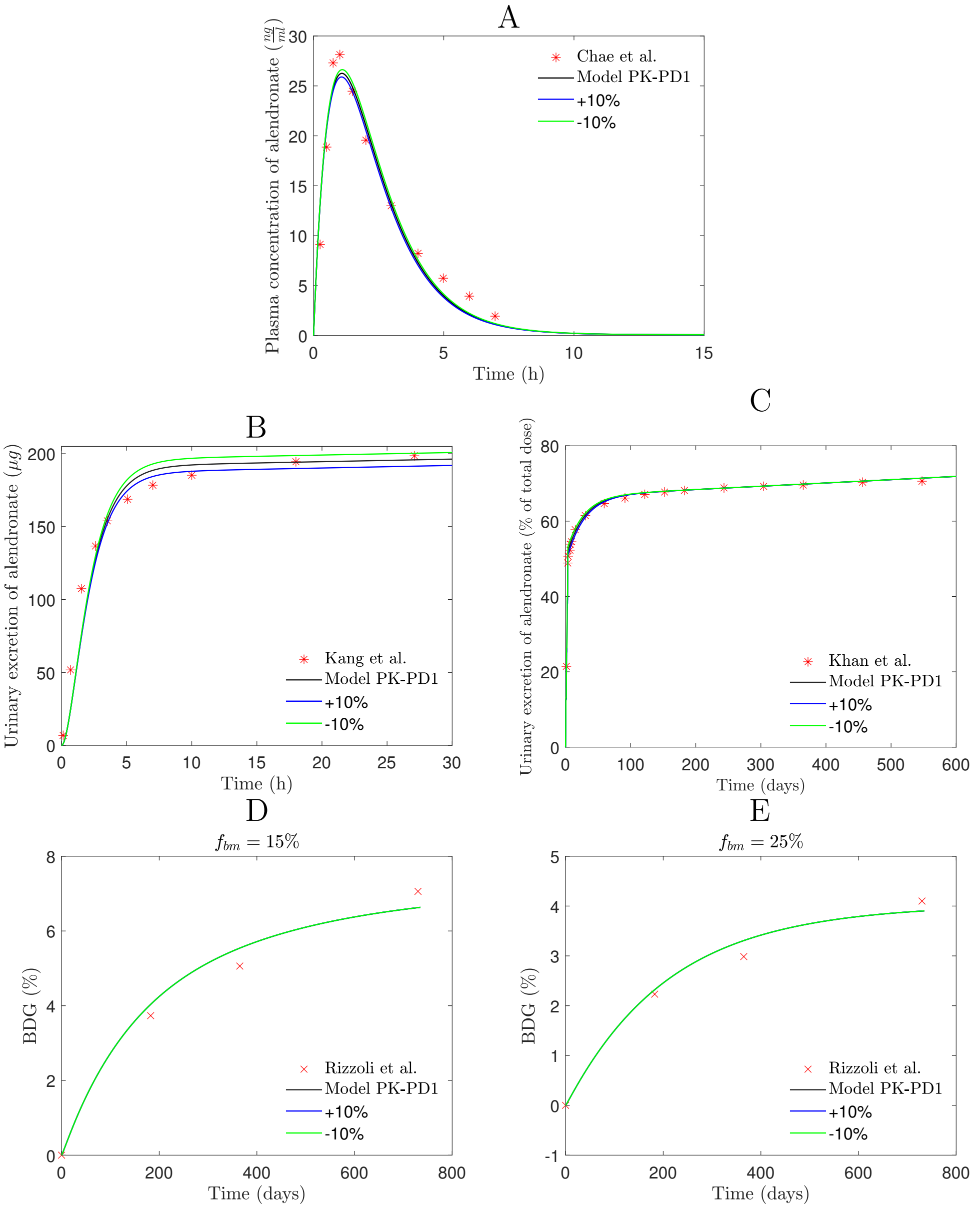

Figure 7: Sensitivity analysis for a $\pm 10\%$ variation of k_{NCT}. A: Alendronate plasma concentration vs time; B: Alendronate short-term urinary excretion vs time; C: Alendronate long-term urinary excretion vs time; D: BDG vs time for hip, i.e. $f_{bm} = 15\%$ and E: BDG vs time for lumbar vertebra, i.e. $f_{bm} = 25\%$, for a once-weekly 70 mg dose. In images D and E the three curves are practically overlapping.

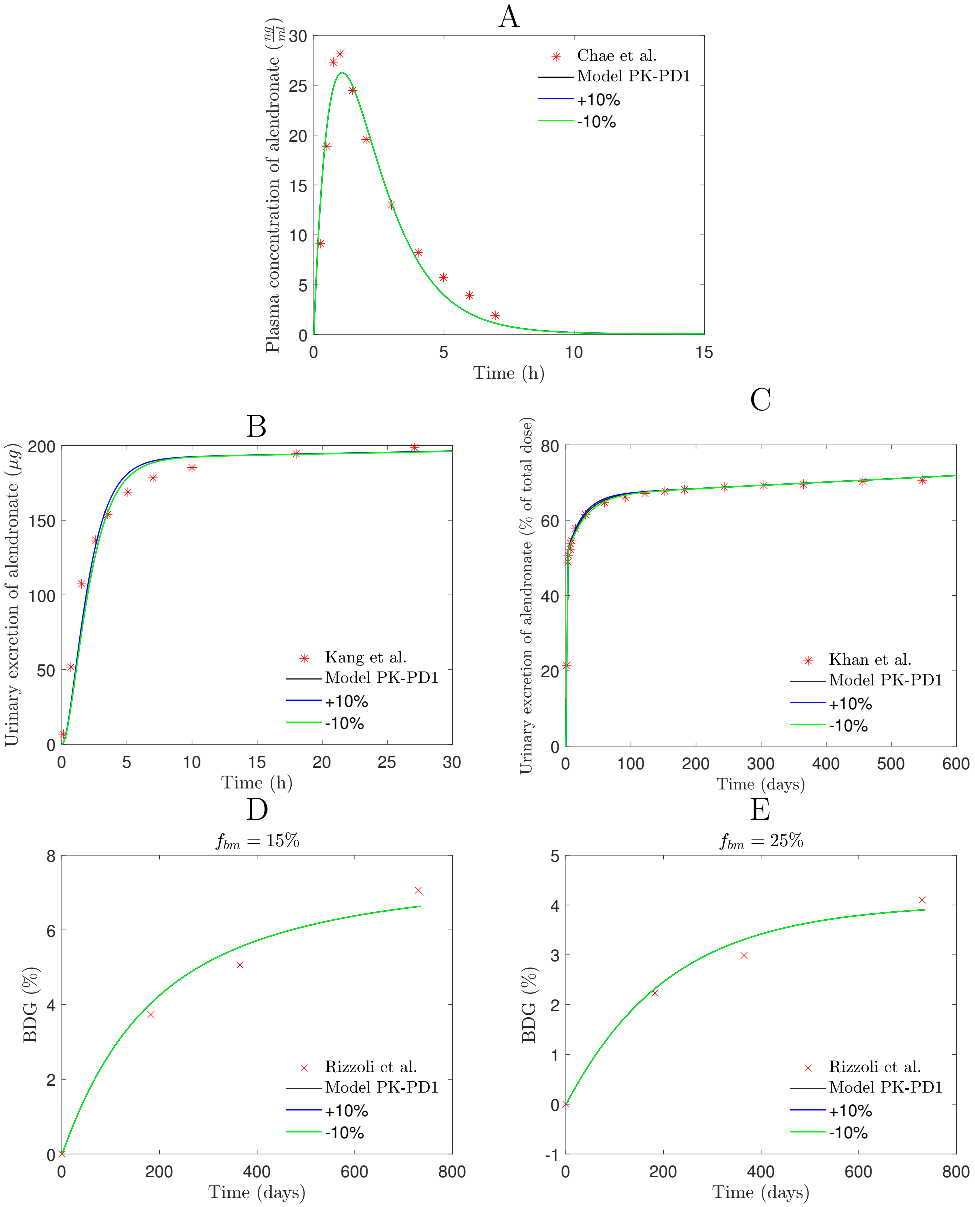

Figure 8: Sensitivity analysis for a ±10% variation of $k_{el,NCT}$. A: Alendronate plasma concentration vs time; B: Alendronate short-term urinary excretion vs time; C: Alendronate long-term urinary excretion vs time; D: BDG vs time for hip, i.e. $f_{bm} = 15\%$ and E: BDG vs time for lumbar vertebra, i.e. $f_{bm} = 25\%$, for a once-weekly 70 mg dose. In images A, D and E the three curves are practically overlapping. In image B, the curves for the fitted constants and the one where the parameters are varied by -10% overlap.

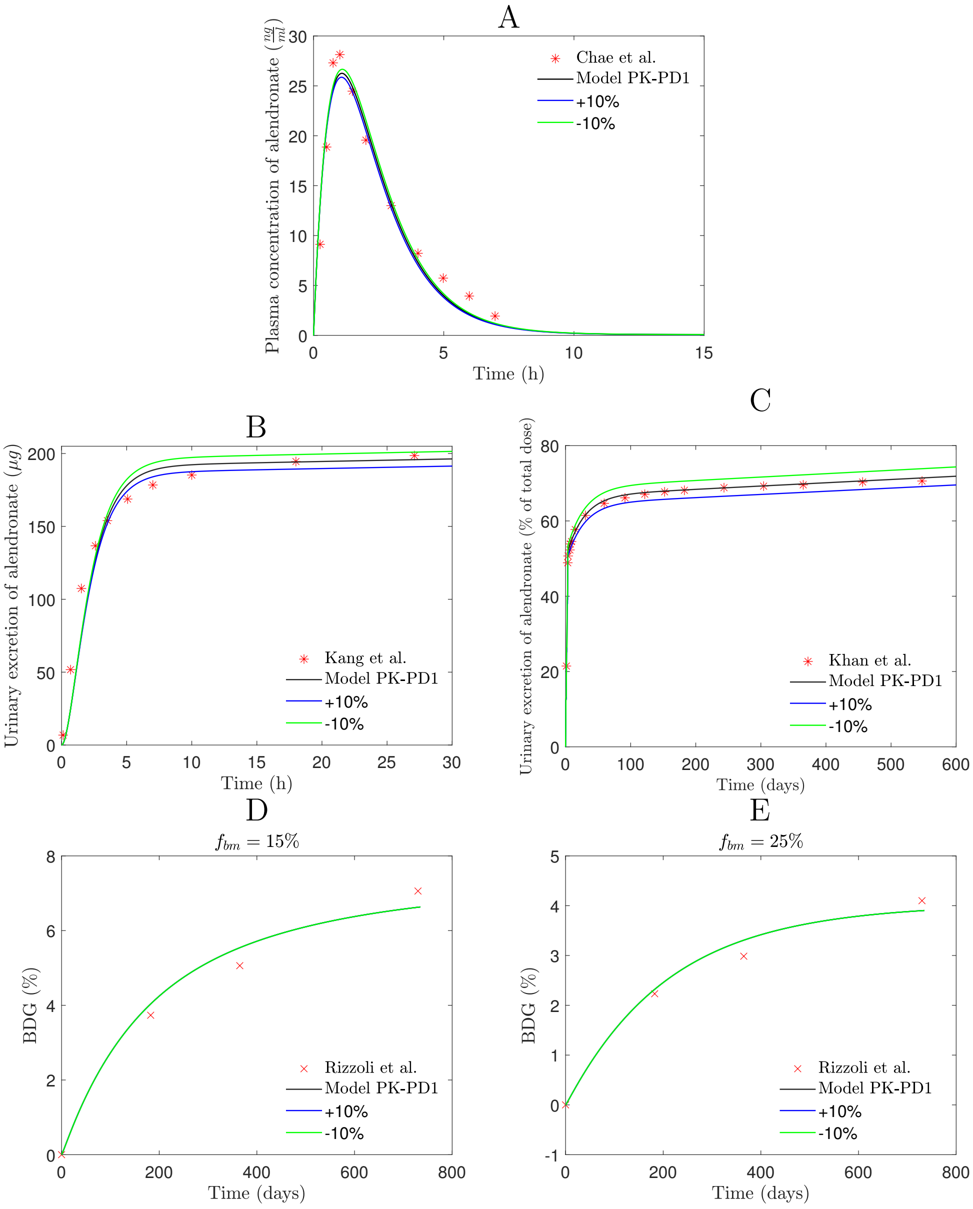

Figure 9: Sensitivity analysis for a $\pm 10\%$ variation of k_{BC}. A: Alendronate plasma concentration vs time; B: Alendronate short-term urinary excretion vs time; C: Alendronate long-term urinary excretion vs time; D: BDG vs time for hip, i.e. $f_{bm} = 15\%$ and E: BDG vs time for lumbar vertebra, i.e. $f_{bm} = 25\%$, for a once-weekly 70 mg dose. In images D and E the three curves are practically overlapping.

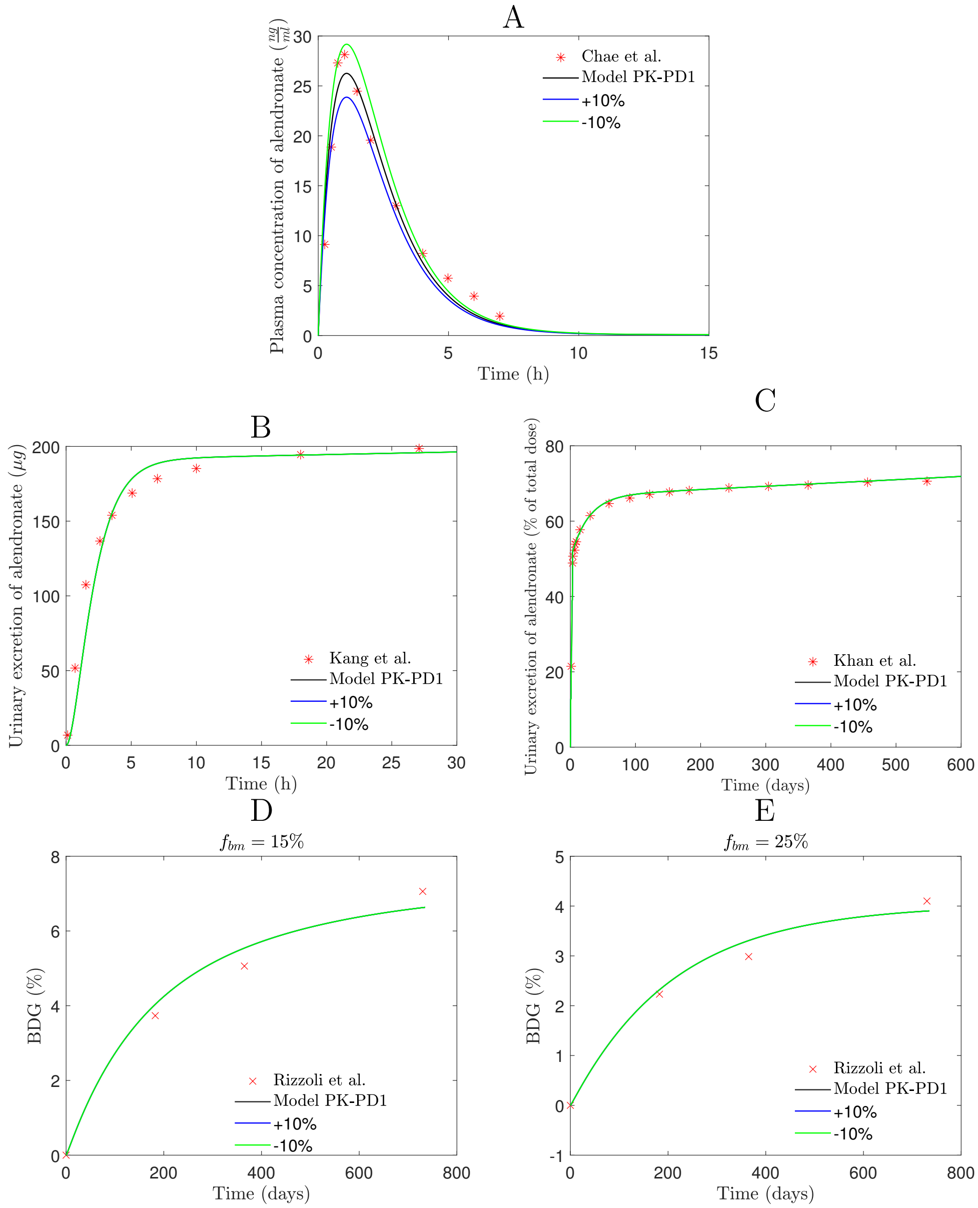

Figure 10: Sensitivity analysis for a ±10% variation of V_c. A: Alendronate plasma concentration vs time; B: Alendronate short-term urinary excretion vs time; C: Alendronate long-term urinary excretion vs time; D: BDG vs time for hip, i.e. $f_{bm} = 15\%$ and E: BDG vs time for lumbar vertebra, i.e. $f_{bm} = 25\%$, for a once-weekly 70 mg dose. In images B, C, D and E the three curves are practically overlapping.

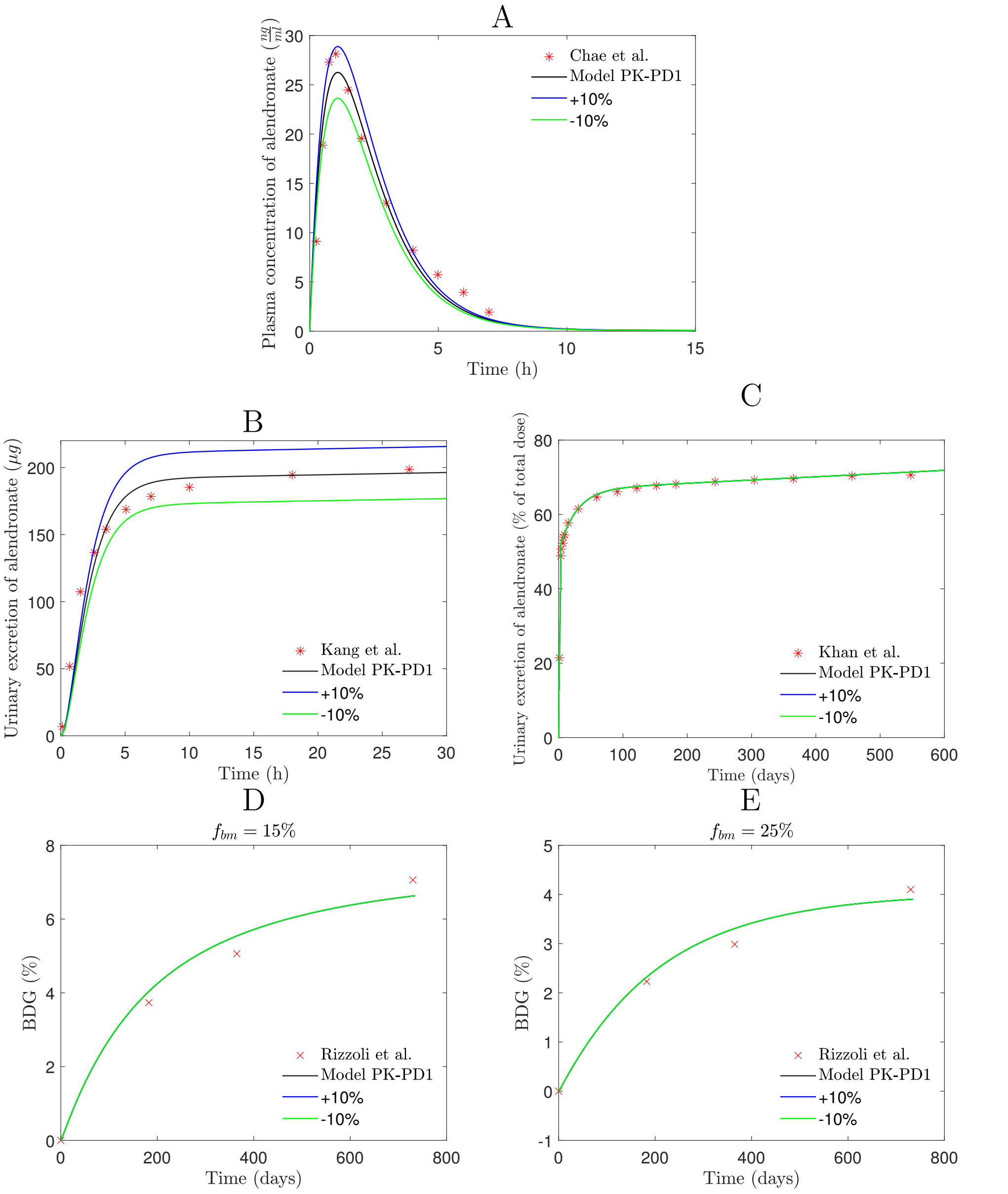

Figure 11: Sensitivity analysis for a $\pm 10\%$ variation of F. A: Alendronate plasma concentration vs time; B: Alendronate short-term urinary excretion vs time; C: Alendronate long-term urinary excretion vs time; D: BDG vs time for hip, i.e. $f_{bm} = 15\%$ and E: BDG vs time for lumbar vertebra, i.e. $f_{bm} = 25\%$, for a once-weekly 70 mg dose. In images C, D and E the three curves are practically overlapping.

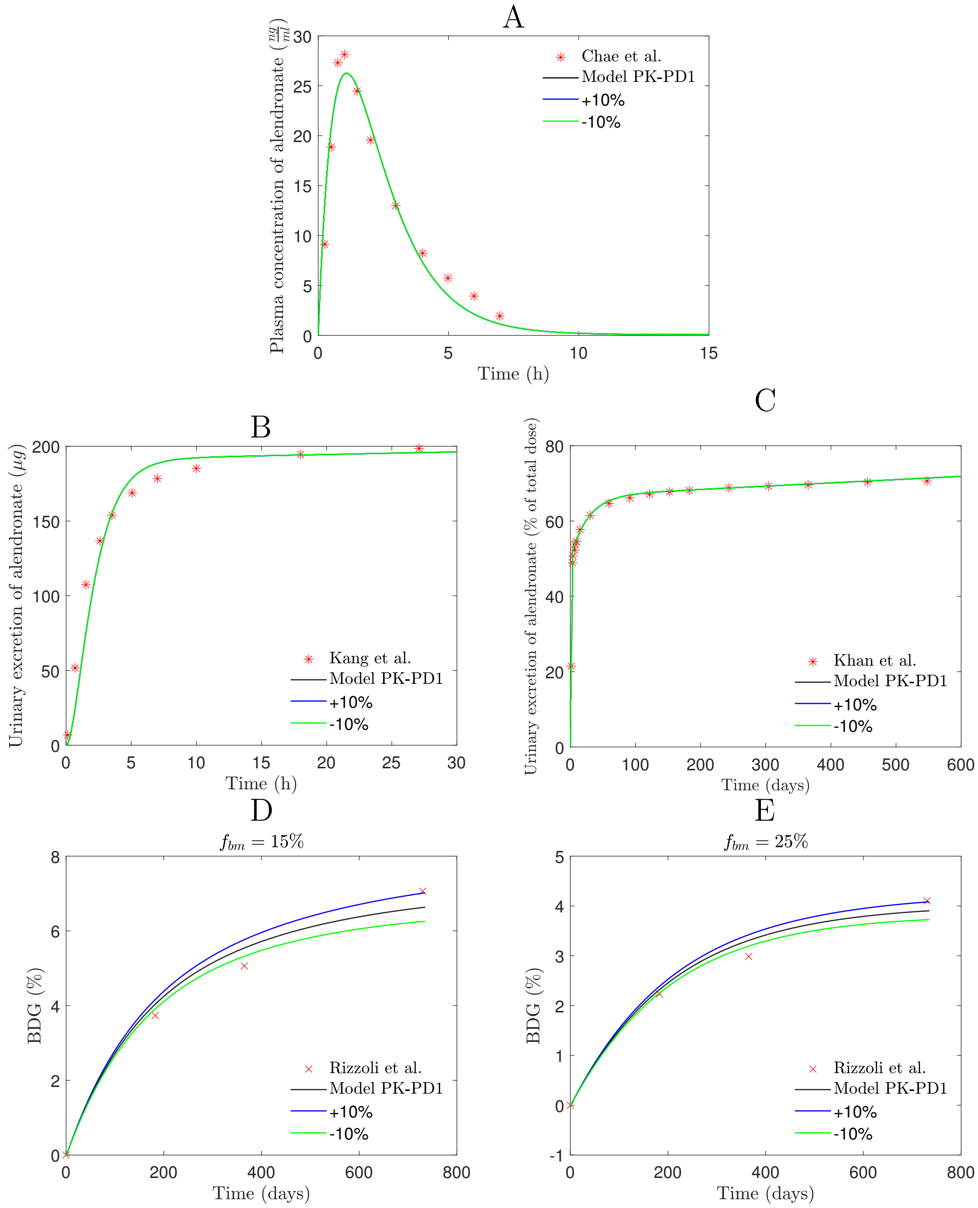

Figure 12: Sensitivity analysis for a $\pm 10\%$ variation of Π_{act}^{Ale}. A: Alendronate plasma concentration vs time; B: Alendronate short-term urinary excretion vs time; C: Alendronate long-term urinary excretion vs time; D: BDG vs time for hip, i.e. $f_{bm} = 15\%$ and E: BDG vs time for lumbar vertebra, i.e. $f_{bm} = 25\%$, for a once-weekly 70 mg dose. In images A, B and C the three curves are practically overlapping.

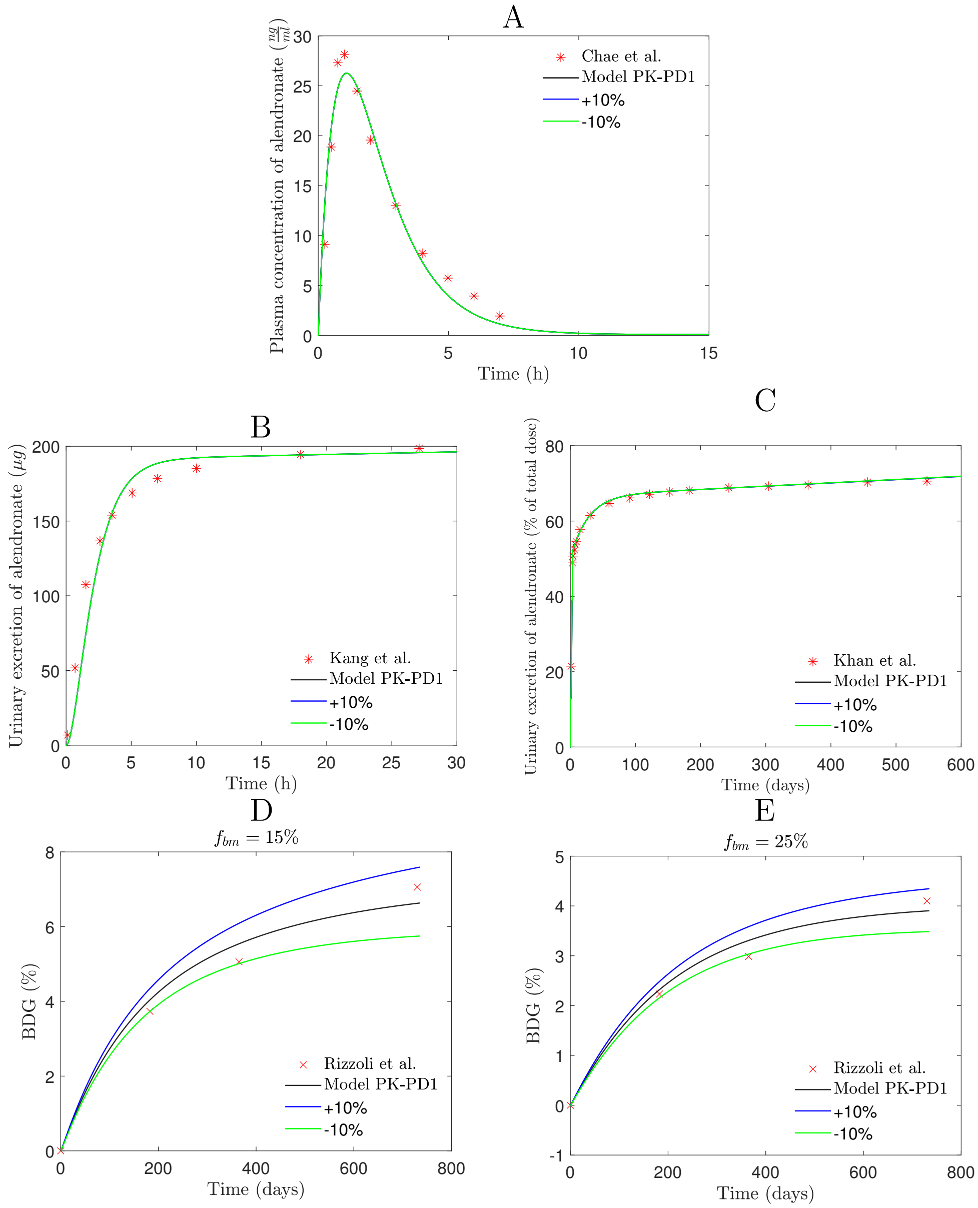

Figure 13: Sensitivity analysis for a ±10% variation of Π_{rep}^{Ale}. A: Alendronate plasma concentration vs time; B: Alendronate short-term urinary excretion vs time; C: Alendronate long-term urinary excretion vs time; D: BDG vs time for hip, i.e. $f_{bm} = 15\%$ and E: BDG vs time for lumbar vertebra, i.e. $f_{bm} = 25\%$, for a once-weekly 70 mg dose. In images A, B and C the three curves are practically overlapping.

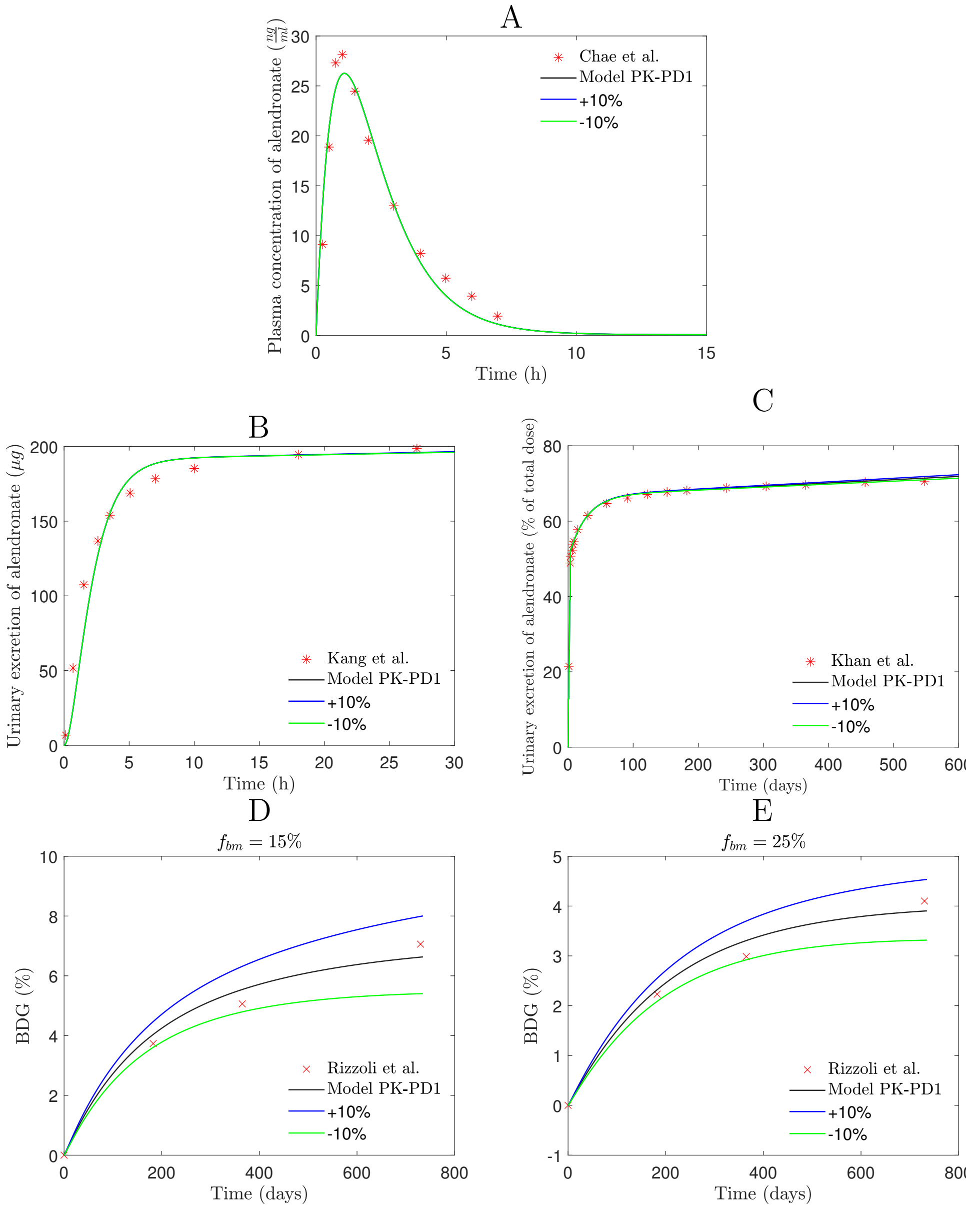

Figure 14: Sensitivity analysis for a ±10% variation of f. A: Alendronate plasma concentration vs time; B: Alendronate short-term urinary excretion vs time; C: Alendronate long-term urinary excretion vs time; D: BDG vs time for hip, i.e. $f_{bm} = 15\%$ and E: BDG vs time for lumbar vertebra, i.e. $f_{bm} = 25\%$, for a once-weekly 70 mg dose. In images A and B the three curves are practically overlapping.

References

[1] Ardawi, M., Al-Kadi, H., Rouzi, A., and Qari, M. (2011). Determinants of serum sclerostin in healthy pre- and postmenopausal women. *Journal of Bone Mineral Research* 26, 2812–2822. doi:10.1002/jbmr.479

[2] Beaupre, G., Orr, T., and Carter, D. (1990). An approach for time-dependent bone modeling and remodeling. *Journal of Orthopaedics Research* 8, 551–661. doi:10.1002/jor.1100080506

[3] Buenzli, P., Pivonka, P., Gardiner, B., and Smith, D. (2012). Modelling the anabolic response of bone using a cell population model. *Journal of Theoretical Biology* 307, 42–52. doi:10.1016/j.jtbi.2012.04.019

[4] Cardoso, L., Fritton, S., Gailani, G., Benalla, M., and Cowin, S. (2013). Advances in assessment of bone porosity, permeability and interstitial fluid flow. *Journal of Biomechanics* 46, 253–265. doi:10.1016/j.jbiomech.2012.10.025

[5] Chae, J., Seo, J., Mahat, B., Yun, H., Baek, I., Lee, B., et al. (2014). A simple pharmacokinetic model of alendronate developed using plasma concentration and urine excretion data from healthy men. *Drug Development and Industrial Pharmacy* 40, 1325–1329. doi:10.3109/03639045.2013.819880

[6] Cremers, S., Sparidans, R., Den, H. J., Hamdy, N., Vermeij, P., and Papapoulos, S. (2002). A pharmacokinetic and pharmacodynamic model for intravenous bisphosphonate (pamidronate) in osteoporosis. *European Journal of Clinical Pharmacology* 57, 883–890. doi:10.1007/s00228-001-0411-8

[7] Currey, J. (2004). Tensile yield in compact bone is determined by strain, postyield behaviour by mineral content. *Journal of Biomechanics* 37, 549–556. doi:10.1016/j.jbiomech.2003.08.008

[8] Eriksen, E., Hodgson, S., Eastell, R., Cedel, S., O'Fallon, W., and Riggs, B. (1990). Cancellous bone remodeling in type i (postmenopausal) osteoporosis: quantitative assesment of rates of formation, resorption and bone loss at tissue and cellular levels. *Journal of Bone Mineral Research* 5, 311–319. doi:10.1002/jbmr.5650050402

[9] Fisher, J., Rodan, G., and Reszka, A. (2000). In *vivo* effects of bisphosphonates on the osteoclast mevalonate pathway. *Endocrinology* 141, 4793–4796. doi:10.1210/endo.141.12.7921

[10] Fleisch, H. (2000). *Bisphosphonates in Bone Disease-From the Laboratory to the Patient* (New York: Academic Press)

[11] García-Aznar, J., Rueberg, T., and Doblaré, M. (2005). A bone remodelling model coupling microdamage growth and repair by 3d bmu-activity. *Biomechanics and Modelling in Mechanobiology* 4, 147–167. doi:10.1007/s10237-005-0067-x

[12] Halasy-Nagy, J., Rodan, G., and Reszka, A. (2001). Inhibition of bone resorption by alendronate and risedronate does not require osteoclast apoptosis. *Bone* 29, 553–559. doi:10.1016/s8756-3282(01)00615-9

[13] Hernández, C., Beaupre, G., and Carter, D. (2001). A model of mechanobiologic and metabolic influences on bone adaptation. *Journal of Rehabilitation Research and Development* 37, 235–244

[14] Hernández, C., Beaupre, G., Keller, T., and Carter, D. (2001). The influence of bone volume fraction and ash fraction on bone strength and modulus. *Bone* 29, 74–78. doi:10.1016/s8756-3282(01)00467-7

[15] Jacobs, C. (1994). *Numerical simulation of bone adaptation to mechanical loading* (Ph.D. thesis: Stanford University)

[16] Jastrzebski, S., Kalinowski, J., Stolina, M., Mirza, F., Torreggiani, E., Kalajzic, I., et al. (2013). Changes in bone sclerostin levels in mice after ovariectomy vary independently of changes in serum sclerostin levels. *Journal of Bone Mineral Research* 28, 618–626. doi:10.1002/jbmr.1773

[17] Juvinall, R. (1967). *Engineering Considerations of Stress, Strain and Strength* (New York, USA: McGraw-Hill)

[18] Kang, H., Hwang, S., Park, J., Kim, C., and et al. (2006). HPLC method validation and pharmacokinetic study of alendronate sodium in human urine with fluorescence detection. *Journal of Liquid Chromatography & Related Technologies* 29, 1589–1600. doi:10.1080/10826070600678308

[19] Khan, S., Kanis, J., Vasikaranand, S., Kline, W., Matuszewski, B., McCloskey, E., et al. (1997). Elimination and biochemical responses to intravenous alendronate in postmenopausal osteoporosis. *Journal of Bone and Mineral Research* 12, 1700–1707. doi:10.1359/jbmr.1997.12.10.1700

[20] Lemaitre, J. and Chaboche, J. (1990). *Mechanics of Solid Materials* (Cambridge, UK: Cambridge University Press)

[21] Lin, J., Duggan, D., Chen, I., and Ellsworth, R. (1991). Physiological disposition of alendronate, a potent anti-osteolytic bisphosphonate, in laboratory animals. *Drug Metabolism & Disposition* 19, 926–932

[22] Martin, M., Sansalone, V., Cooper, D. M. L., Forwood, M., and Pivonka, P. (2019). Mechanobiological osteocyte feedback drives mechanostat regulation of bone in a multiscale computational model. *Biomechanics and Modeling in Mechanobiology* 18, 1475–1496. doi:10.1007/s10237-019-01158-w

[23] Martin, R. (1984). Porosity and specific surface of bone. *Critical Reviews in Biomedical Engineering* 10, 179–222

[24] Martínez-Reina, J., García-Aznar, J., Domínguez, J., and Doblaré, M. (2008). On the role of bone damage in calcium homeostasis. *Journal of Theretical Biology* 254, 704–712. doi:10.1016/j.jtbi.2008.06.007

[25] Martínez-Reina, J., Calvo-Gallego, J., Martin, M., and et al. (2022). Assessment of strategies for safe drug discontinuation and transition of denosumab treatment in PMO -Insights from a mechanistic PK/PD model of bone turnover. *Frontiers in Bioengineering and Biotechnology* In Press

[26] Martínez-Reina, J., Calvo-Gallego, J., and Pivonka, P. (2021). Are drug holidays a safe option in treatment of osteoporosis?-insights from an in silico mechanistic PK-PD model of denosumab treatment of postmenopausal osteoporosis. *Journal of the Mechanical Behaviour of Biomedical Materials* 113, 104140. doi:10.1016/j.jmbbm.2020.104140

[27] Martínez-Reina, J., Calvo-Gallego, J., and Pivonka, P. (2021). Combined effects of exercise and denosumab treatment on local failure in

post-menopausal osteoporosis–insights from bone remodelling simulations accounting for mineralisation and damage. *Frontiers in Bioengineering and Biotechnology* 9, 635056. doi:10.3389/fbioe.2021.635056

[28] Martínez-Reina, J., García-Aznar, J., Domínguez, J., and Doblaré, M. (2009). A bone remodelling model including the directional activity of bmus. *Biomechanics and Modelling in Mechanobiology* 8, 111–127. doi:10.1007/s10237-008-0122-5

[29] Martínez-Reina, J. and Pivonka, P. (2019). Effects of long-term treatment of denosumab on bone mineral density: insights from an in-silico model of bone mineralization. *Bone* 125, 87–95. doi:10.1016/j.bone.2019.04.022

[30] Nakashima, T., Hayashi, M., Fukunaga, T., Kurata, K., Oh-Hora, M., Feng, J., et al. (2011). Evidence for osteocyte regulation of bone homeostasis through rankl expression. *Nature Medicine* 17, 1231. doi:10.1038/nm.2452

[31] Need, A., Horowitz, M., Morris, H., Moore, R., and Nordin, C. (2007). Seasonal change in osteoid thickness and mineralization lag time in ambulant patients. *Journal of Bone Mineral Research* 22, 757–761. doi:10.1359/jbmr.070203

[32] Parfitt, A. (2002). Targeted and nontargeted bone remodeling: relationship to basic multicellular unit origination and progression. *Bone* 30, 5–7. doi:10.1016/s8756-3282(01)00642-1

[33] Pattin, C., Caler, W., and Carter, D. (1996). Cyclic mechanical property degradation during fatigue loading of cortical bone. *Journal of Biomechanics* 29, 69–79. doi:10.1016/0021-9290(94)00156-1

[34] Peterson, M. and Riggs, M. (2010). A physiologically based mathematical model of integrated calcium homeostasis and bone remodeling. *Bone* 46, 49–63. doi:10.1016/j.bone.2009.08.053

[35] Pivonka, P., Buenzli, P., and Dunstan, C. (2012). *A Systems Approach to Understanding Bone Cell Interactions in Health and Disease* (USA: InTech). 169–204. doi:10.5772/51149

[36] Pivonka, P., Buenzli, P., Scheiner, S., Hellmich, C., and Dunstan, C. (2013). The influence of bone surface availability in bone remodelling — a mathematical model including coupled geometrical and biomechanical regulations of bone cells. *Engineering Structures* 47, 134–147. doi:10.1016/j.engstruct.2012.09.006

[37] Pivonka, P., Zimak, J., Smith, D., Gardiner, B., Dunstan, C., Sims, N., et al. (2008). Model structure and control of bone remodeling: A theoretical study. *Bone* 43, 249–263. doi:10.1016/j.bone.2008.03.025

[38] Porras, A. G., Holland, S. D., and Gertz, B. J. (1999). Pharmacokinetics of alendronate. *Clinical pharmacokinetics* 36, 315–328. doi:10.2165/00003088-199936050-00002

[39] Rizzoli, R., Greenspan, S., 3rd, G. B., Schnitzer, T., Watts, N., Adami, S., et al. (2002). Two-year results of once-weekly administration of alendronate 70 mg for the treatment of postmenopausal osteoporosis. *Journal of Bone Mineral Research* 17, 1988–1996. doi:10.1359/jbmr.2002.17.11.1988

[40] Sato, M., Grasser, W., Endo, N., Atkins, R., Simmons, H., Thompson, D., et al. (1991). Bisphosphonate action. alendronate localization in rat bone and effects on osteoclast ultrastructure. *Journal of Clinical Investigation* 88, 2095–2105. doi:10.1172/JCI115539

[41] Takagi, Y., Inoue, S., Fujikawa, K., Matsuki-Fukushima, M., Mayahara, M., Endo, Y., et al. (2021). Effect of nitrogen-containing bisphosphonates on osteoclasts and osteoclastogenesis: an ultrastructural study. *Microscopy* 70, 302–307. doi:10.1093/jmicro/dfaa073

[42] Ulrich, D., van Rietbergen, B., Laib, A., and Ruegsegger, P. (1999). The ability of three-dimensional structural indices to reflect mechanical aspects of trabecular bone. *Bone* 25, 55–60. doi:10.1016/s8756-3282(99)00098-8

[43] Valentín, J. (2002). Basic anatomical and physiological data for use in radiological protection: reference values. *Annals of the ICRP* 32, 1–277. doi:10.1016/S0146-6453(03)00002-2

[44] Xiong, J., Piemontese, M., Onal, M., Campbell, J., Goellner, J., Dusevich, V., et al. (2015). Osteocytes, not osteoblasts or lining cells, are the

main source of the rankl required for osteoclast formation in remodeling bone. *PLOS ONE* 10, e0138189. doi:10.1371/journal.pone.0138189

[45] Yu, Z., Surface, L. E., Park, C. Y., Horlbeck, M., Wyant, G., Abu-Remaileh, M., et al. (2018). Identification of a transporter complex responsible for the cytosolic entry of nitrogen-containing bisphosphonates. *eLIFE* 7, e36620. doi:10.7554/eLife.36620.001

[46] Zioupos, P. and Currey, J. (1998). Changes in the stiffness, strength, and toughness of human cortical bone with age. *Bone* 22, 57–66. doi:10.1016/s8756-3282(97)00228-7
